# Prevalence of Chagas Disease in Latin-American Migrants Living in Europe: A Systematic Review and Meta-analysis

**DOI:** 10.1371/journal.pntd.0003540

**Published:** 2015-02-13

**Authors:** Ana Requena-Méndez, Edelweiss Aldasoro, Elisa de Lazzari, Elisa Sicuri, Michael Brown, David A. J. Moore, Joaquim Gascon, Jose Muñoz

**Affiliations:** 1 ISGlobal, Barcelona Center for International Health Research, (CRESIB), Hospital Clínic—Universitat de Barcelona, Barcelona, Spain; 2 Department of Clinical Research, Faculty of Infectious and Tropical Diseases, London School of Hygiene and Tropical Medicine, London, United Kingdom; Federal University of São Paulo, BRAZIL

## Abstract

**Background:**

Few studies have assessed the burden of Chagas disease in non-endemic countries and most of them are based on prevalence estimates from Latin American (LA) countries that likely differ from the prevalence in migrants living in Europe. The aim of this study was to systematically review the existing data informing current understanding of the prevalence of Chagas disease in LA migrants living in European countries.

**Methods:**

We conducted a systematic review and meta-analysis of studies reporting prevalence of Chagas disease in European countries belonging to the European Union (EU) before 2004 in accordance with the MOOSE guidelines and based on the database sources MEDLINE and Global Health. No restrictions were placed on study date, study design or language of publication. The pooled prevalence was estimated using random effect models based on DerSimonian & Laird method.

**Results:**

We identified 18 studies conducted in five European countries. The random effect pooled prevalence was 4.2% (95%CI:2.2-6.7%); and the heterogeneity of Chagas disease prevalence among studies was high (I2 = 97%,p<0.001). Migrants from Bolivia had the highest prevalence of Chagas disease (18.1%, 95%CI:13.9–22.7%).

**Conclusions:**

Prevalence of Chagas in LA migrants living in Europe is high, particularly in migrants from Bolivia and Paraguay. Data are highly heterogeneous dependent upon country of origin and within studies of migrants from the same country of origin. Country-specific prevalence differs from the estimates available from LA countries. Our meta-analysis provides prevalence estimates of Chagas disease that should be used to estimate the burden of disease in European countries.

## Author Summary

Chagas disease is emerging in European countries due to the migration flows from Latin-American endemic countries to Europe, particularly to southern countries. Some studies have evaluated the prevalence of this disease in several European countries although these estimates are based on national population prevalence rates from countries of origin and the estimated size of the corresponding migrant population. The objective of this study is to review the studies about the prevalence of Chagas disease in Latin American migrants living in European countries. Our meta-analysis provides prevalence estimates of Chagas disease that should be used to estimate the burden of disease in European countries. This accurate data about country specific prevalence of Chagas disease could be used to evaluate the cost-effectiveness of screening programmes and also could help policy makers to design health interventions concerning Chagas disease.

## Introduction

One of the most remarkable changes in the epidemiology of parasitic diseases in recent decades has been the emergence of Chagas disease in European countries and its associated transmission risk outside of endemic areas. Europe is currently hosting large populations of migrants that were estimated to account for the 8.7% of the total European population in 2010[1]. Migration from Latin American (LA) countries steadily increased in the last two decades, especially in southern European countries such as Spain and Italy[2,3] and more recently in further northern European countries[4].

These population movements have driven the emergence of Chagas disease in European countries[2,3] since a considerable percentage of LA migrants are chronically infected with *Trypanosoma cruzi (T.cruzi)*. Consequently, the number of reported cases of Chagas disease with and without cardiac involvement has dramatically increased in recent years especially in those European countries such as Spain, Italy and Switzerland where most LA migrants are hosted[5–7]. Though vector-borne transmission cannot occur in Europe (because the vector is not present), Chagas disease can be transmitted in non-endemic countries through vertical transmission, blood transfusion and organ transplantation. Measures to control transmission have been designed and implemented in some countries in order to mitigate the risk of propagating the disease within European borders[8–10], although these measures have been shown to be insufficient.

The clinical importance of Chagas disease arises from the 30–40% of infected patients that will develop cardiac and gastrointestinal involvement many years after infection[11]. Though a significant proportion remain asymptomatic (in the so-called “indeterminate” stage of the infection), they may be capable of transmitting the infection to others. Although digestive tract disorders can be a severe health problem in chronically infected individuals, arrhythmias and severe cardiomyopathy constitute the hallmark of the chronic phase and the most common cause of death in people who die from Chagas disease[11]. There is no vaccine available to prevent *T.cruzi* infection or Chagas disease. Antiparasitic therapy has proven efficacy in clearing *T.cruzi* infection in acute, congenital and early chronic disease[12–16], and although there is a trend to offer antiparasitic therapy to chronically infected adults[17], the efficacy of this moderately toxic and poorly tolerated treatment in this stage of the disease remains to be fully evaluated.

Assessing the true burden and public health implications of Chagas disease in European countries is crucial for the design and planning of public health interventions to improve the health of migrants in Europe and to control transmission. A study in 2009 based on aggregated data collected from the literature and official sources estimated the total number of people infected with *T.cruzi* in European countries as between 68,000 and 122,000 cases, with the greatest numbers believed to be living in Spain, Italy and the United Kingdom[3]. However, the study noted that only 4,290 cases had been reported, meaning that 95% of cases remained undiagnosed[3]. The fact that most chronically infected patients remain asymptomatic for long time[11], that health professionals in non-endemic areas are generally unaware of this disease, and that barriers to access healthcare for migrant populations are still present[1], greatly explains the high rate of underdiagnosis in European health care systems[18].

Available estimates of the burden of Chagas disease in Europe are derived by applying national population prevalence rates from countries of origin to the estimated size of the corresponding migrant population[2,3]. Because country of origin prevalence is geographically heterogeneous and because migrant populations may not be representative of the whole population of origin (geographically or socioeconomically)[19], we hypothesized that the prevalence of Chagas disease in LA migrants living in Europe differs from that reported in LA countries.

The main objective of this study was to establish prevalence estimates of Chagas disease in LA migrants living in European countries. We systematically reviewed all *T.cruzi* prevalence studies undertaken in (i) EU/EEA countries, (ii) in the adult population and (iii) non-hospital based and we performed a meta-analysis to estimate the global prevalence of Chagas disease in Europe.

## Methods

### Search string and selection criteria

A systematic review was undertaken in accordance with the MOOSE guidelines as outlined by the Meta-analysis of Observational Studies in Epidemiology group[20] in order to identify all relevant publications reporting prevalence of Chagas disease in European countries belonging to the European Union (EU) before 2004. Countries included were Spain, Portugal, France, Italy, Germany, Belgium, Netherlands, United Kingdom, Denmark, Austria, Greece, Ireland, Sweden and Finland. Switzerland was also included for the purpose of this study due to the high number of Latin American migrants as well as the increasing number of confirmed cases of *T. cruzi* infection in that country^6^. Countries joining the EU after 2004 were not included because of the extremely low number of Latin American migrants and scarce or non-existent data about Chagas disease in these countries.

The search strategy was used to interrogate the database sources MEDLINE and Global Health. Other sources of information used included conference proceedings, meeting abstracts, Masters and Doctoral Theses, personal correspondence with authors of recently published abstracts and manuscripts in press.

In the search string, the results were limited to all articles published up to December 2013. The following search terms were used (Chagas OR Trypanosom*) AND (Europe OR Spain OR France OR United Kingdom OR Germany OR Belgium OR Switzerland OR Portugal OR Italy OR Netherlands OR Sweden OR Finland OR Denmark OR Greece OR Austria OR Ireland). The last update search was performed in January 2014.

No restrictions were placed on study date, study design or language of publication. Prevalence studies based on community, antenatal care, blood banks or primary health care were included since participants’ health seeking was not likely to be specifically motivated towards Chagas disease diagnosis, whereas hospital-based studies were not included because of the high risk of selection bias. Moreover, studies assessing the prevalence of Chagas disease using different criteria to those considered by WHO (to confirm the diagnosis, two conventional tests should be used. If the results are not in agreement, a third test should be performed, either conventional or non-conventional[21]) were not considered for inclusion in the meta-analysis. Prevalence studies that had not itemized the nationalities of screened people were also not included in our study.

### Data extraction

Two members of the study team assessed all selected documents for data extraction. Publications reporting survey data at the same location and period were carefully examined to avoid duplicate information.

Prevalence data from reference sources was extracted in accordance with a standard protocol. Data on geographical location, year of publication and duration of the study, age population, type of study, sampling methodology, methods of diagnosis of *T.cruzi* infection and prevalence of *T.cruzi* infection stratified by country of origin was included. In the case of blood-bank studies, prevalence data of *T.cruzi* on travelers was excluded. When country of origin data of all screened people were not completely detailed in the article, further clarifications from the authors of these studies were requested and in case this information was not available, these prevalence data were labeled as “other countries” only for the analysis of the pooled prevalence data.

Disagreement was resolved by consensus between the two reviewers or through consultation with the corresponding author of the selected papers, when necessary. Study quality was assessed considering MOOSE guidelines.

### Statistical methods

Pooled prevalence estimates with corresponding 95% confidence intervals (CI) were calculated using the Freeman-Tukey double arcsine transformation. Random effects model based on the DerSimonian & Laird method was considered. Between studies heterogeneity was assessed using the Cochran’s Q statistic. The I^2^ index was also reported, indicating the variation between studies attributed to heterogeneity rather than chance. Stratified analysis by type of study was conducted with data from countries that showed high heterogeneity index. In case of no statistically significant heterogeneity, the pooled prevalence was estimated using the fixed effect model based on the inverse variance. The nature of the data of this review was the point prevalence estimates without any intervention, thus the publication bias was expected to be negligible. With the forest plot we displayed the point prevalence and 95% CI of individual studies as well as the pooled estimates and 95% CI for all strata. All tests were two-tailed. The analyses were performed using Stata 13 (StataCorp. 2013. Stata: Release 13. Statistical Software. College Station, TX: StataCorp LP) and StatsDirect 2.8.0 (StatsDirect,Altrincham, Cheshire, UK).

All funding sources had no role in study design, data collection, analysis, interpretation, or writing of the report. All authors had full access to the study data and had final responsibility for the decision to submit for publication.

## Results

### Study selection

**Fig 1:**
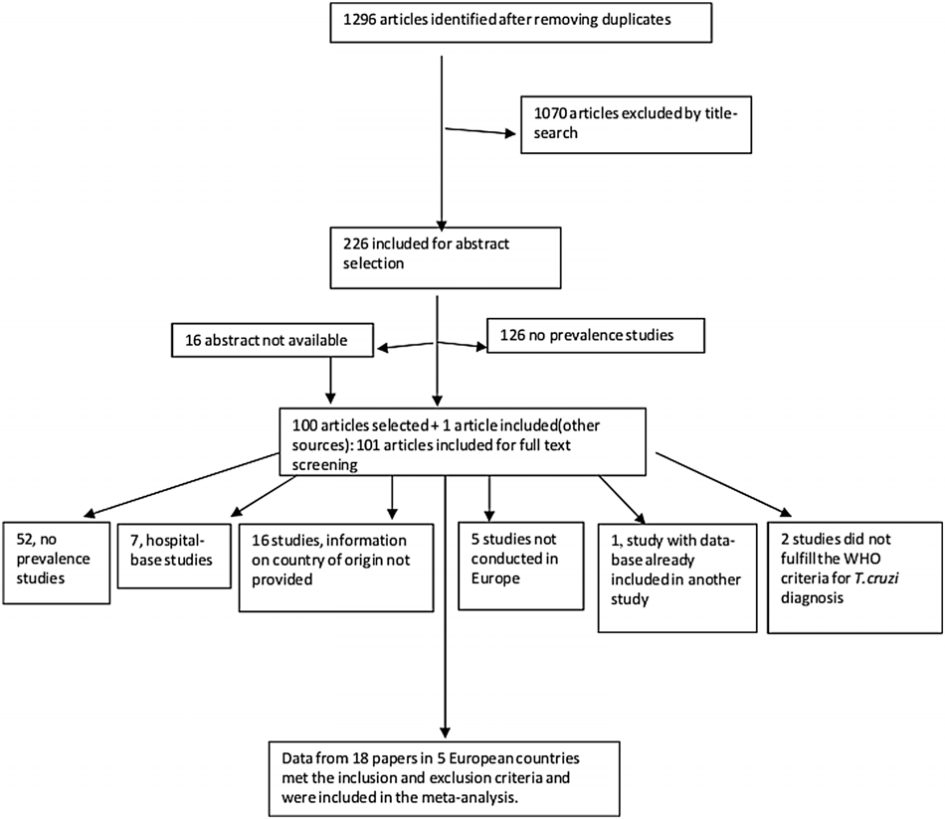
Flow diagram for study selection.

A list of all the articles retrieved from the literature search is available in the S1 Text. After removal of duplicates 1296 potentially eligible articles were identified by title-search. 1070 irrelevant articles were eliminated by title-search because they were not related to Chagas disease or to the epidemiology of the disease and 226 potentially relevant articles were accepted for review of the abstract. Of these, 126 did not contain prevalence data leaving a total of 100 papers (including 16 papers which did not contain any abstract) plus one study which was introduced after reviewing the references of other article) that were retrieved for full-text screening. Out of them, 52 were rejected since they did not provide any data of prevalence, 7 papers were further rejected since they were hospital-based studies, 16 papers were ruled out since the information about country of origin of all screened people was not provided; 5 articles were excluded because the study had not been conducted in Europe, 1 article because the data-base had been already included in a another study, 2 articles because the inclusion criteria did not full-fit the WHO criteria for the diagnosis of Chagas disease. Finally, data from 18 papers were finally included in the meta-analysis (Fig. 1).

Three studies were undertaken in blood banks,[22–24] eight in antenatal units,[8,25–31] six in primary care settings or communities[6,32–36] and one study provided data both from blood bank and antenatal settings.[37] Data from 10,884 individuals was analyzed. Data were only available from studies conducted in five European countries: Spain (n = 8,148), France (n = 972), Switzerland (n = 1,317), Italy (n = 347) and Germany (n = 100). A summary of the included studies is provided in Table 1.

**Table 1:** Main characteristics of the studies included in the meta-analysis.

Author	Country	Study Year	Study type	Sampling characteristics	Age	Diagnostic method	Prevalence (%)	n	Reference
Angheben	Italy	2008-? / 2008–2009	Blood bank/ Antenatal care	Prospective general screening. No active community outreach	Adult	Immunochromatographic assay (Chagas Quick Test, Cypress Diagnostics, Belgium); BioELISA Chagas, Biokit S.A., Spain; DRG CHAGAS IgG, Germany	0	245	[37]
Avila-Arzanegui	Spain	Dec 2008—Jan 2010	Antenatal care	Prospective general screening. No active community outreach	Adult	Indirect immunofluorescence (MarDx, Inc. Trinity Biotech plc Bray, Co. Wicklow, Ireland); ELISA *T. cruzi* Ab, Dia. Pro, Milán, Italy); ORTHO T. cruzi ELISA Test System, Buckinghamshire, UK.	12	158	[25]
Barona-Vilar	Spain	2009–2010	Antenatal care	Multicentre retrospective cross-sectional survey of microbiological records	Adult	Ortho1 T. cruzi ELISA, Test System, (Johnson & Johnson, USA); Chagatek ELISA, (bioMerieux, France) and *T. cruzi* Ab, DIAPRO, (Diagnostic BioProbes, Italy); Agglutination assay, IDPaGIA Chagas, DiaMed AG, Switzerland; Immunochromatographic assay; Stick Chagas, Operon SA, Spain	11.6	1945	[26]
El Ghouzzi	France	April 2007- Oct 2008	Blood bank	Prospective general screening. No active community outreach	Adult	ELISA cruzi, manufactured by bioMérieux Brazil S.A (Estrada do Mapuã, Jacarepaguã, RJ, Brazil); Bioelisa CHAGAS (Biokit, Lliçà d’Amunt, Spain)	0.3	972	[22]
Frank	Germany	May—August 1995	Community study	Religious support group of Latin Americans recruited at their meetings. Informed consent sought.	Adult	Indirect immunofluorescence test (IIF) using fixed epimastigotes (Bits/Germany) as antigen; In house ELISA for *T. cruzi* antibodies	2	100	[32]
Gabrielli	Italy	2010–2012	Blood bank	Prospective general screening. No active community outreach	Adult	Immunochromatographic assay (ICT) (Chagas Quick Test, Cypress Diagnostics, Langdorp, Belgium; BioELISA Chagas, Biokit S.A., Barcelona, Spain; NovaLisa Chagas ELISA test, Nova Tec Immunodiagnostica, GmbH, Dietzenbach, Germany	0.98	102	[23]
Jackson	Switzerland	Jun- Dec 2008	Primary health care and community study	Prospective recruitment in primary care unit. Community outreach: active case finding in cultural centres, churches and migrant associations. Recruitment sessions in churches attended by migrants.	>16 y	ELISA cruzi, Biomérieux, Brazil and Bioelisa Chagas, Biokit, Spain),	12.85	1012	[6]
Orti-Lucas	Spain	Feb 2005—Jul 2007	Antenatal care	Prospective general screening. No active community outreach	Adult	Immunoprecipitación particle gel immuno assay—Diamed (IP) (reference BO20011–01.04); Indirect immunofluorescence, Immunoflour Chagas—Inverness Medical (reference 20–03648; ELISA Dade Behering (reference CHAG0560DB)	9.69	382	[27]
Martinez de Tejada	Switzerland	2008	Antenatal care	Prospective general screening. No active community outreach	Adult	Immunofluorescence in house	1.97	305	[28]
Muñoz-Vilches	Spain	April 2007—April 2011	Antenatal care	Prospective general screening. No active community outreach	Adult	ELISA-in house (ELISA-CNM Centro Nacional de Microbiología-); Indirect immunofluorescence assay, IFI-in house (IFI-CNM)	1.53	261	[29]
Muñoz	Spain	March 2005—Sept 2007	Antenatal care	Prospective general screening. No active community outreach	Adult	BioELISA Chagas; Biokit S.A, Spain; In house ELISA (crude antigen from *T. cruzi* epimastigotes)	3.41	1350	[8]
Navarro	Spain	May 2008—Dec 2009	Community study	Non-governmental organisations and migrants’ associations promoted talks on the disease to migrants in different community settings.	Adult	Rapid immunochromatographic test (Simple Chagas WB, Operon); Filter paper sent to the National Microbiology Centre for confirmation: using Indirect fluorescent antibody technique and ELISA assay.	15.94	276	[33]
Patricio Talayero	Spain	2005–2007	Antenatal care	Prospective general screening No active community outreach	Adult	Immunoprecipitation, ID-PaGIA Chagas Antibody Test, (reference B020011–01.04 de Diamed-Id); Indirect immunofluorescence (Innogenetics, ref. 20–03648)	4.65	624	[30]
Piron	Spain	Sept 2005—Sept 2006	Blood bank	Prospective general screening. No active community outreach	Adult	Particle gel immunoassay (ID-PaGIA, DiaMed, Cressier surMorat, Switzerland); Chagas bioelisa assay (Biokit, Lliçá d’Amunt, Spain)	0.66	1770	[24]
Ramos_a_	Spain	2006–2010	Antenatal	Prospective general screening. No active community outreach	Adult	Particle gel immunoassay (ID-PaGIA, DiaMed, Cressier surMorat, Switzerland); Chagas bioelisa assay (Biokit, Lliçá d’Amunt, Spain)	1.28	545	[31]
Ramos_b_	Spain	Nov 2009—Nov 2010	Community study	Informal links with migrants’ and migrants’ associations through social and cultural activities to deliver information and conduct recruitment	Adult	In-house ELISA (antigen prepared from epimastigotes obtained from a culture of the stationary phase of two strains of *T. cruzi*, (MC and T) and from another *T. cruzi* I (Dm28); In-house Indirect immunofluorescent antibody test (antigens prepared from cultures of epimastigotes in the stationary phase of the same strains of *T. cruzi* used in ELISA).	6.47	201	[34]
Roca	Spain	Oct 2007—Oct 2009	Primary Health care	Prospective general screening. No active community outreach	Adult	Immunochromatographic test (ICT) that uses recombinant antigens of *T. cruzi* (TcD, TcE, PEP-2 and SAPA); In-house ELISA with whole *T. cruzi* epimastigote antigens; Commercial ELISA (recombinant antigens TcD, TcE, PEP-2 and TcLo1.2.)	2.87	766	[35]
Soriano	Spain	March 2006—March 2007	Primary Health Care	Prospective general screening. No active community outreach	15–45 y	Chagas bioelisa assay (Biokit, Lliçá d’Amunt, Spain); In-house ELISA (epimastigote antigens obtained by sonication from the epimastigote forms of the Maracay strains of *T. cruzi*)	4.31	116	[36]

### Country of origin data by study type

Estimates of pooled Chagas disease prevalence in publications ranged from 0 to 15.9%. The random effect pooled prevalence was 4.2% (95% CI: 2.2–6.7%); and the heterogeneity of Chagas disease prevalence among studies included in the meta-analysis (with widely varied population groups originating from 14 different countries) was very high (I^2^ = 97%, p<0.001). Prevalence estimates from studies conducted in blood bank studies (0.5%, 95%: 0.3–0.9%) were considerably lower than those derived from primary health care or community level (8.7%, 95%CI: 7.7–9.9%) and antenatal screening (6.5%, 95% CI: 5.8–7.1%) settings (p<0.001, Table 2). These differences were highest in data from Argentina (I^2^ 72.4%) and Bolivia (I^2^ 87.4%). Stratified analysis by type of study was conducted with data from these two countries showing high heterogeneity within PHC/community and antenatal studies. Studies conducted in blood banks showed more homogeneous prevalence estimates (Table 3).

**Table 2:** Heterogeneity of pooled prevalence estimates of Chagas disease by type of study recruitment setting.

	Positive	Screened	Prevalence (%)	95% CI.	% weight (random)
PHC	216	2471	8.7	7.7–9.9	33.27
blood donors	14	2629	0.5	0.3–0.9	33.29
pregnancy	374	5784	6.5	5.8–7.1	33.43
Random Effects Pooled Prevalence	604	10884	4.2	0.7–10.8	100.00

TEST FOR HETEROGENEITY

Q Heterogeneity chi-squared = 319.05 (d.f. = 2) p = 0·0000

I^2^ (variation in Prevalence attributable to heterogeneity) = 99.4%

Moment-based estimate of between-study variance Tau^2^ = 0.0479

**Table 3:** Country-specific prevalence of Chagas disease by type of study and values of results of heterogeneity tests among different type of studies.

	Prevalence PHC*/Community studies (95% CI)	Prevalence Blood bank studies (95% CI)	Prevalence Antenatal studies (95% CI)	HeterogeneityI^2^	p-value (Q Heterogeneity chi-squared)
**Argentina**	2.6 (0.3–9.2) ^1^	0.5 (0.1–1.9) ^2^	2.8(1.5–4.9) ^3^	72.4	0.03
**Bolivia**	21.6 (19–24.4)^4^	10.7 (4.7–19. 9)^5^	26.5 (24–29)^6^	87.4	<0.001
**Brazil**	0.4 (0–2)	0 (0–1.1)	0.9 (0.2–2.6)	46.4	0.2
**Chile**	0 (0–0.1)	0 (0–2.1)	1.3 (0–6.8)	5.3	0.3
**Colombia**	0 (0–1.9)	0 (0–0.7)	0.7 (0.2–1.4)	65.5	0.06
**Ecuador**	0 (0–1.3)	0.4 (0–2.2)	0.4 (01–0.8)	0	0.5
**El Salvador**	0 (0–30.8)	9.1 (1.1–29.2)	0 (0–0.1)	43.8	0·2
**Honduras**	0 (0–9.7)	0 (0–21.8)	3.5 (0.7–10)	.0	0.4
**Mexico**	0	0	0	-	-
**Nicaragua**	0 (0–14.2)	0 (0–28.5)	6.7 (0.2–31.9)	0	0.4
**Paraguay**	3.9 (1.6–7.8)	4.5 (0.1–22.8)	6 (3.1–10.6)	0	0.7
**Peru**	0.3 (0–1.9)	0 (0–1.6)	0.6 (0.1–1.7)	0	0.4
**Uruguay**	0	0	0	-	-
**Venezuela**	0	0	0	-	-

95% CI—95% confidence interval; All country test for heterogeneity: Q Heterogeneity chi-squared = 319.1 (d.f. = 2), p< 0. 001, I^2^ (variation in Prevalence attributable to heterogeneity) = 99.4%, Moment-based estimate of between-study variance Tau^2^ = 0.05;

^1^: I^2^ index of heterogeneity 79.5%;

^2^: I^2^ index of heterogeneity 0%;

^3^: I^2^ index of heterogeneity 51.5%;

^4^: I^2^ index of heterogeneity 79.4%;

^5^: I^2^ index of heterogeneity 0%;

^6^: I^2^ index of heterogeneity 85.1%

### Pooled migrant data by country of origin (regardless of setting)

**Fig 2:**
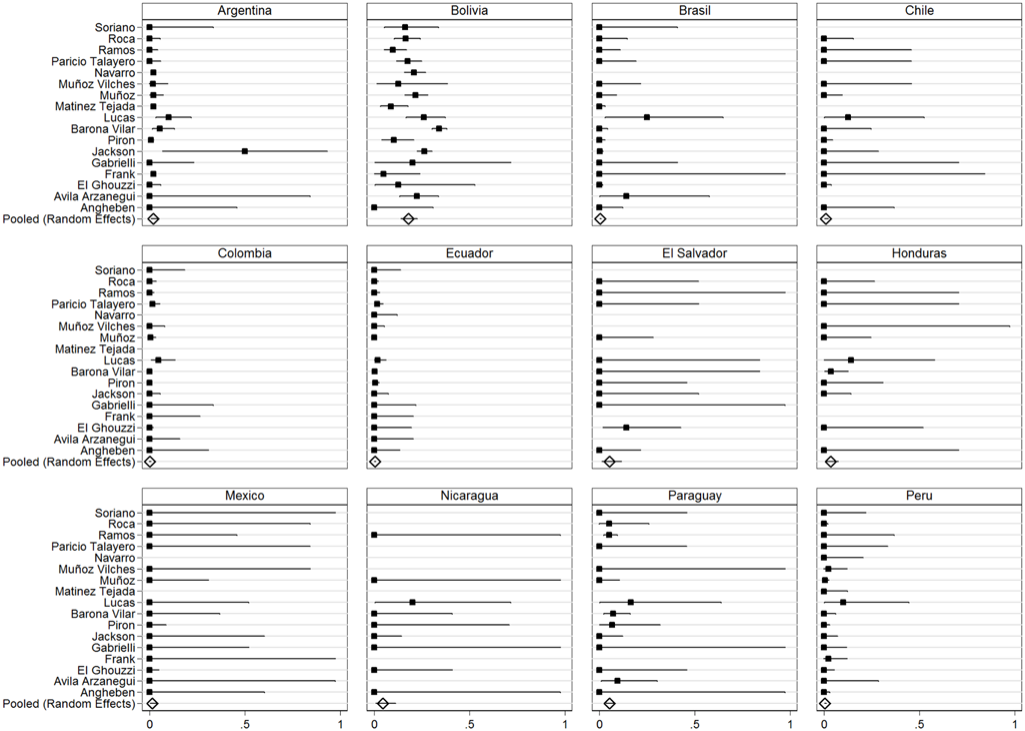
Forest plots of prevalence of Chagas disease by country of origin of Latin American migrants.

Chagas disease prevalence estimates varied substantially by country of origin (Table 3). Migrants to Europe from Bolivia had the highest prevalence of Chagas disease (18.1%, 95% CI: 13.9–22.7), followed by those from Paraguay (5.5%, 95% CI: 3.5–7.9). Individuals born in Central American countries that showed high prevalence of Chagas disease were from El Salvador (5.6%, 95% CI: 1.6–11.7), Honduras (3.7%, 95% CI: 1.3–7.4) and Nicaragua (4.57%, 95% CI: 0.8–11.3), although the number of screened subjects was very low (67, 136, 50 respectively) (Table 4). Prevalence amongst Argentinian migrants was 2.2% (95% CI: 0.8–4.1) and the prevalence amongst migrants from other countries in all studied groups was under 1% (Table 4). No cases of Chagas disease were detected in migrants to Europe from Uruguay, Venezuela, Panama, Guatemala or Mexico. Fig. 2 shows the results from random effects model pooled *T. cruzi* prevalence and heterogeneity by country of birth.

**Table 4:** Pooled *T. cruzi* prevalence by country of origin in Latin American migrants from European countries.

Country	Number screened	Number of seropositives	Country-specific prevalence* (%)	95% CI	Prevalence in country of origin (National level) PAHO (%)[39]	Prevalence ratio
**Argentina**	875	16	2.2	0.80–4·13	4.13	0.53
**Bolivia**	2264	541	18	13.9–22.66	6.75	2.67
**Brazil**	954	4	0.6	0.16–1.12	1.02	0.59
**Chile**	290	1	1	0.17–2.36	0.99	1.01
**Colombia**	1627	6	0.5	0.15–0.92	0.96	0.52
**Ecuador**	2131	7	0.4	0.18–0.72	1.74	0.23
**El Salvador**	67	2	3.7	1.62–11.7	3.37	1.10
**Honduras**	136	3	4.2	1.27–7.36	3.05	1.38
**Mexico**	166	0	1.5^^^	0.24–3.76	1.03	1.46
**Nicaragua**	50	1	4.6	0.76–11.3	1.14	4.04
**Paraguay**	385	19	5.5	3.46–7.91	2.54	2.17
**Peru**	1029	4	0.6	0.23–1.18	0.69	0.87
**Uruguay**	248	0	0.8^^^	0.08–2.24	0.66	1.21
**Venezuela**	311	0	0.9^^^	0.16–2.22	1.16	0.78

CI: Confidence Interval; PAHO: Pan American Health Organization;

*Weighted prevalence with Random effect model;

^^^ although there was not any reported case of Chagas disease in migrants coming from this country, the weighted prevalence is not “0” due to the Random Effect model

### Comparison of migrant data to country of origin data

When compared to PAHO estimates of national prevalence rates the pooled prevalence of Chagas disease in migrants from Bolivia and from Paraguay living in Europe were significantly higher (prevalence ratio 2.67 and 2.17 respectively). The prevalence observed in Central American countries was also higher than the official in-country estimates, albeit with large confidence intervals due to small sample sizes. Table 4 shows prevalence estimates from the meta-analysis compared to the available estimates from LA countries.

## Discussion

The true burden and public health implications of imported Chagas disease in European countries are still unclear, hampering the possibility of designing and implementing targeted public health interventions to improve the health of infected individuals and control the transmission in European countries. One of the main limitations to obtain accurate estimates is the uncertain prevalence of Chagas disease in the migrant populations in Europe, that likely differs from the estimates from LA countries and that is closely linked to the country and region of origin[19]. Data presented here indicate that overall 4.2% of LA individuals living in European countries are chronically infected with Chagas disease which suggests that European countries hosting LA migrant population should seriously evaluate the need to address the detection, management and control of Chagas disease. However, concealed within the overall point estimate of 4.2% is marked heterogeneity both between and within migrants from different LA countries. The data presented here are a useful starting point for understanding the burden of Chagas disease in migrants to Europe whilst also highlighting the profound knowledge gap and clear need for more systematic, larger scale investigation.

The high degree of prevalence heterogeneity found among different countries of origin was expected. Bolivian migrants had the highest prevalence of Chagas disease with 18% of the migrant population infected with *T.cruzi*. This is concordant with data from high endemic areas in Bolivia from 2004 to 2009, in which 23.3% of pregnant women were estimated to be infected with *T.cruzi*[38]. However, in comparison to PAHO estimates[39] of the prevalence of *T.cruzi* infection in the whole Bolivian population (6.75%), prevalence of the infection in migrants is 2.67 fold higher. Thus, extrapolating PAHO data to estimate the prevalence of Chagas disease in a particular European country would significantly underestimate the real burden of Chagas disease in Bolivian migrant populations. One possible explanation for this difference is the fact that an over-representative proportion of Bolivian migrants are coming from hyperendemic areas of Chagas disease in Bolivia such as the departments of Tarija, Cochabamba or Chuquisaca and the proportion of migrants coming from low endemic areas in Bolivia such as La Paz is lower. High variation in *T. cruzi* prevalence has also been described with pregnant women living in high risk areas such as Chuquisaca (37% infected) or Tarija (38%) being more than seven times more likely to be infected as compared with those living in low-endemic regions such as La Paz (5%)[38].

Similarly, migrants from Paraguay also presented higher prevalence of Chagas disease as compared to overall in-country estimates[39], but comparable with studies undertaken in hyperendemic areas (12.7%)[40]. This may reflect a higher proportion of people from Paraguay migrating from highly endemic areas to Europe as suggested for Bolivian migrants, or simply that migrants from high endemic areas are more likely to be screened than those coming from big cities. Our data show opposite results in migrants from Argentina, with lower prevalence (2.2%) as compared to the overall prevalence estimated in Argentina (4.13%). Again, this may be attributable to the demography of migrant populations, in this case perhaps predominantly migration from urban parts of this country.

Prevalence amongst migrants from other countries including Brazil, Peru, Colombia and Venezuela was around or lower than 1%. A somewhat surprisingly high pooled prevalence of Chagas disease was found in migrants coming from El Salvador, Honduras and Nicaragua, even though more data is needed to achieve accurate prevalence estimates for these populations, as sample size were not large. However, it is noteworthy that these data differ significantly from PAHO estimates, in the case of Nicaragua representing a four-fold increase. Although data on the prevalence of Chagas disease in Central America is scarce, a prevalence as high as 8,5% was found in a sample taken from patients in the area of Somoto in Nicaragua[41]. The heterogeneity and smaller size of migrant communities analysed in some studies, especially in the case of countries from Central America, with relatively little representation in European countries, represents a challenge to achieve statistically relevant information. On the other hand, our results may also question the validity of PAHO estimates, highlighting the need for updated prevalence data in LA countries.

Available data estimating the prevalence of Chagas disease in European countries are heavily reliant on research studies conducted in tertiary referral centres that tend to overestimate the true population prevalence[19]. This systematic review excluded hospital-based studies and studies conducted in reference units because of the risk of overestimating the prevalence due to selection bias. Studies conducted in antenatal care, blood banks, primary health care or community-based were included in the analysis. Even though the profile of participants differs from one type of study to another, we were not expecting large selection bias since in most cases the reason for attending the health setting was not to seek a diagnosis of Chagas disease. However, we found a high heterogeneity of prevalence by study type, showing higher estimates in studies conducted in the community and primary care and in antenatal care compared to those conducted in blood banks. This heterogeneity was mainly found in data from Bolivia and Argentina. Although it is possible that these differences are caused by the different recruitment settings, these studies could have overestimated the prevalence due to selection bias. Random selection methods were not used to recruit participants in any of the studies found in the literature search[6,32–34]. Unbiased community based prevalence data are needed to understand the true prevalence of disease. Moreover, self-exclusion from transfusion services due to pre-screening ineligibility could also be the reason for lower prevalence in blood-bank studies. Finally, migrants going to blood-banks maybe older, with longer stays in Europe and from different socio-economical background compared to migrants attending PHC centers or maternities, which could also explain in part the lower prevalence in blood-banks compared with the other settings.

Another limitation of this study is the fact that pooled estimates do not capture the heterogeneity that exists within some regions in LA countries. Thus, there is a need for additional sub-national-level data to make decisions about the implementation of screening and management programmes.

Updated data from LA migrants in Europe is needed taking into account (i) changes in migration flows, particularly in the context of the current economic crisis; (ii) undocumented migrants and (iii) LA migrants with European citizenship. Health information systems in most European countries currently lack capacity to analyze data based on migration status and/or country of origin. The unclear definition of who constitutes a migrant, and the use of different variables as a proxy for migration status (nationality, ethnicity, origin), including the lack of data about country of birth in many countries hampers the possibility to gather and compare data between countries[1].This is especially relevant in the case of Chagas disease, where large differences in prevalence are observed between different LA countries and even within different regions of the same country[5,11].

Providing policy makers in European countries with accurate data about country-specific prevalence of Chagas disease could help them in the design of health interventions concerning Chagas disease. Furthermore these differences acquire importance when the cost-effectiveness of screening programmes is assessed since variations in prevalence could alter the results of the economic analysis and provide policy makers with incorrect information.

## Supporting Information

S1 Text List of articles retrieved from the literature search. (DOCX)

S1 Checklist PRISMA Checklist.
(DOC)

S1 Diagram PRISMA Flow diagram.
(DOC)
