# Supplementary material for: Prevalence of Chagas Disease in Latin-American Migrants Living in Europe: A Systematic Review and Meta-analysis
Source: PLoS Negl Trop Dis. 2015 Feb 13;9(2):e0003540. doi: 10.1371/journal.pntd.0003540 (PMC4332678; doi:10.1371/journal.pntd.0003540)
Supplement: S1 Text — (DOCX) [file pntd.0003540.s001.docx]

S1 Text.

LIST OF ARTICLES RETRIEVED FROM THE LITERATURE SEARCH

***ARTICLES EXCLUDED BY TITLE-SEARCH***

1. [Chagas disease. American trypanosomiasis. Recommendation for non-endemic zones]. Med Trop (Mars) 70: 131-132.

2. (1965) Sleeping sickness presenting in Britain. Discussed at the Hospital for Tropical Diseases, London, N.W.1. J Trop Med Hyg 68: 296-299.

3. (1969) African trypanosomiasis--Florida. J Infect Dis 119: 307-308.

4. (1974) [World distribution of tropical diseases]. Nouv Presse Med 3: 2342-2343.

5. (1976) [Current data. Infectious diseases: monthly or four-weekly number of reported cases, 1974 and 1975]. World Health Stat Rep 29: 236-248.

6. (1977) Infections diseases: monthly or four-weekly number of reported cases, 1976 and 1977. World Health Stat Rep 30: 260-270.

7. (1979) Royal Society of Tropical Medicine and Hygiene. Nineteenth seminar on trypanosomiasis. Summaries of papers. Trans R Soc Trop Med Hyg 73: 125-139.

8. (1983) African trypanosomiasis. MMWR Morb Mortal Wkly Rep 32: 112-113.

9. (2002) Blue Ribbon Committee on Bloodborne Parasitic Diseases. Can Commun Dis Rep 28 Suppl 3: 1-23 (Eng), 21-27 (Fre).

10. (2002) From the Centers for Disease Control and Prevention. Chagas disease after organ transplantation--United States, 2001. JAMA 287: 1795-1796.

11. (2009) [Conclusion of the consensus workshop organized by the "Societe de pathologie exotique" about the Chagas disease in non-endemic areas (26 June 2009, Paris, France)]. Bull Soc Pathol Exot 102: 342-346.

12. Abd-Alla AM, Bergoin M, Parker AG, Maniania NK, Vlak JM, et al. Improving Sterile Insect Technique (SIT) for tsetse flies through research on their symbionts and pathogens. J Invertebr Pathol 2013 Mar;112 Suppl:S2-10 doi: 101016/jjip201207009 Epub 2012 Jul 24.

13. Abderrazak SB, Oury B, Lal AA, Bosseno MF, Force-Barge P, et al. (1999) Plasmodium falciparum: population genetic analysis by multilocus enzyme electrophoresis and other molecular markers. Exp Parasitol 92: 232-238.

14. Abila PP, Slotman MA, Parmakelis A, Dion KB, Robinson AS, et al. (2008) High levels of genetic differentiation between Ugandan Glossina fuscipes fuscipes populations separated by Lake Kyoga. PLoS Negl Trop Dis 2: e242.

15. Acha RE, Rezende MT, Guzman Heredia RA, Silva AC, Rezende ES, et al. (2002) Prevalence of cardiac arrhythmias during and after pregnancy in women with Chagas' disease without apparent heart disease. Arq Bras Cardiol 79: 1-9.

16. Acquatella H (2008) [Predicting heart failure and mortality in chronic Chagas' heart disease. A novel disorder in Spain]. Rev Esp Cardiol 61: 105-107.

17. Afewerk Y, Clausen PH, Abebe G, Tilahun G, Mehlitz D (2000) Multiple-drug resistant Trypanosoma congolense populations in village cattle of Metekel district, north-west Ethiopia. Acta Trop 76: 231-238.

18. Afonso AM, Ebell MH, Tarleton RL A systematic review of high quality diagnostic tests for Chagas disease. PLoS Negl Trop Dis 2012;6(11):e1881 doi: 101371/journalpntd0001881 Epub 2012 Nov 8.

19. Agbo EC, Clausen PH, Buscher P, Majiwa PA, Claassen E, et al. (2003) Population genetic structure and cladistic analysis of Trypanosoma brucei isolates. Infect Genet Evol 3: 165-174.

20. Akinwale OP, Nock IH, Esievo KA, Edeghere HU, Olukosi YA (2006) Study on the susceptibility of Sahel goats to experimental Trypanosoma vivax infection. Vet Parasitol 137: 210-213.

21. Akman L, Rio RV, Beard CB, Aksoy S (2001) Genome size determination and coding capacity of Sodalis glossinidius, an enteric symbiont of tsetse flies, as revealed by hybridization to Escherichia coli gene arrays. J Bacteriol 183: 4517-4525.

22. Aksoy S, Caccone A, Galvani AP, Okedi LM Glossina fuscipes populations provide insights for human African trypanosomiasis transmission in Uganda. Trends Parasitol 2013 Aug;29(8):394-406 doi: 101016/jpt201306005 Epub 2013 Jul 8.

23. Aksoy S, Rio RV (2005) Interactions among multiple genomes: tsetse, its symbionts and trypanosomes. Insect Biochem Mol Biol 35: 691-698.

24. Alam U, Hyseni C, Symula RE, Brelsfoard C, Wu Y, et al. Implications of microfauna-host interactions for trypanosome transmission dynamics in Glossina fuscipes fuscipes in Uganda. Appl Environ Microbiol 2012 Jul;78(13):4627-37 doi: 101128/AEM00806-12 Epub 2012 Apr 27.

25. Alam U, Medlock J, Brelsfoard C, Pais R, Lohs C, et al. Wolbachia symbiont infections induce strong cytoplasmic incompatibility in the tsetse fly Glossina morsitans. PLoS Pathog 2011 Dec;7(12):e1002415 doi: 101371/journalppat1002415 Epub 2011 Dec 8.

26. Alatortsev VS, Cruz-Reyes J, Zhelonkina AG, Sollner-Webb B (2008) Trypanosoma brucei RNA editing: coupled cycles of U deletion reveal processive activity of the editing complex. Mol Cell Biol 28: 2437-2445.

27. Albajar-Vinas P, Jannin J The hidden Chagas disease burden in Europe. Euro Surveill 16.

28. Albrecht H (1997) Redefining AIDS: towards a modification of the current AIDS case definition. Clin Infect Dis 24: 64-74.

29. Alcover MM, Gramiccia M, Di Muccio T, Ballart C, Castillejo S, et al. Application of molecular techniques in the study of natural infection of Leishmania infantum vectors and utility of sandfly blood meal digestion for epidemiological surveys of leishmaniasis. Parasitol Res 2012 Aug;111(2):515-23 doi: 101007/s00436-012-2863-4 Epub 2012 Mar 2.

30. Alter HJ (2008) Pathogen reduction: a precautionary principle paradigm. Transfus Med Rev 22: 97-102.

31. Alvar J, Canavate C, Gutierrez-Solar B, Jimenez M, Laguna F, et al. (1997) Leishmania and human immunodeficiency virus coinfection: the first 10 years. Clin Microbiol Rev 10: 298-319.

32. Alvarez MG, Postan M, Weatherly DB, Albareda MC, Sidney J, et al. (2008) HLA Class I-T cell epitopes from trans-sialidase proteins reveal functionally distinct subsets of CD8+ T cells in chronic Chagas disease. PLoS Negl Trop Dis 2: e288.

33. Ambroise-Thomas P (2009) [Chagas disease]. Bull Soc Pathol Exot 102: 274.

34. Anderson KJ, Kuhn RE (1989) Elevated environmental temperature enhances immunity in experimental Chagas' disease. Infect Immun 57: 13-17.

35. Anderson LW, Banks KL (1982) Early course of infection in susceptible and resistant strains of mice, using [3H]uridine-labeled Trypanosoma brucei subsp. brucei. Infect Immun 36: 525-530.

36. Anderson NE, Mubanga J, Fevre EM, Picozzi K, Eisler MC, et al. Characterisation of the wildlife reservoir community for human and animal trypanosomiasis in the Luangwa Valley, Zambia. PLoS Negl Trop Dis 5: e1211.

37. Anderson TJ (1990) Blood parasites of mammals from Papua New Guinea. J Wildl Dis 26: 291-294.

38. Andersson B The Trypanosoma cruzi genome; conserved core genes and extremely variable surface molecule families. Res Microbiol 162: 619-625.

39. Andersson N, Morales A, Nava E, Martinez E, Rodriguez I, et al. (1990) Trypanosoma cruzi infection in the Mexican state of Guerrero: a seroepidemiological (ELISA) survey of 20 communities. J Trop Med Hyg 93: 341-346.

40. Andrade MC, Dick EJ, Jr., Guardado-Mendoza R, Hohmann ML, Mejido DC, et al. (2009) Nonspecific lymphocytic myocarditis in baboons is associated with Trypanosoma cruzi infection. Am J Trop Med Hyg 81: 235-239.

41. Apanius V, Kirkpatrick CE (1988) Preliminary report of Haemoproteus tinnunculi infection in a breeding population of American kestrels (Falco sparverius). J Wildl Dis 24: 150-153.

42. Appleman MD, Shulman IA, Saxena S, Kirchhoff LV (1993) Use of a questionnaire to identify potential blood donors at risk for infection with Trypanosoma cruzi. Transfusion 33: 61-64.

43. Arafa MS, Thaver S (1974) A seroepidemiological survey of Trypanosoma and Toxoplasma in Rattus norvegicus in England. J Egypt Public Health Assoc 49: 231-243.

44. Aragort W, Alvarez MF, Leiro JL, Sanmartin ML (2005) Blood protozoans in elasmobranchs of the family Rajidae from Galicia (NW Spain). Dis Aquat Organ 65: 63-68.

45. Araujo A, Hall WW (2004) Human T-lymphotropic virus type II and neurological disease. Ann Neurol 56: 10-19.

46. Araujo CA, Waniek PJ, Jansen AM (2009) An overview of Chagas disease and the role of triatomines on its distribution in Brazil. Vector Borne Zoonotic Dis 9: 227-234.

47. Arhin GK, Li H, Ullu E, Tschudi C (2006) A protein related to the vaccinia virus cap-specific methyltransferase VP39 is involved in cap 4 modification in Trypanosoma brucei. RNA 12: 53-62.

48. Arhin GK, Shen S, Irmer H, Ullu E, Tschudi C (2004) Role of a 300-kilodalton nuclear complex in the maturation of Trypanosoma brucei initiator methionyl-tRNA. Eukaryot Cell 3: 893-899.

49. Armstrong AC, Gidding S, Gjesdal O, Wu C, Bluemke DA, et al. LV mass assessed by echocardiography and CMR, cardiovascular outcomes, and medical practice. JACC Cardiovasc Imaging 2012 Aug;5(8):837-48 doi: 101016/jjcmg201206003.

50. Arnett FC, Reveille JD, Moutsopoulos HM, Georgescu L, Elkon KB (1996) Ribosomal P autoantibodies in systemic lupus erythematosus. Frequencies in different ethnic groups and clinical and immunogenetic associations. Arthritis Rheum 39: 1833-1839.

51. Arrick BA, Griffith OW, Cerami A (1981) Inhibition of glutathione synthesis as a chemotherapeutic strategy for trypanosomiasis. J Exp Med 153: 720-725.

52. Artzrouni M, Gouteux JP (2001) A model of Gambian sleeping sickness with open vector populations. IMA J Math Appl Med Biol 18: 99-117.

53. Assumpcao TC, Eaton DP, Pham VM, Francischetti IM, Aoki V, et al. An insight into the sialotranscriptome of Triatoma matogrossensis, a kissing bug associated with fogo selvagem in South America. Am J Trop Med Hyg 2012 Jun;86(6):1005-14 doi: 104269/ajtmh201211-0690.

54. Atouguia J, Costa J (1999) Therapy of human African trypanosomiasis: current situation. Mem Inst Oswaldo Cruz 94: 221-224.

55. Attardo GM, Ribeiro JM, Wu Y, Berriman M, Aksoy S Transcriptome analysis of reproductive tissue and intrauterine developmental stages of the tsetse fly (Glossina morsitans morsitans). BMC Genomics 11: 160.

56. Attardo GM, Strickler-Dinglasan P, Perkin SA, Caler E, Bonaldo MF, et al. (2006) Analysis of fat body transcriptome from the adult tsetse fly, Glossina morsitans morsitans. Insect Mol Biol 15: 411-424.

57. Auty HK, Picozzi K, Malele I, Torr SJ, Cleaveland S, et al. Using molecular data for epidemiological inference: assessing the prevalence of Trypanosoma brucei rhodesiense in tsetse in Serengeti, Tanzania. PLoS Negl Trop Dis 2012 Jan;6(1):e1501 doi: 101371/journalpntd0001501 Epub 2012 Jan 31.

58. Ayed Z, Brindel I, Bouteille B, Van Meirvenne N, Doua F, et al. (1997) Detection and characterization of autoantibodies directed against neurofilament proteins in human African trypanosomiasis. Am J Trop Med Hyg 57: 1-6.

59. Baer B, Schmid-Hempel P (2001) Unexpected consequences of polyandry for parasitism and fitness in the bumblebee, Bombus terrestris. Evolution 55: 1639-1643.

60. Bafort JM, Molyneux D, Racey PA (1970) The sub-genus Schizotrypanum in Britain. Trans R Soc Trop Med Hyg 64: 472.

61. Bajer A, Pawelczyk A, Behnke JM, Gilbert FS, Sinski E (2001) Factors affecting the component community structure of haemoparasites in bank voles (Clethrionomys glareolus) from the Mazury Lake District region of Poland. Parasitology 122 Pt 1: 43-54.

62. Baker DW, Wright RF (1994) Management of heart failure. IV. Anticoagulation for patients with heart failure due to left ventricular systolic dysfunction. JAMA 272: 1614-1618.

63. Baker RD (1989) Calculating the basic reproductive rate Ro when there are 2 or more pathogens. Ann Soc Belg Med Trop 69 Suppl 1: 99-107; discussion 144.

64. Baker RD, Maudlin I, Milligan PJ, Molyneux DH, Welburn SC (1990) The possible role of Rickettsia-like organisms in trypanosomiasis epidemiology. Parasitology 100 Pt 2: 209-217.

65. Balana-Fouce R, Redondo CM, Perez-Pertejo Y, Diaz-Gonzalez R, Reguera RM (2006) Targeting atypical trypanosomatid DNA topoisomerase I. Drug Discov Today 11: 733-740.

66. Balmer O, Beadell JS, Gibson W, Caccone A Phylogeography and taxonomy of Trypanosoma brucei. PLoS Negl Trop Dis 5: e961.

67. Balmer O, Caccone A (2008) Multiple-strain infections of Trypanosoma brucei across Africa. Acta Trop 107: 275-279.

68. Banuls AL, Hide M, Tibayrenc M (1999) Molecular epidemiology and evolutionary genetics of Leischmania parasites. Int J Parasitol 29: 1137-1147.

69. Baptista Rosas RC, Riquelme M (2007) [The epidemiology of coccidioidomycosis in Mexico]. Rev Iberoam Micol 24: 100-105.

70. Barbu CM, Hong A, Manne JM, Small DS, Quintanilla Calderon JE, et al. The effects of city streets on an urban disease vector. PLoS Comput Biol 2013;9(1):e1002801 doi: 101371/journalpcbi1002801 Epub 2013 Jan 17.

71. Bargues MD, Marcilla A, Dujardin JP, Mas-Coma S (2002) Triatomine vectors of Trypanosoma cruzi: a molecular perspective based on nuclear ribosomal DNA markers. Trans R Soc Trop Med Hyg 96 Suppl 1: S159-164.

72. Bargues MD, Zuriaga MA, Mas-Coma S Nuclear rDNA pseudogenes in Chagas disease vectors: Evolutionary implications of a new 5.8S+ITS-2 paralogous sequence marker in triatomines of North, Central and northern South America. Infect Genet Evol 2013 Nov 14;21C:134-156 doi: 101016/jmeegid201310028.

73. Barker RH, Jr., Liu H, Hirth B, Celatka CA, Fitzpatrick R, et al. (2009) Novel S-adenosylmethionine decarboxylase inhibitors for the treatment of human African trypanosomiasis. Antimicrob Agents Chemother 53: 2052-2058.

74. Barnabe C, Brisse S, Tibayrenc M (2000) Population structure and genetic typing of Trypanosoma cruzi, the agent of Chagas disease: a multilocus enzyme electrophoresis approach. Parasitology 120 ( Pt 5): 513-526.

75. Barnabe C, Neubauer K, Solari A, Tibayrenc M (2001) Trypanosoma cruzi: presence of the two major phylogenetic lineages and of several lesser discrete typing units (DTUs) in Chile and Paraguay. Acta Trop 78: 127-137.

76. Barnabe C, Yaeger R, Pung O, Tibayrenc M (2001) Trypanosoma cruzi: a considerable phylogenetic divergence indicates that the agent of Chagas disease is indigenous to the native fauna of the United States. Exp Parasitol 99: 73-79.

77. Barnard WH, Bair RD (1986) Prevalence of avian hematozoa in central Vermont. J Wildl Dis 22: 365-374.

78. Baroldi G, Silver MD, De Maria R, Parolini M, Turillazzi E, et al. (2003) Frequency and extent of contraction band necrosis in orthotopically transplanted human hearts. A morphometric study. Int J Cardiol 88: 267-278.

79. Barr SC, Brown CC, Dennis VA, Klei TR (1991) The lesions and prevalence of Trypanosoma cruzi in opossums and armadillos from southern Louisiana. J Parasitol 77: 624-627.

80. Barr SC, Dennis VA, Klei TR (1991) Serologic and blood culture survey of Trypanosoma cruzi infection in four canine populations of southern Louisiana. Am J Vet Res 52: 570-573.

81. Barr SC, Van Beek O, Carlisle-Nowak MS, Lopez JW, Kirchhoff LV, et al. (1995) Trypanosoma cruzi infection in Walker hounds from Virginia. Am J Vet Res 56: 1037-1044.

82. Barrett MP, Croft SL Management of trypanosomiasis and leishmaniasis. Br Med Bull 2012;104:175-96 doi: 101093/bmb/lds031 Epub 2012 Nov 7.

83. Barrett MP, Vincent IM, Burchmore RJ, Kazibwe AJ, Matovu E Drug resistance in human African trypanosomiasis. Future Microbiol 6: 1037-1047.

84. Barry MA, Bezek S, Serpa JA, Hotez PJ, Woc-Colburn L Neglected infections of poverty in Texas and the rest of the United States: management and treatment options. Clin Pharmacol Ther 2012 Aug;92(2):170-81 doi: 101038/clpt201285 Epub 2012 Jul 4.

85. Barry MA, Weatherhead JE, Hotez PJ, Woc-Colburn L Childhood parasitic infections endemic to the United States. Pediatr Clin North Am 2013 Apr;60(2):471-85 doi: 101016/jpcl201212011 Epub 2013 Jan 18.

86. Barta JR, Desser SS (1984) Blood parasites of amphibians from Algonquin Park, Ontario. J Wildl Dis 20: 180-189.

87. Batchelor NA, Atkinson PM, Gething PW, Picozzi K, Fevre EM, et al. (2009) Spatial predictions of Rhodesian Human African Trypanosomiasis (sleeping sickness) prevalence in Kaberamaido and Dokolo, two newly affected districts of Uganda. PLoS Negl Trop Dis 3: e563.

88. Baudon D, Boutin JP, Louis FJ, Drevet D (1999) [Major African endemic diseases at the dawn of the year 2000]. Med Trop (Mars) 59: 5-13.

89. Bauer B, Holzgrefe B, Mahama CI, Baumann MP, Mehlitz D, et al. Managing tsetse transmitted trypanosomosis by insecticide treated nets--an affordable and sustainable method for resource poor pig farmers in Ghana. PLoS Negl Trop Dis 5: e1343.

90. Bauer B, Kabore I, Liebisch A, Meyer F, Petrich-Bauer J (1992) Simultaneous control of ticks and tsetse flies in Satiri, Burkina Faso, by the use of flumethrin pour on for cattle. Trop Med Parasitol 43: 41-46.

91. Baumann MP, Zessin KH (1992) Productivity and health of camels (Camelus dromedarius) in Somalia: associations with trypanosomosis and brucellosis. Trop Anim Health Prod 24: 145-156.

92. Beard CB, Butler JF, Hall DW (1990) Prevalence and biology of endosymbionts of fleas (Siphonaptera: Pulicidae) from dogs and cats in Alachua County, Florida. J Med Entomol 27: 1050-1061.

93. Becker JL (2003) Vector-borne illnesses and the safety of the blood supply. Curr Hematol Rep 2: 511-517.

94. Behbehani K (1998) Candidate parasitic diseases. Bull World Health Organ 76 Suppl 2: 64-67.

95. Benchimol Barbosa PR (2006) The oral transmission of Chagas' disease: an acute form of infection responsible for regional outbreaks. Int J Cardiol 112: 132-133.

96. Bengtsson E (1967) [Control of blood donors. 4. Tropical diseases and blood transfusion]. Lakartidningen 64: 1973-1976.

97. Bennett GF, Aguirre AA, Cook RS (1991) Blood parasites of some birds from northeastern Mexico. J Parasitol 77: 38-41.

98. Bennett GF, Earle RA, Du Toit H, Huchzermeyer FW (1992) A host-parasite catalogue of the haematozoa of the sub-Saharan birds. Onderstepoort J Vet Res 59: 1-73.

99. Bennett GF, Garvin M, Bates JM (1991) Avian hematozoa from west-central Bolivia. J Parasitol 77: 207-211.

100. Bennett GF, Laird M (1973) Collaborative investigations into avian malarias: an international research programme. J Wildl Dis 9: 26-28.

101. Berche P (2001) [Jamot and the myth of Sisyphus]. Rev Prat 51: 1401-1404.

102. Bergquist NR (2001) Vector-borne parasitic diseases: new trends in data collection and risk assessment. Acta Trop 79: 13-20.

103. Berlin D, Nasereddin A, Azmi K, Ereqat S, Abdeen Z, et al. Longitudinal study of an outbreak of Trypanosoma evansi infection in equids and dromedary camels in Israel. Vet Parasitol 174: 317-322.

104. Berlin D, Nasereddin A, Azmi K, Ereqat S, Abdeen Z, et al. Prevalence of Trypanosoma evansi in horses in Israel evaluated by serology and reverse dot blot. Res Vet Sci 2012 Dec;93(3):1225-30 doi: 101016/jrvsc201204009 Epub 2012 May 11.

105. Berneman A, Montout L, Goyard S, Chamond N, Cosson A, et al. Combined approaches for drug design points the way to novel proline racemase inhibitor candidates to fight Chagas' disease. PLoS One 2013 Apr 16;8(4):e60955 doi: 101371/journalpone0060955 Print 2013.

106. Berrang-Ford L, Berke O, Abdelrahman L, Waltner-Toews D, McDermott J (2006) Spatial analysis of sleeping sickness, southeastern Uganda, 1970-2003. Emerg Infect Dis 12: 813-820.

107. Berrang-Ford L, Berke O, Sweeney S, Abdelrahman L Sleeping sickness in southeastern Uganda: a spatio-temporal analysis of disease risk, 1970-2003. Vector Borne Zoonotic Dis 10: 977-988.

108. Berrang-Ford L, Lundine J, Breau S Conflict and human African trypanosomiasis. Soc Sci Med 72: 398-407.

109. Berrang-Ford L, Odiit M, Maiso F, Waltner-Toews D, McDermott J (2006) Sleeping sickness in Uganda: revisiting current and historical distributions. Afr Health Sci 6: 223-231.

110. Berrizbeitia M, Ndao M, Bubis J, Gottschalk M, Ache A, et al. (2006) Field evaluation of four novel enzyme immunoassays for Chagas' disease in Venezuela blood banks: comparison of assays using fixed-epimastigotes, fixed-trypomastigotes or trypomastigote excreted-secreted antigens from two Trypanosoma cruzi strains. Transfus Med 16: 419-431.

111. Bethony JM, Cole RN, Guo X, Kamhawi S, Lightowlers MW, et al. Vaccines to combat the neglected tropical diseases. Immunol Rev 239: 237-270.

112. Bienek DR, Plouffe DA, Wiegertjes GF, Belosevic M (2002) Immunization of goldfish with excretory/secretory molecules of Trypanosoma danilewskyi confers protection against infection. Dev Comp Immunol 26: 649-657.

113. Bilodeau M, Burns S, Gawoski J, Moschella S, Ooi W Co-morbid infections in Hansen's disease patients in the United States: considerations for treatment. Am J Trop Med Hyg 2013 Oct;89(4):781-3 doi: 104269/ajtmh13-0167 Epub 2013 Aug 26.

114. Bisoffi Z, Beltrame A, Monteiro G, Arzese A, Marocco S, et al. (2005) African trypanosomiasis gambiense, Italy. Emerg Infect Dis 11: 1745-1747.

115. Bisser S, Courtioux B [Sleeping sickness: end of the epidemic outbreak?]. Rev Neurol (Paris) 2012 Mar;168(3):230-8 doi: 101016/jneurol201112004 Epub 2012 Mar 6.

116. Bittencourt AL, Vieira GO, Tavares HC, Mota E, Maguire J (1984) Esophageal involvement in congenital Chagas' disease. Report of a case with megaesophagus. Am J Trop Med Hyg 33: 30-33.

117. Black CM, Israelski DM, Suzuki Y, Remington JS (1989) Effect of recombinant tumour necrosis factor on acute infection in mice with Toxoplasma gondii or Trypanosoma cruzi. Immunology 68: 570-574.

118. Blanchot I, Dabadie A, Tell G, Guiguen C, Faugere B, et al. (1992) [Recurrent fever episodes in an African child: diagnostic difficulties of trypanosomiasis in France]. Pediatrie 47: 179-183.

119. Blanchy S (2009) [Summary of Workshop. No1. Risk populations of Chagas disease in metropolitan France and congenital Chagas disease]. Bull Soc Pathol Exot 102: 330-332.

120. Blanco G, Rodriguez-Estrella R, Merino S, Bertellotti M (2001) Effects of spatial and host variables on hematozoa in white-crowned sparrows wintering in Baja California. J Wildl Dis 37: 786-790.

121. Blum J, Nkunku S, Burri C (2001) Clinical description of encephalopathic syndromes and risk factors for their occurrence and outcome during melarsoprol treatment of human African trypanosomiasis. Trop Med Int Health 6: 390-400.

122. Blum J, Schmid C, Burri C (2006) Clinical aspects of 2541 patients with second stage human African trypanosomiasis. Acta Trop 97: 55-64.

123. Blum JA, Neumayr AL, Hatz CF Human African trypanosomiasis in endemic populations and travellers. Eur J Clin Microbiol Infect Dis 31: 905-913.

124. Boccara F, Blanchard-Lemoine B, Sarda L, Bardet J, Le Guludec D, et al. (1998) [Diagnostic strategy in acute myocarditis]. Arch Mal Coeur Vaiss 91: 1151-1158.

125. Bohning D, Greiner M (1998) Prevalence estimation under heterogeneity in the example of bovine trypanosomosis in Uganda. Prev Vet Med 36: 11-23.

126. Boin ID, Boteon YL, Stucchi RS, Pereira MI, Portugal TC, et al. Serological profile of pretransplantation liver patients. Transplant Proc 42: 491-493.

127. Boisseau-Garsaud AM, Cales-Quist D, Desbois N, Jouannelle J, Jouannelle A, et al. (2000) A new case of cutaneous infection by a presumed monoxenous trypanosomatid in the island of Martinique (French West Indies). Trans R Soc Trop Med Hyg 94: 51-52.

128. Bolognesi ML Multi-target-directed ligands as innovative tools to combat trypanosomatid diseases. Curr Top Med Chem 11: 2824-2833.

129. Booth CE, Elliott PF (2002) Hematological responses to hematozoa in North American and neotropical songbirds. Comp Biochem Physiol A Mol Integr Physiol 133: 451-467.

130. Bosseno MF, Barnabe C, Magallon Gastelum E, Lozano Kasten F, Ramsey J, et al. (2002) Predominance of Trypanosoma cruzi lineage I in Mexico. J Clin Microbiol 40: 627-632.

131. Bosseno MF, Garcia LS, Baunaure F, Gastelum EM, Gutierrez MS, et al. (2006) Identification in triatomine vectors of feeding sources and Trypanosoma cruzi variants by heteroduplex assay and a multiplex miniexon polymerase chain reaction. Am J Trop Med Hyg 74: 303-305.

132. Bourgeade A, Nosny Y, Faugere B, Pene P (1985) [African trypanosomiasis of icterohemorrhagic form]. Bull Soc Pathol Exot Filiales 78: 908-913.

133. Bouteille B, Chauviere G (1999) [Use of megazol for the treatment of trypanosomiasis]. Med Trop (Mars) 59: 321-330.

134. Bouteille B, Dumas M (1999) [Human African trypanosomiasis: reviving the challenge for a re-emerging illness]. Med Trop (Mars) 59: 20-24.

135. Bouyer J, Guerrini L, Desquesnes M, de la Rocque S, Cuisance D (2006) Mapping African Animal Trypanosomosis risk from the sky. Vet Res 37: 633-645.

136. Bouyer J, Pruvot M, Bengaly Z, Guerin PM, Lancelot R (2007) Learning influences host choice in tsetse. Biol Lett 3: 113-116.

137. Bouyer J, Stachurski F, Gouro AS, Lancelot R (2009) Control of bovine trypanosomosis by restricted application of insecticides to cattle using footbaths. Vet Parasitol 161: 187-193.

138. Bouyer J, Stachurski F, Kabore I, Bauer B, Lancelot R (2007) Tsetse control in cattle from pyrethroid footbaths. Prev Vet Med 78: 223-238.

139. Bower SM, Woo PT (1979) The prevalence of Trypanosoma catostomi in white sucker (Catostomus commersoni) from southern Ontario. J Wildl Dis 15: 429-431.

140. Bowling J, Walter EA (2009) Recognizing and meeting the challenge of Chagas disease in the USA. Expert Rev Anti Infect Ther 7: 1223-1234.

141. Bowman NM, Kawai V, Gilman RH, Bocangel C, Galdos-Cardenas G, et al. Autonomic dysfunction and risk factors associated with Trypanosoma cruzi infection among children in Arequipa, Peru. Am J Trop Med Hyg 84: 85-90.

142. Braendli B, Dankwa E, Junghanss T (1990) [East African sleeping sickness (Trypanosoma rhodesiense infection) in 2 Swiss travelers to the tropics]. Schweiz Med Wochenschr 120: 1348-1352.

143. Brashear RJ, Winkler MA, Schur JD, Lee H, Burczak JD, et al. (1995) Detection of antibodies to Trypanosoma cruzi among blood donors in the southwestern and western United States. I. Evaluation of the sensitivity and specificity of an enzyme immunoassay for detecting antibodies to T. cruzi. Transfusion 35: 213-218.

144. Bray DP, Bown KJ, Stockley P, Hurst JL, Bennett M, et al. (2007) Haemoparasites of common shrews (Sorex araneus) in Northwest England. Parasitology 134: 819-826.

145. Brenchley R, Tariq H, McElhinney H, Szoor B, Huxley-Jones J, et al. (2007) The TriTryp phosphatome: analysis of the protein phosphatase catalytic domains. BMC Genomics 8: 434.

146. Breniere SF, Bosseno MF, Gastelum EM, Soto Gutierrez MM, de Jesus Kasten Monges M, et al. Community participation and domiciliary occurrence of infected Meccus longipennis in two Mexican villages in Jalisco state. Am J Trop Med Hyg 83: 382-387.

147. Breniere SF, Bosseno MF, Magallon-Gastelum E, Castillo Ruvalcaba EG, Gutierrez MS, et al. (2007) Peridomestic colonization of Triatoma longipennis (Hemiptera, Reduviidae) and Triatoma barberi (Hemiptera, Reduviidae) in a rural community with active transmission of Trypanosoma cruzi in jalisco state, Mexico. Acta Trop 101: 249-257.

148. Breniere SF, Bosseno MF, Noireau F, Yacsik N, Liegeard P, et al. (2002) Integrate study of a Bolivian population infected by Trypanosoma cruzi, the agent of Chagas disease. Mem Inst Oswaldo Cruz 97: 289-295.

149. Breniere SF, Bosseno MF, Telleria J, Carrasco R, Vargas F, et al. (1995) Field application of polymerase chain reaction diagnosis and strain typing of Trypanosoma cruzi in Bolivian triatomines. Am J Trop Med Hyg 53: 179-184.

150. Breniere SF, Braquemond P, Solari A, Agnese JF, Tibayrenc M (1991) An isoenzyme study of naturally occurring clones of Trypanosoma cruzi isolated from both sides of the West Andes highland. Trans R Soc Trop Med Hyg 85: 62-66.

151. Breniere SF, Carrasco R, Revollo S, Aparicio G, Desjeux P, et al. (1989) Chagas' disease in Bolivia: clinical and epidemiological features and zymodeme variability of Trypanosoma cruzi strains isolated from patients. Am J Trop Med Hyg 41: 521-529.

152. Briceno-Leon R (2009) [Chagas disease in the Americas: an ecohealth perspective]. Cad Saude Publica 25 Suppl 1: S71-82.

153. Brisseau JM, Cebron JP, Petit T, Marjolet M, Cuilliere P, et al. (1988) Chagas' myocarditis imported into France. Lancet 1: 1046.

154. Brown EL, Roellig DM, Gompper ME, Monello RJ, Wenning KM, et al. Seroprevalence of Trypanosoma cruzi among eleven potential reservoir species from six states across the southern United States. Vector Borne Zoonotic Dis 10: 757-763.

155. Brown K (2008) From Ubombo to Mkhuzi: disease, colonial science, and the control of Nagana (livestock trypanosomosis) in Zululand, South Africa, C. 1894-1953. J Hist Med Allied Sci 63: 285-322.

156. Brun R, Blum J, Chappuis F, Burri C Human African trypanosomiasis. Lancet 375: 148-159.

157. Brun R, Schumacher R, Schmid C, Kunz C, Burri C (2001) The phenomenon of treatment failures in Human African Trypanosomiasis. Trop Med Int Health 6: 906-914.

158. Bruno JM (1997) [Correspondence of the French Ministry of Cooperation relative to the editorial by J. Drucker and T. Ancelle (Med. Trop. 1996; 56: 345-346 and 346-348)]. Med Trop (Mars) 57: 102, 103.

159. Bruzzone R, Dubois-Dalcq M, Grau GE, Griffin DE, Kristensson K (2009) Infectious diseases of the nervous system and their impact in developing countries. PLoS Pathog 5: e1000199.

160. Bucheton B, MacLeod A, Jamonneau V Human host determinants influencing the outcome of Trypanosoma brucei gambiense infections. Parasite Immunol 33: 438-447.

161. Buckner FS, Wilson AJ, Van Voorhis WC (1999) Detection of live Trypanosoma cruzi in tissues of infected mice by using histochemical stain for beta-galactosidase. Infect Immun 67: 403-409.

162. Buitrago R, Waleckx E, Bosseno MF, Zoveda F, Vidaurre P, et al. First report of widespread wild populations of Triatoma infestans (Reduviidae, Triatominae) in the valleys of La Paz, Bolivia. Am J Trop Med Hyg 82: 574-579.

163. Burreson EM (2007) Hemoflagellates of Oregon marine fishes with the description of new species of Trypanosoma and Trypanoplasma. J Parasitol 93: 1442-1451.

164. Burri C Chemotherapy against human African trypanosomiasis: is there a road to success? Parasitology 137: 1987-1994.

165. Burri C, Keiser J (2001) Pharmacokinetic investigations in patients from northern Angola refractory to melarsoprol treatment. Trop Med Int Health 6: 412-420.

166. Busch MP, Kleinman SH, Nemo GJ (2003) Current and emerging infectious risks of blood transfusions. JAMA 289: 959-962.

167. Buttenheim AM, Paz-Soldan V, Barbu C, Skovira C, Quintanilla Calderon J, et al. Is participation contagious? Evidence from a household vector control campaign in urban Peru. J Epidemiol Community Health 2013 Sep 23 doi: 101136/jech-2013-202661.

168. Bystrov IV, Ni GV (2007) [Hematozoon cenoses of small rodents and insectivora in the Orenburg Region]. Med Parazitol (Mosk): 31-35.

169. Cahn P, Belloso WH, Murillo J, Prada-Trujillo G (2000) AIDS in Latin America. Infect Dis Clin North Am 14: 185-209.

170. Camargo EC, Bacheschi LA, Massaro AR (2005) Stroke in Latin America. Neuroimaging Clin N Am 15: 283-296, x.

171. Cameron MM, Milligan PJ, Llanos-Cuentas A, Davies CR (1995) An association between phlebotomine sandflies and aphids in the Peruvian Andes. Med Vet Entomol 9: 127-132.

172. Cano J, Descalzo MA, Ndong-Mabale N, Ndongo-Asumu P, Bobuakasi L, et al. (2007) Spatial and temporal variability of the Glossina palpalis palpalis population in the Mbini focus (Equatorial Guinea). Int J Health Geogr 6: 36.

173. Canyuk B, Medrano FJ, Wenck MA, Focia PJ, Eakin AE, et al. (2004) Interactions at the dimer interface influence the relative efficiencies for purine nucleotide synthesis and pyrophosphorolysis in a phosphoribosyltransferase. J Mol Biol 335: 905-921.

174. Caporale VP, Battelli G, Semproni G (1980) Epidemiology of dourine in the equine population of the Abruzzi Region. Zentralbl Veterinarmed B 27: 489-498.

175. Capps L, Abad B (2004) Chagas cardiomyopathy and serologic testing in a small rural hospital in Chiapas, Mexico. Rev Panam Salud Publica 15: 337-340.

176. Carabarin-Lima A, Gonzalez-Vazquez MC, Rodriguez-Morales O, Baylon-Pacheco L, Rosales-Encina JL, et al. Chagas disease (American trypanosomiasis) in Mexico: an update. Acta Trop 2013 Aug;127(2):126-35 doi: 101016/jactatropica201304007 Epub 2013 Apr 30.

177. Caradonna K, Pereiraperrin M (2009) Preferential brain homing following intranasal administration of Trypanosoma cruzi. Infect Immun 77: 1349-1356.

178. Cardinal MV, Castanera MB, Lauricella MA, Cecere MC, Ceballos LA, et al. (2006) A prospective study of the effects of sustained vector surveillance following community-wide insecticide application on Trypanosoma cruzi infection of dogs and cats in rural Northwestern Argentina. Am J Trop Med Hyg 75: 753-761.

179. Cardinal MV, Lauricella MA, Ceballos LA, Lanati L, Marcet PL, et al. (2008) Molecular epidemiology of domestic and sylvatic Trypanosoma cruzi infection in rural northwestern Argentina. Int J Parasitol 38: 1533-1543.

180. Cardinal MV, Lauricella MA, Marcet PL, Orozco MM, Kitron U, et al. (2007) Impact of community-based vector control on house infestation and Trypanosoma cruzi infection in Triatoma infestans, dogs and cats in the Argentine Chaco. Acta Trop 103: 201-211.

181. Carlson JS, Martinez-Gomez JE, Valkiunas G, Loiseau C, Bell DA, et al. Diversity and phylogenetic relationships of hemosporidian parasites in birds of Socorro Island, Mexico, and their role in the re-introduction of the Socorro dove (Zenaida graysoni). J Parasitol 2013 Apr;99(2):270-6 doi: 101645/GE-32061 Epub 2012 Oct 8.

182. Carod-Artal FJ American trypanosomiasis. Handb Clin Neurol 2013;114:103-23 doi: 101016/B978-0-444-53490-300007-8.

183. Carod-Artal FJ Policy implications of the changing epidemiology of Chagas disease and stroke. Stroke 2013 Aug;44(8):2356-60 doi: 101161/STROKEAHA113000738 Epub 2013 Jun 11.

184. Carod-Artal FJ Trypanosomiasis, cardiomyopathy and the risk of ischemic stroke. Expert Rev Cardiovasc Ther 8: 717-728.

185. Carod-Artal FJ (2009) [Globalization, stroke and Chagas disease on the hundredth anniversary of its discovery]. Neurologia 24: 431-432.

186. Carod-Artal FJ, Gascon J Chagas disease and stroke. Lancet Neurol 9: 533-542.

187. Carod-Artal FJ, Vargas AP, Falcao T Stroke in asymptomatic Trypanosoma cruzi-infected patients. Cerebrovasc Dis 31: 24-28.

188. Carranza JC, Valadares HM, D'Avila DA, Baptista RP, Moreno M, et al. (2009) Trypanosoma cruzi maxicircle heterogeneity in Chagas disease patients from Brazil. Int J Parasitol 39: 963-973.

189. Carrasco HJ, Frame IA, Valente SA, Miles MA (1996) Genetic exchange as a possible source of genomic diversity in sylvatic populations of Trypanosoma cruzi. Am J Trop Med Hyg 54: 418-424.

190. Castilho TM, Camargo LM, McMahon-Pratt D, Shaw JJ, Floeter-Winter LM (2008) A real-time polymerase chain reaction assay for the identification and quantification of American Leishmania species on the basis of glucose-6-phosphate dehydrogenase. Am J Trop Med Hyg 78: 122-132.

191. Castro E, Girones N, Bueno JL, Carrion J, Lin L, et al. (2007) The efficacy of photochemical treatment with amotosalen HCl and ultraviolet A (INTERCEPT) for inactivation of Trypanosoma cruzi in pooled buffy-coat platelets. Transfusion 47: 434-441.

192. Castro-Sesquen YE, Gilman RH, Yauri V, Cok J, Angulo N, et al. Detection of soluble antigen and DNA of Trypanosoma cruzi in urine is independent of renal injury in the guinea pig model. PLoS One 2013;8(3):e58480 doi: 101371/journalpone0058480 Epub 2013 Mar 8.

193. Cattand P, Jannin J, Lucas P (2001) Sleeping sickness surveillance: an essential step towards elimination. Trop Med Int Health 6: 348-361.

194. Cavazzana M, Jr., Marcili A, Lima L, da Silva FM, Junqueira AC, et al. Phylogeographical, ecological and biological patterns shown by nuclear (ssrRNA and gGAPDH) and mitochondrial (Cyt b) genes of trypanosomes of the subgenus Schizotrypanum parasitic in Brazilian bats. Int J Parasitol 40: 345-355.

195. Ceballos LA, Cardinal MV, Vazquez-Prokopec GM, Lauricella MA, Orozco MM, et al. (2006) Long-term reduction of Trypanosoma cruzi infection in sylvatic mammals following deforestation and sustained vector surveillance in northwestern Argentina. Acta Trop 98: 286-296.

196. Ceballos LA, Piccinali RV, Marcet PL, Vazquez-Prokopec GM, Cardinal MV, et al. Hidden sylvatic foci of the main vector of Chagas disease Triatoma infestans: threats to the vector elimination campaign? PLoS Negl Trop Dis 5: e1365.

197. Ceballos LA, Vazquez-Prokopec GM, Cecere MC, Marcet PL, Gurtler RE (2005) Feeding rates, nutritional status and flight dispersal potential of peridomestic populations of Triatomainfestans in rural northwestern Argentina. Acta Trop 95: 149-159.

198. Cecchi G, Courtin F, Paone M, Diarra A, Franco JR, et al. (2009) Mapping sleeping sickness in Western Africa in a context of demographic transition and climate change. Parasite 16: 99-106.

199. Cecchi G, Paone M, Franco JR, Fevre EM, Diarra A, et al. (2009) Towards the Atlas of human African trypanosomiasis. Int J Health Geogr 8: 15.

200. Cecere MC, Vasquez-Prokopec GM, Gurtler RE, Kitron U (2006) Reinfestation sources for Chagas disease vector, Triatoma infestans, Argentina. Emerg Infect Dis 12: 1096-1102.

201. Cecere MC, Vazquez-Prokopec GM, Gurtler RE, Kitron U (2004) Spatio-temporal analysis of reinfestation by Triatoma infestans (Hemiptera: Reduviidae) following insecticide spraying in a rural community in northwestern Argentina. Am J Trop Med Hyg 71: 803-810.

202. Cerny O, Votypka J, Svobodova M Spatial feeding preferences of ornithophilic mosquitoes, blackflies and biting midges. Med Vet Entomol 25: 104-108.

203. Chalvet-Monfray K, Artzrouni M, Gouteux JP, Auger P, Sabatier P (1998) A two-patch model of Gambian sleeping sickness: application to vector control strategies in a village and plantations. Acta Biotheor 46: 207-222.

204. Chamaille L, Tran A, Meunier A, Bourdoiseau G, Ready P, et al. Environmental risk mapping of canine leishmaniasis in France. Parasit Vectors 3: 31.

205. Chamond N, Coatnoan N, Minoprio P (2002) Immunotherapy of Trypanosoma cruzi infections. Curr Drug Targets Immune Endocr Metabol Disord 2: 247-254.

206. Chappuis F, Alirol E, d'Acremont V, Bottieau E, Yansouni CP Rapid diagnostic tests for non-malarial febrile illness in the tropics. Clin Microbiol Infect 2013 May;19(5):422-31 doi: 101111/1469-069112154 Epub 2013 Feb 15.

207. Chappuis F, Stivanello E, Adams K, Kidane S, Pittet A, et al. (2004) Card agglutination test for trypanosomiasis (CATT) end-dilution titer and cerebrospinal fluid cell count as predictors of human African Trypanosomiasis (Trypanosoma brucei gambiense) among serologically suspected individuals in southern Sudan. Am J Trop Med Hyg 71: 313-317.

208. Chappuis F, Udayraj N, Stietenroth K, Meussen A, Bovier PA (2005) Eflornithine is safer than melarsoprol for the treatment of second-stage Trypanosoma brucei gambiense human African trypanosomiasis. Clin Infect Dis 41: 748-751.

209. Charles RA, Kjos S, Ellis AE, Barnes JC, Yabsley MJ Southern plains woodrats (Neotoma micropus) from southern Texas are important reservoirs of two genotypes of Trypanosoma cruzi and host of a putative novel Trypanosoma species. Vector Borne Zoonotic Dis 2013 Jan;13(1):22-30 doi: 101089/vbz20110817 Epub 2012 Nov 5.

210. Checchi F, Chappuis F, Karunakara U, Priotto G, Chandramohan D Accuracy of five algorithms to diagnose gambiense human African trypanosomiasis. PLoS Negl Trop Dis 5: e1233.

211. Checchi F, Cox AP, Chappuis F, Priotto G, Chandramohan D, et al. Prevalence and under-detection of gambiense human African trypanosomiasis during mass screening sessions in Uganda and Sudan. Parasit Vectors 2012 Aug 7;5:157 doi: 101186/1756-3305-5-157.

212. Checchi F, Filipe JA, Barrett MP, Chandramohan D (2008) The natural progression of Gambiense sleeping sickness: what is the evidence? PLoS Negl Trop Dis 2: e303.

213. Cheke RA, Hassall M, Peirce MA (1976) Blood parasites of British birds and notes on their seasonal occurrence at two rural sites in England. J Wildl Dis 12: 133-138.

214. Chen X, Li S, Aksoy S (1999) Concordant evolution of a symbiont with its host insect species: molecular phylogeny of genus Glossina and its bacteriome-associated endosymbiont, Wigglesworthia glossinidia. J Mol Evol 48: 49-58.

215. Cheng Q, Aksoy S (1999) Tissue tropism, transmission and expression of foreign genes in vivo in midgut symbionts of tsetse flies. Insect Mol Biol 8: 125-132.

216. Chevrier C, Canini F, Darsaud A, Cespuglio R, Buguet A, et al. (2005) Clinical assessment of the entry into neurological state in rat experimental African trypanosomiasis. Acta Trop 95: 33-39.

217. Chianella S, Semprevivo M, Peng ZC, Zaccheo D, Bentivoglio M, et al. (1999) Microglia activation in a model of sleep disorder: an immunohistochemical study in the rat brain during Trypanosoma brucei infection. Brain Res 832: 54-62.

218. Chindalore V, Neas B, Reichlin M (1998) The association between anti-ribosomal P antibodies and active nephritis in systemic lupus erythematosus. Clin Immunol Immunopathol 87: 292-296.

219. Chretien JP, Smoak BL (2005) African Trypanosomiasis: Changing Epidemiology and Consequences. Curr Infect Dis Rep 7: 54-60.

220. Ciesielski S, Seed JR, Estrada J, Wrenn E (1993) The seroprevalence of cysticercosis, malaria, and Trypanosoma cruzi among North Carolina migrant farmworkers. Public Health Rep 108: 736-741.

221. Cisarovsky G, Schmid-Hempel P Few colonies of the host Bombus terrestris disproportionately affect the genetic diversity of its parasite, Crithidia bombi. Infect Genet Evol 2013 Nov 18;21C:192-197 doi: 101016/jmeegid201311010.

222. Clark DV, Mammen MP, Jr., Nisalak A, Puthimethee V, Endy TP (2005) Economic impact of dengue fever/dengue hemorrhagic fever in Thailand at the family and population levels. Am J Trop Med Hyg 72: 786-791.

223. Clark GG (1972) Trypanosomes from mule deer in New Mexico and Colorado. J Wildl Dis 8: 325-326.

224. Clausen PH, Bauer B, Zessin KH, Diall O, Bocoum Z, et al. Preventing and containing trypanocide resistance in the cotton zone of West Africa. Transbound Emerg Dis 57: 28-32.

225. Clausen PH, Chuluun S, Sodnomdarjaa R, Greiner M, Noeckler K, et al. (2003) A field study to estimate the prevalence of Trypanosoma equiperdum in Mongolian horses. Vet Parasitol 115: 9-18.

226. Clausen PH, Wiemann A, Patzelt R, Kakaire D, Poetzsch C, et al. (1998) Use of a PCR assay for the specific and sensitive detection of Trypanosoma Spp. in naturally infected dairy cattle in peri-urban Kampala, Uganda. Ann N Y Acad Sci 849: 21-31.

227. Click Lambert R, Kolivras KN, Resler LM, Brewster CC, Paulson SL (2008) The potential for emergence of Chagas disease in the United States. Geospat Health 2: 227-239.

228. Conrad ME (1981) Diseases transmissible by blood transfusion: viral hepatitis and other infectious disorders. Semin Hematol 18: 122-146.

229. Cooper RG (2008) Care, husbandry and diseases of the African giant rat (Cricetomys gambianus). J S Afr Vet Assoc 79: 62-66.

230. Corash L (1998) Inactivation of viruses, bacteria, protozoa, and leukocytes in platelet concentrates. Vox Sang 74 Suppl 2: 173-176.

231. Corash L (2000) New technologies for the inactivation of infectious pathogens in cellular blood components and the development of platelet substitutes. Baillieres Best Pract Res Clin Haematol 13: 549-563.

232. Corash L (2001) Inactivation of infectious pathogens in labile blood components: meeting the challenge. Transfus Clin Biol 8: 138-145.

233. Cordes N, Huang WF, Strange JP, Cameron SA, Griswold TL, et al. Interspecific geographic distribution and variation of the pathogens Nosema bombi and Crithidia species in United States bumble bee populations. J Invertebr Pathol 2012 Feb;109(2):209-16 doi: 101016/jjip201111005 Epub 2011 Nov 18.

234. Cordon-Obras C, Berzosa P, Ndong-Mabale N, Bobuakasi L, Buatiche JN, et al. (2009) Trypanosoma brucei gambiense in domestic livestock of Kogo and Mbini foci (Equatorial Guinea). Trop Med Int Health 14: 535-541.

235. Cordon-Obras C, Garcia-Estebanez C, Ndong-Mabale N, Abaga S, Ndongo-Asumu P, et al. Screening of Trypanosoma brucei gambiense in domestic livestock and tsetse flies from an insular endemic focus (Luba, Equatorial Guinea). PLoS Negl Trop Dis 4: e704.

236. Corrales RM, Mora MC, Negrette OS, Diosque P, Lacunza D, et al. (2009) Congenital Chagas disease involves Trypanosoma cruzi sub-lineage IId in the northwestern province of Salta, Argentina. Infect Genet Evol 9: 278-282.

237. Cortes-Bergoderi M, Thomas RJ, Albuquerque FN, Batsis JA, Burdiat G, et al. Validity of cardiovascular risk prediction models in Latin America and among Hispanics in the United States of America: a systematic review. Rev Panam Salud Publica 2012 Aug;32(2):131-9.

238. Cottontail VM, Wellinghausen N, Kalko EK (2009) Habitat fragmentation and haemoparasites in the common fruit bat, Artibeus jamaicensis (Phyllostomidae) in a tropical lowland forest in Panama. Parasitology 136: 1133-1145.

239. Courtenay O, Kovacic V, Gomes PA, Garcez LM, Quinnell RJ (2009) A long-lasting topical deltamethrin treatment to protect dogs against visceral leishmaniasis. Med Vet Entomol 23: 245-256.

240. Courtin D, Jamonneau V, Mathieu JF, Koffi M, Milet J, et al. (2006) Comparison of cytokine plasma levels in human African trypanosomiasis. Trop Med Int Health 11: 647-653.

241. Courtin D, Milet J, Jamonneau V, Yeminanga CS, Kumeso VK, et al. (2007) Association between human African trypanosomiasis and the IL6 gene in a Congolese population. Infect Genet Evol 7: 60-68.

242. Courtin F, Dupont S, Zeze DG, Jamonneau V, Sane B, et al. (2005) [Human African trypanosomiasis: urban transmission in the focus of Bonon (Cote d'Ivoire)]. Trop Med Int Health 10: 340-346.

243. Courtioux B, Pervieux L, Vatunga G, Marin B, Josenando T, et al. (2009) Increased CXCL-13 levels in human African trypanosomiasis meningo-encephalitis. Trop Med Int Health 14: 529-534.

244. Croft SL, Vivas L, Brooker S (2003) Recent advances in research and control of malaria, leishmaniasis, trypanosomiasis and schistosomiasis. East Mediterr Health J 9: 518-533.

245. Cross GA (2001) African trypanosomes in the 21st century: what is their future in science and in health? Int J Parasitol 31: 427-433.

246. Custer B, Agapova M, Martinez RH The cost-effectiveness of pathogen reduction technology as assessed using a multiple risk reduction model. Transfusion 50: 2461-2473.

247. Dale C, Welburn SC, Maudlin I, Milligan PJ (1995) The kinetics of maturation of trypanosome infections in tsetse. Parasitology 111 ( Pt 2): 187-191.

248. D'Alessandro A, Barreto P, Saravia N, Barreto M (1984) Epidemiology of Trypanosoma cruzi in the oriental plains of Colombia. Am J Trop Med Hyg 33: 1084-1095.

249. Daly JJ, DeGiusti DL (1971) Trypanosoma catostomi n. sp. from the white sucker Catostomus c. commersoni (Lacepede). J Protozool 18: 414-417.

250. Damaso CR, Esposito JJ, Condit RC, Moussatche N (2000) An emergent poxvirus from humans and cattle in Rio de Janeiro State: Cantagalo virus may derive from Brazilian smallpox vaccine. Virology 277: 439-449.

251. D'Amico F, Gouteux JP, Le Gall F, Cuisance D (1996) Are stable flies (Diptera: Stomoxyinae) vectors of Trypanosoma vivax in the Central African Republic? Vet Res 27: 161-170.

252. D'Archivio S, Cosson A, Medina M, Lang T, Minoprio P, et al. Non-invasive in vivo study of the Trypanosoma vivax infectious process consolidates the brain commitment in late infections. PLoS Negl Trop Dis 2013;7(1):e1976 doi: 101371/journalpntd0001976 Epub 2013 Jan 3.

253. D'Archivio S, Medina M, Cosson A, Chamond N, Rotureau B, et al. Genetic engineering of Trypanosoma (Dutonella) vivax and in vitro differentiation under axenic conditions. PLoS Negl Trop Dis 2011 Dec;5(12):e1461 doi: 101371/journalpntd0001461 Epub 2011 Dec 27.

254. Dardonville C, Barrett MP, Brun R, Kaiser M, Tanious F, et al. (2006) DNA binding affinity of bisguanidine and bis(2-aminoimidazoline) derivatives with in vivo antitrypanosomal activity. J Med Chem 49: 3748-3752.

255. Daubert MA, Stergiopoulos K, Brown DL A caravanning cardiomyopathy. Am J Med 124: 824-826.

256. Daugschies A (2001) [Importation of parasites by tourism and animal trading]. Dtsch Tierarztl Wochenschr 108: 348-352.

257. Davis AK, Hopkins WA Widespread trypanosome infections in a population of eastern hellbenders (Cryptobranchus alleganiensis alleganiensis) in Virginia, USA. Parasitol Res 2013 Jan;112(1):453-6 doi: 101007/s00436-012-3076-6 Epub 2012 Aug 25.

258. Davis S, Aksoy S, Galvani A A global sensitivity analysis for African sleeping sickness. Parasitology 138: 516-526.

259. Dayo GK, Bengaly Z, Messad S, Bucheton B, Sidibe I, et al. Prevalence and incidence of bovine trypanosomosis in an agro-pastoral area of southwestern Burkina Faso. Res Vet Sci 88: 470-477.

260. de La Rocque S, Michel JF, Bouyer J, De Wispelaere G, Cuisance D (2005) Geographical Information Systems in parasitology: a review of potential applications using the example of animal trypanosomosis in West Africa. Parassitologia 47: 97-104.

261. De La Rocque S, Michel JF, Cuisance D (2001) [Different potentials of a geographic information system for studies in epidemiology: example of animal trypanosomiasis in the Sudan region]. Med Trop (Mars) 61: 365-371.

262. De La Rocque S, Michel JF, De Wispelaere G, Cuisance D (2001) [New tools for the study of animal trypanosomiasis in the Sudan: model-building of dangerous epidemiological passage by remote sensing geographic information systems]. Parasite 8: 171-195.

263. De La Rocque S, Michel V, Plazanet D, Pin R (2004) Remote sensing and epidemiology: examples of applications for two vector-borne diseases. Comp Immunol Microbiol Infect Dis 27: 331-341.

264. de la Rua N, Stevens L, Dorn PL High genetic diversity in a single population of Triatoma sanguisuga (LeConte, 1855) inferred from two mitochondrial markers: Cytochrome b and 16S ribosomal DNA. Infect Genet Evol 11: 671-677.

265. de Lana M, da Silveira Pinto A, Barnabe C, Quesney V, Noel S, et al. (1998) Trypanosoma cruzi: compared vectorial transmissibility of three major clonal genotypes by Triatoma infestans. Exp Parasitol 90: 20-25.

266. de Nishioka SA, Gyorkos TW, Joseph L, Collet JP, MacLean JD (2003) Tattooing and transfusion-transmitted diseases in Brazil: a hospital-based cross-sectional matched study. Eur J Epidemiol 18: 441-449.

267. de Souza SM, de Abreu Vieira PM, Roatt BM, Reis LE, da Silva Fonseca K, et al. Dogs infected with the blood trypomastigote form of Trypanosoma cruzi display an increase expression of cytokines and chemokines plus an intense cardiac parasitism during acute infection. Mol Immunol 2013 Dec 5;58(1):92-97 doi: 101016/jmolimm201311007.

268. de Thoisy B, Vogel I, Reynes JM, Pouliquen JF, Carme B, et al. (2001) Health evaluation of translocated free-ranging primates in French Guiana. Am J Primatol 54: 1-16.

269. Delgado S, Castillo Neyra R, Quispe Machaca VR, Ancca Juarez J, Chou Chu L, et al. A history of chagas disease transmission, control, and re-emergence in peri-rural La Joya, Peru. PLoS Negl Trop Dis 5: e970.

270. Deng X, Sabino EC, Cunha-Neto E, Ribeiro AL, Ianni B, et al. Genome Wide Association Study (GWAS) of Chagas Cardiomyopathy in Trypanosoma cruzi Seropositive Subjects. PLoS One 2013 Nov 20;8(11):e79629 doi: 101371/journalpone0079629.

271. Deribe K, Meribo K, Gebre T, Hailu A, Ali A, et al. The burden of neglected tropical diseases in Ethiopia, and opportunities for integrated control and elimination. Parasit Vectors 2012 Oct 24;5:240 doi: 101186/1756-3305-5-240.

272. Desjeux P (1999) Global control and Leishmania HIV co-infection. Clin Dermatol 17: 317-325.

273. Desowitz RS (2002) Visiting the virus veteran. Interview by Philip Siekman. Fortune 146: 44.

274. Desquesnes M, Bengaly Z, Millogo L, Meme Y, Sakande H (2001) The analysis of the cross-reactions occurring in antibody-ELISA for the detection of trypanosomes can improve identification of the parasite species involved. Ann Trop Med Parasitol 95: 141-155.

275. Desquesnes M, Biteau-Coroller F, Bouyer J, Dia ML, Foil L (2009) Development of a mathematical model for mechanical transmission of trypanosomes and other pathogens of cattle transmitted by tabanids. Int J Parasitol 39: 333-346.

276. Desquesnes M, Bossard G, Patrel D, Herder S, Patout O, et al. (2008) First outbreak of Trypanosoma evansi in camels in metropolitan France. Vet Rec 162: 750-752.

277. Desquesnes M, Bossard G, Thevenon S, Patrel D, Ravel S, et al. (2009) Development and application of an antibody-ELISA to follow up a Trypanosoma evansi outbreak in a dromedary camel herd in France. Vet Parasitol 162: 214-220.

278. Desquesnes M, Dargantes A, Lai DH, Lun ZR, Holzmuller P, et al. Trypanosoma evansi and surra: a review and perspectives on transmission, epidemiology and control, impact, and zoonotic aspects. Biomed Res Int 2013;2013:321237 doi: 101155/2013/321237 Epub 2013 Sep 18.

279. Desquesnes M, Dia ML (2003) Mechanical transmission of Trypanosoma congolense in cattle by the African tabanid Atylotus agrestis. Exp Parasitol 105: 226-231.

280. Desquesnes M, Dia ML (2003) Trypanosoma vivax: mechanical transmission in cattle by one of the most common African tabanids, Atylotus agrestis. Exp Parasitol 103: 35-43.

281. Desquesnes M, Kamyingkird K, Pruvot M, Kengradomkij C, Bossard G, et al. (2009) Antibody-ELISA for Trypanosoma evansi: application in a serological survey of dairy cattle, Thailand, and validation of a locally produced antigen. Prev Vet Med 90: 233-241.

282. Desser SS (2000) The blood parasites of the spiny pocket mouse Liomys salvini (Thomas, 1893) from Costa Rica. J Parasitol 86: 156-157.

283. Deviche P, Fokidis HB, Lerbour B, Greiner E Blood parasitaemia in a high latitude flexible breeder, the white-winged crossbill, Loxia leucoptera: contribution of seasonal relapse versus new inoculations. Parasitology 137: 261-273.

284. Deviche P, Greiner EC, Manteca X (2001) Interspecific variability of prevalence in blood parasites of adult passerine birds during the breeding season in Alaska. J Wildl Dis 37: 28-35.

285. Deviche P, McGraw K, Greiner EC (2005) Interspecific differences in hematozoan infection in Sonoran desert Aimophila sparrows. J Wildl Dis 41: 532-541.

286. Devillers H, Lobry JR, Menu F (2008) An agent-based model for predicting the prevalence of Trypanosoma cruzi I and II in their host and vector populations. J Theor Biol 255: 307-315.

287. DeVisser MH, Messina JP (2009) Optimum land cover products for use in a Glossina-morsitans habitat model of Kenya. Int J Health Geogr 8: 39.

288. Dhiman M, Estrada-Franco JG, Pando JM, Ramirez-Aguilar FJ, Spratt H, et al. (2009) Increased myeloperoxidase activity and protein nitration are indicators of inflammation in patients with Chagas' disease. Clin Vaccine Immunol 16: 660-666.

289. Diaz LA, Arteaga LA, Hilario-Vargas J, Valenzuela JG, Li N, et al. (2004) Anti-desmoglein-1 antibodies in onchocerciasis, leishmaniasis and Chagas disease suggest a possible etiological link to Fogo selvagem. J Invest Dermatol 123: 1045-1051.

290. Dimock KA, Davis CD, Kuhn RE (1991) Effect of elevated environmental temperature on the antibody response of mice to Trypanosoma cruzi during the acute phase of infection. Infect Immun 59: 4377-4382.

291. Docampo R (1990) Sensitivity of parasites to free radical damage by antiparasitic drugs. Chem Biol Interact 73: 1-27.

292. Docampo R (2001) Recent developments in the chemotherapy of Chagas disease. Curr Pharm Des 7: 1157-1164.

293. Docampo R, Moreno SN, Cruz FS (1988) Enhancement of the cytotoxicity of crystal violet against Trypanosoma cruzi in the blood by ascorbate. Mol Biochem Parasitol 27: 241-247.

294. Docampo R, Moreno SN, Gadelha FR, de Souza W, Cruz FS (1988) Prevention of Chagas' disease resulting from blood transfusion by treatment of blood: toxicity and mode of action of gentian violet. Biomed Environ Sci 1: 406-413.

295. Dodd R (2009) Managing the microbiological safety of blood for transfusion: a US perspective. Future Microbiol 4: 807-818.

296. Dodd RY (2004) Current safety of the blood supply in the United States. Int J Hematol 80: 301-305.

297. Doerr W (1967) [Inflammatory diseases of the myocardium]. Verh Dtsch Ges Pathol 51: 67-101.

298. Dorn PL, Calderon C, Melgar S, Moguel B, Solorzano E, et al. (2009) Two distinct Triatoma dimidiata (Latreille, 1811) taxa are found in sympatry in Guatemala and Mexico. PLoS Negl Trop Dis 3: e393.

299. Dorn PL, Daigle ME, Combe CL, Tate AH, Stevens L, et al. Low prevalence of Chagas parasite infection in a nonhuman primate colony in Louisiana. J Am Assoc Lab Anim Sci 2012 Jul;51(4):443-7.

300. Dorn PL, Melgar S, Rouzier V, Gutierrez A, Combe C, et al. (2003) The Chagas vector, Triatoma dimidiata (Hemiptera: Reduviidae), is panmictic within and among adjacent villages in Guatemala. J Med Entomol 40: 436-440.

301. Dreyfuss G, Gayral P, Dubost G, Nicolas JA, Craciunescu D (1988) [Antiparasitic activity of Cis-Pt (II) pentamidine in experimental trypanosomiasis of sheep caused by Trypanosoma brucei brucei]. Bull Soc Pathol Exot Filiales 81: 626-631.

302. Dreyfuss G, Loiseau P, Wolf JG, Bories C, Gayral P, et al. (1988) [A new series of antiparasitic organic arsenicals: the spiroarsoranes. Experimental trypanocidal activity]. Bull Soc Pathol Exot Filiales 81: 561-570.

303. Ducheyne E, Mweempwa C, De Pus C, Vernieuwe H, De Deken R, et al. (2009) The impact of habitat fragmentation on tsetse abundance on the plateau of eastern Zambia. Prev Vet Med 91: 11-18.

304. Duffy CW, Maclean L, Sweeney L, Cooper A, Turner CM, et al. Population Genetics of Trypanosoma brucei rhodesiense: Clonality and Diversity within and between Foci. PLoS Negl Trop Dis 2013 Nov 14;7(11):e2526 doi: 101371/journalpntd0002526.

305. Dujardin JP, Schofield CJ, Tibayrenc M (1998) Population structure of Andean Triatoma infestans: allozyme frequencies and their epidemiological relevance. Med Vet Entomol 12: 20-29.

306. Dumas M, Bouteille B (1997) [Current status of trypanosomiasis]. Med Trop (Mars) 57: 65-69.

307. Dumas M, Bouteille B (2002) [Human African trypanosomiasis: present and future treatment ]. Bull Soc Pathol Exot 95: 341-344.

308. Durden LA, McLean RG, Oliver JH, Jr., Ubico SR, James AM (1997) Ticks, Lyme disease spirochetes, trypanosomes, and antibody to encephalitis viruses in wild birds from coastal Georgia and South Carolina. J Parasitol 83: 1178-1182.

309. Duvallet G, de La Rocque S, Reifenberg JM, Solano P, Lefrancois T, et al. (1999) Review on the molecular tools for the understanding of the epidemiology of animal trypanosomosis in West Africa. Mem Inst Oswaldo Cruz 94: 245-248.

310. Eberhard M, D'Alessandro A (1982) Congenital Trypanosoma cruzi infection in a laboratory-born squirrel monkey, Saimiri sciureus. Am J Trop Med Hyg 31: 931-933.

311. Eisler MC, Magona JW, Revie CW Diagnosis of cattle diseases endemic to sub-Saharan Africa: evaluating a low cost decision support tool in use by veterinary personnel. PLoS One 2012;7(7):e40687 doi: 101371/journalpone0040687 Epub 2012 Jul 12.

312. Elies R, Ferrari I, Wallukat G, Lebesgue D, Chiale P, et al. (1996) Structural and functional analysis of the B cell epitopes recognized by anti-receptor autoantibodies in patients with Chagas' disease. J Immunol 157: 4203-4211.

313. el-Sayed NM, Harkins PC, Fox RO, Anderson K, Patton CL (1995) Crystallization and preliminary X-ray investigation of the recombinant Trypanosoma brucei rhodesiense calmodulin. Proteins 21: 354-357.

314. Enemark H, Seibaek MB, Kirchhoff LV, Jensen GB (2000) [Chronic Chagas disease--an echo from youth]. Ugeskr Laeger 162: 2567-2569.

315. Enriquez GF, Cardinal MV, Orozco MM, Lanati L, Schijman AG, et al. Discrete typing units of Trypanosoma cruzi identified in rural dogs and cats in the humid Argentinean Chaco. Parasitology 2013 Mar;140(3):303-8 doi: 101017/S003118201200159X Epub 2012 Oct 12.

316. Erdmann H, Rossnagel C, Bohme J, Iwakura Y, Jacobs T, et al. IL-17A promotes macrophage effector mechanisms against Trypanosoma cruzi by trapping parasites in the endolysosomal compartment. Immunobiology 2013 Jun;218(6):910-23 doi: 101016/jimbio201210005 Epub 2012 Oct 26.

317. Esch KJ, Petersen CA Transmission and epidemiology of zoonotic protozoal diseases of companion animals. Clin Microbiol Rev 2013 Jan;26(1):58-85 doi: 101128/CMR00067-12.

318. Escriba JM, Ponce E, Romero Ade D, Vinas PA, Marchiol A, et al. (2009) Treatment and seroconversion in a cohort of children suffering from recent chronic Chagas infection in Yoro, Honduras. Mem Inst Oswaldo Cruz 104: 986-991.

319. Estes ME (1989) Chagas' disease. Crit Care Nurse 9: 48-64.

320. Estevens J, Fidalgo P, Tendeiro T, Chagas C, Ferra A, et al. (1993) Anti-Helicobacter pylori antibodies prevalence and gastric adenocarcinoma in Portugal: report of a case-control study. Eur J Cancer Prev 2: 377-380.

321. Estrada-Franco JG, Bhatia V, Diaz-Albiter H, Ochoa-Garcia L, Barbabosa A, et al. (2006) Human Trypanosoma cruzi infection and seropositivity in dogs, Mexico. Emerg Infect Dis 12: 624-630.

322. Evans TG, Vasconcelos IA, Lima JW, Teixeira JM, McAullife IT, et al. (1990) Canine visceral leishmaniasis in northeast Brazil: assessment of serodiagnostic methods. Am J Trop Med Hyg 42: 118-123.

323. Ewing SA, Carnahan DL (1967) Occurrence of Trypanosoma theileri in bovine peripheral blood. J Am Vet Med Assoc 150: 1131-1132.

324. Faiman R, Abbasi I, Jaffe C, Motro Y, Nasereddin A, et al. A newly emerged cutaneous leishmaniasis focus in northern Israel and two new reservoir hosts of Leishmania major. PLoS Negl Trop Dis 2013;7(2):e2058 doi: 101371/journalpntd0002058 Epub 2013 Feb 21.

325. Farikou O, Njiokou F, Cuny G, Geiger A Microsatellite genotyping reveals diversity within populations of Sodalis glossinidius, the secondary symbiont of tsetse flies. Vet Microbiol 150: 207-210.

326. Farrar RG, Klei TR (1990) Prevalence of Trypanosoma theileri in Louisiana cattle. J Parasitol 76: 734-736.

327. Farrar WE, Jr., Gibbins SD, Whitfield ST (1972) Low prevalence of antibody to Trypanosoma cruzi in Georgia. Am J Trop Med Hyg 21: 404-406.

328. Feit A, El-Sherif N, Korostoff S (1983) Chagas' disease masquerading as coronary artery disease. Arch Intern Med 143: 144-145.

329. Fejfar Z (1968) Cardiomyopathies--an international problem. Cardiologia 52: 9-19.

330. Feliciangeli MD, Sanchez-Martin MJ, Suarez B, Marrero R, Torrellas A, et al. (2007) Risk factors for Trypanosoma cruzi human infection in Barinas State, Venezuela. Am J Trop Med Hyg 76: 915-921.

331. Ferreira RM, Saad MH, Silva MG, Fonseca Lde S (2002) Non-tuberculous mycobacteria I: one year clinical isolates identification in Tertiary Hospital Aids Reference Center, Rio de Janeiro, Brazil, in pre highly active antiretroviral therapy era. Mem Inst Oswaldo Cruz 97: 725-729.

332. Ferro C, Morrison AC, Torres M, Pardo R, Wilson ML, et al. (1995) Age structure, blood-feeding behavior, and Leishmania chagasi infection in Lutzomyia longipalpis (Diptera: Psychodidae) at an endemic focus of visceral leishmaniasis in Colombia. J Med Entomol 32: 618-629.

333. Fevre EM, Coleman PG, Welburn SC, Maudlin I (2004) Reanalyzing the 1900-1920 sleeping sickness epidemic in Uganda. Emerg Infect Dis 10: 567-573.

334. Fevre EM, Tilley A, Picozzi K, Fyfe J, Anderson I, et al. (2006) Central point sampling from cattle in livestock markets in areas of human sleeping sickness. Acta Trop 97: 229-232.

335. Fevre EM, Wissmann BV, Welburn SC, Lutumba P (2008) The burden of human African trypanosomiasis. PLoS Negl Trop Dis 2: e333.

336. Field V, Gautret P, Schlagenhauf P, Burchard GD, Caumes E, et al. Travel and migration associated infectious diseases morbidity in Europe, 2008. BMC Infect Dis 10: 330.

337. Fischer-Tenhagen C, Hamblin C, Quandt S, Frolich K (2000) Serosurvey for selected infectious disease agents in free-ranging black and white rhinoceros in Africa. J Wildl Dis 36: 316-323.

338. Fitzpatrick MA, Caicedo JC, Stosor V, Ison MG Expanded infectious diseases screening program for Hispanic transplant candidates. Transpl Infect Dis 12: 336-341.

339. Fitzpatrick S, Feliciangeli MD, Sanchez-Martin MJ, Monteiro FA, Miles MA (2008) Molecular genetics reveal that silvatic Rhodnius prolixus do colonise rural houses. PLoS Negl Trop Dis 2: e210.

340. Flores-Figueroa J, Okhuysen PC, von Sonnenburg F, DuPont HL, Libman MD, et al. Patterns of illness in travelers visiting Mexico and Central America: the GeoSentinel experience. Clin Infect Dis 53: 523-531.

341. Florez O, Martin J, Gonzalez CI Interleukin 4, interleukin 4 receptor-alpha and interleukin 10 gene polymorphisms in Chagas disease. Parasite Immunol 33: 506-511.

342. Florian Sanz F, Gomez Navarro C, Castrillo Garcia N, Pedrote Martinez A, Lage Galle E (2005) [Chagasic cardiomyopathy in Spain: a diagnosis to bear in mind]. An Med Interna 22: 538-540.

343. Fournier-Wirth C, Jaffrezic-Renault N, Coste J Detection of blood-transmissible agents: can screening be miniaturized? Transfusion 50: 2032-2045.

344. Fourrier A, Becquet R, Vernes A, Mouton Y, Afchain D, et al. (1976) [II. Tryopanosomiasis, onchercercosis, tropical diseases that travel]. Lille Med 21: 457-459.

345. Fraker PJ, Caruso R, Kierszenbaum F (1982) Alteration of the immune and nutritional status of mice by synergy between zinc deficiency and infection with Trypanosoma cruzi. J Nutr 112: 1224-1229.

346. Fralish BH, Tarleton RL (2003) Genetic immunization with LYT1 or a pool of trans-sialidase genes protects mice from lethal Trypanosoma cruzi infection. Vaccine 21: 3070-3080.

347. Franco EL, Sulzer AJ, Higby RW, Peralta JM (1980) Immunoglobulin G and immunoglobulin M polar staining of Toxoplasma gondii in the indirect immunofluorescence test. J Clin Microbiol 12: 780-784.

348. Franke CR, Greiner M, Mehlitz D (1994) Investigations on naturally occurring Trypanosoma evansi infections in horses, cattle, dogs and capybaras (Hydrochaeris hydrochaeris) in Pantanal de Pocone (Mato Grosso, Brazil). Acta Trop 58: 159-169.

349. Freedman BI, Kopp JB, Langefeld CD, Genovese G, Friedman DJ, et al. The apolipoprotein L1 (APOL1) gene and nondiabetic nephropathy in African Americans. J Am Soc Nephrol 21: 1422-1426.

350. Friedhoff KT, Petrich J, Hoffmann M, Buscher G (1984) Trypanosomes in Cervidae in Germany. Zentralbl Bakteriol Mikrobiol Hyg A 256: 286-287.

351. Friedman RK, Bastos FI, Leite IC, Veloso VG, Moreira RI, et al. Pregnancy rates and predictors in women with HIV/AIDS in Rio de Janeiro, Southeastern Brazil. Rev Saude Publica 45: 373-381.

352. Gabbay YB, Glass RI, Monroe SS, Carcamo C, Estes MK, et al. (1994) Prevalence of antibodies to Norwalk virus among Amerindians in isolated Amazonian communities. Am J Epidemiol 139: 728-733.

353. Gabbay YB, Luz CR, Costa IV, Cavalcante-Pepino EL, Sousa MS, et al. (2005) Prevalence and genetic diversity of astroviruses in children with and without diarrhea in Sao Luis, Maranhao, Brazil. Mem Inst Oswaldo Cruz 100: 709-714.

354. Galardo AK, Zimmerman RH, Lounibos LP, Young LJ, Galardo CD, et al. (2009) Seasonal abundance of anopheline mosquitoes and their association with rainfall and malaria along the Matapi River, Amapa, [corrected] Brazil. Med Vet Entomol 23: 335-349.

355. Galel SA, Lifson JD, Engleman EG (1995) Prevention of AIDS transmission through screening of the blood supply. Annu Rev Immunol 13: 201-227.

356. Galhardo MC, De Oliveira RM, Valle AC, Paes Rde A, Silvatavares PM, et al. (2008) Molecular epidemiology and antifungal susceptibility patterns of Sporothrix schenckii isolates from a cat-transmitted epidemic of sporotrichosis in Rio de Janeiro, Brazil. Med Mycol 46: 141-151.

357. Gamboa-Leon R, Gonzalez-Ramirez C, Padilla-Raygoza N, Sosa-Estani S, Caamal-Kantun A, et al. Do commercial serologic tests for Trypanosoma cruzi infection detect Mexican strains in women and newborns? J Parasitol 97: 338-343.

358. Gannavaram S, Sharma P, Duncan RC, Salotra P, Nakhasi HL Mitochondrial associated ubiquitin fold modifier-1 mediated protein conjugation in Leishmania donovani. PLoS One 6: e16156.

359. Garamszegi LZ The sensitivity of microscopy and PCR-based detection methods affecting estimates of prevalence of blood parasites in birds. J Parasitol 96: 1197-1203.

360. Garcia A, Courtin D, Solano P, Koffi M, Jamonneau V (2006) Human African trypanosomiasis: connecting parasite and host genetics. Trends Parasitol 22: 405-409.

361. Garcia-Alvarez A, Sitges M, Heras M, Poyatos S, Posada E, et al. [Endothelial function and high-sensitivity C-reactive protein levels in patients with Chagas disease living in a nonendemic area]. Rev Esp Cardiol 64: 891-896.

362. Garraud O, Andreu G, Elghouzzi MH, Laperche S, Lefrere JJ (2007) Measures to prevent transfusion-associated protozoal infections in non-endemic countries. Travel Med Infect Dis 5: 110-112.

363. Garvin MC, Remsen JV, Jr., Bishop MA, Bennett GF (1993) Hematozoa from passeriform birds in Louisiana. J Parasitol 79: 318-321.

364. Garvin MC, Szell CC, Moore FR (2006) Blood parasites of Nearctic-Neotropical migrant passerine birds during spring trans-Gulf migration: impact on host body condition. J Parasitol 92: 990-996.

365. Garzon E, Genna F, Bosseno MF, Simony-La Fontaine J, Radal M, et al. (2005) Differential infectivity and immunopathology in murine experimental infections by two natural clones belonging to the Trypanosoma cruzi I lineage. Parasitology 131: 109-119.

366. Gautret P, Clerinx J, Caumes E, Simon F, Jensenius M, et al. (2009) Imported human African trypanosomiasis in Europe, 2005-2009. Euro Surveill 14.

367. Gautret P, Cramer JP, Field V, Caumes E, Jensenius M, et al. Infectious diseases among travellers and migrants in Europe, EuroTravNet 2010. Euro Surveill 2012 Jun 28;17(26) pii: 20205.

368. Gazin P, Melo G, Abuquerque A, Oliveira W, Jr., Soula G, et al. (2004) [Chagas disease in a rural area of Northeast Brazil]. Bull Soc Pathol Exot 97: 189-192.

369. Geiger A, Ravel S, Mateille T, Janelle J, Patrel D, et al. (2007) Vector competence of Glossina palpalis gambiensis for Trypanosoma brucei s.l. and genetic diversity of the symbiont Sodalis glossinidius. Mol Biol Evol 24: 102-109.

370. Geiger A, Simo G, Grebaut P, Peltier JB, Cuny G, et al. Transcriptomics and proteomics in human African trypanosomiasis: current status and perspectives. J Proteomics 74: 1625-1643.

371. Genovese G, Friedman DJ, Ross MD, Lecordier L, Uzureau P, et al. Association of trypanolytic ApoL1 variants with kidney disease in African Americans. Science 329: 841-845.

372. Gentilini O, Chagas E, Zurrida S, Intra M, De Cicco C, et al. (2007) Sentinel lymph node biopsy in male patients with early breast cancer. Oncologist 12: 512-515.

373. Gerli R, Caponi L, Tincani A, Scorza R, Sabbadini MG, et al. (2002) Clinical and serological associations of ribosomal P autoantibodies in systemic lupus erythematosus: prospective evaluation in a large cohort of Italian patients. Rheumatology (Oxford) 41: 1357-1366.

374. Germain M, Goldman M (2002) Blood donor selection and screening: strategies to reduce recipient risk. Am J Ther 9: 406-410.

375. Ghedin E, Zhang WW, Charest H, Sundar S, Kenney RT, et al. (1997) Antibody response against a Leishmania donovani amastigote-stage-specific protein in patients with visceral leishmaniasis. Clin Diagn Lab Immunol 4: 530-535.

376. Giammarino M, Vaschetti G, Boano G (2007) Blood parasites in birds from Burkina Faso. Parassitologia 49: 55-57.

377. Gibson W, Pilkington JG, Pemberton JM Trypanosoma melophagium from the sheep ked Melophagus ovinus on the island of St Kilda. Parasitology 137: 1799-1804.

378. Ginsberg R, Ackley A, Stoner E, Lee L (1986) African sleeping sickness presenting in an American emergency department. Ann Emerg Med 15: 86-88.

379. Girones N, Bueno JL, Carrion J, Fresno M, Castro E (2006) The efficacy of photochemical treatment with methylene blue and light for the reduction of Trypanosoma cruzi in infected plasma. Vox Sang 91: 285-291.

380. Glaser B, Gothe R (1998) [Imported arthropod-borne parasites and parasitic arthropods in dogs. Species spectrum and epidemiologic analysis of the cases diagnosed in 1995/96]. Tierarztl Prax Ausg K Kleintiere Heimtiere 26: 40-46.

381. Godfrey DG, Baker RD, Rickman LR, Mehlitz D (1990) The distribution, relationships and identification of enzymic variants within the subgenus Trypanozoon. Adv Parasitol 29: 1-74.

382. Goldrick BA (2004) Chagas disease. Am J Nurs 104: 27.

383. Goldsmith RS, Zarate RJ, Zarate LG, Kagan I, Jacobson LB (1985) Clinical and epidemiologic studies of Chagas' disease in rural communities in Oaxaca State, Mexico, and a seven-year follow-up: I. Cerro del Aire. Bull Pan Am Health Organ 19: 120-138.

384. Goldsmith RS, Zarate RJ, Zarate LG, Morales G, Kagan I, et al. (1992) Clinical and epidemiologic studies of Chagas' disease in rural communities of Oaxaca, Mexico, and an eight-year followup: II. Chila. Bull Pan Am Health Organ 26: 47-59.

385. Goncalez TT, Sabino EC, Murphy EL, Chen S, Chamone DA, et al. (2006) Human immunodeficiency virus test-seeking motivation in blood donors, Sao Paulo, Brazil. Vox Sang 90: 170-176.

386. Gonzalez C, Paz A, Ferro C Predicted altitudinal shifts and reduced spatial distribution of Leishmania infantum vector species under climate change scenarios in Colombia. Acta Trop 2014 Jan;129:83-90 doi: 101016/jactatropica201308014 Epub 2013 Aug 26.

387. Goossens B, Mbwambo H, Msangi A, Geysen D, Vreysen M (2006) Trypanosomosis prevalence in cattle on Mafia Island (Tanzania). Vet Parasitol 139: 74-83.

388. Gottlieb P, Shen LG, Chimezie E, Bahng S, Kenney ME, et al. (1995) Inactivation of Trypanosoma cruzi trypomastigote forms in blood components by photodynamic treatment with phthalocyanines. Photochem Photobiol 62: 869-874.

389. Gourbiere S, Dumonteil E, Rabinovich JE, Minkoue R, Menu F (2008) Demographic and dispersal constraints for domestic infestation by non-domicilated chagas disease vectors in the Yucatan Peninsula, Mexico. Am J Trop Med Hyg 78: 133-139.

390. Gouteux JP, Artzrouni M (2000) [Persistence and resurgence of sleeping sickness caused by Trypanosoma brucei gambiense in historic foci. Biomathematical approach of an epidemiologic enigma]. C R Acad Sci III 323: 351-364.

391. Gouteux JP, Kounda Gboumbi JC, D'Amico F, Wagner C, Noutoua L, et al. (1993) [An epidemiological survey to discover the probable places of infection with sleeping sickness in the Central African Republic]. Bull World Health Organ 71: 605-614.

392. Gouteux JP, Le Gall F, Guillerme JM, Demba D (1996) [Insecticide treatment (Pour on and Spot on) of cattle against Glossina fuscipes fuscipes in the Central African Republic]. Vet Res 27: 273-284.

393. Gow AG, Simpson JW, Picozzi K (2007) First report of canine African trypanosomosis in the UK. J Small Anim Pract 48: 658-661.

394. Grace D, Randolph T, Diall O, Clausen PH (2008) Training farmers in rational drug-use improves their management of cattle trypanosomosis: a cluster-randomised trial in south Mali. Prev Vet Med 83: 83-97.

395. Gracio MA, Gracio AJ, Viveiros M, Amaral L (2003) Since phenothiazines alter antibiotic susceptibility of microorganisms by inhibiting efflux pumps, are these agents useful for evaluating similar pumps in phenothiazine-sensitive parasites? Int J Antimicrob Agents 22: 347-351.

396. Grady SC, Messina JP, McCord PF Population vulnerability and disability in Kenya's tsetse fly habitats. PLoS Negl Trop Dis 5: e957.

397. Grebaut P, Mbida JA, Kondjio CA, Njiokou F, Penchenier L, et al. (2004) Spatial and temporal patterns of human African trypanosomosis (HAT) transmission risk in the Bipindi focus, in the forest zone of southern Cameroon. Vector Borne Zoonotic Dis 4: 230-238.

398. Greenblatt HC, Diggs CL, Rosenstreich DL (1984) Trypanosoma rhodesiense: analysis of the genetic control of resistance among mice. Infect Immun 44: 107-111.

399. Greenblatt HC, Potter TA, Rosenstreich DL (1985) Genetic control of natural resistance to Trypanosoma rhodesiense: transfer of resistance with bone marrow or spleen cells. J Infect Dis 151: 911-916.

400. Greiner M, Bhat TS, Patzelt RJ, Kakaire D, Schares G, et al. (1997) Impact of biological factors on the interpretation of bovine trypanosomosis serology. Prev Vet Med 30: 61-73.

401. Greiner M, Franke CR, Bohning D, Schlattmann P (1994) Construction of an intrinsic cut-off value for the sero-epidemiological study of Trypanosoma evansi infections in a canine population in Brazil: a new approach towards an unbiased estimation of prevalence. Acta Trop 56: 97-109.

402. Greiner M, Kumar S, Kyeswa C (1997) Evaluation and comparison of antibody ELISAs for serodiagnosis of bovine trypanosomosis. Vet Parasitol 73: 197-205.

403. Greiner M, Mattioli RC, Faye J, Rebeski D, Winger E, et al. (2001) A survival analysis of trypanosomosis diagnostic-test performance under natural infection challenge. Prev Vet Med 51: 51-62.

404. Grellier P, Santus R, Mouray E, Agmon V, Maziere JC, et al. (1997) Photosensitized inactivation of Plasmodium falciparum- and Babesia divergens-infected erythrocytes in whole blood by lipophilic pheophorbide derivatives. Vox Sang 72: 211-220.

405. Grieves JL, Hubbard GB, Williams JT, Vandeberg JL, Dick EJ, Jr., et al. (2008) Trypanosoma cruzi in non-human primates with a history of stillbirths: a retrospective study (Papio hamadryas spp.) and case report (Macaca fascicularis). J Med Primatol 37: 318-328.

406. Grimaldi A, Alfieri O, Camici PG, La Canna G, Zoppei G, et al. ["African sickness" and the heart: the mystery of endomyocardial fibrosis]. G Ital Cardiol (Rome) 12: 484-491.

407. Grinsztejn B, Veloso VG, Friedman RK, Moreira RI, Luz PM, et al. (2009) Early mortality and cause of deaths in patients using HAART in Brazil and the United States. AIDS 23: 2107-2114.

408. Grinsztejn B, Veloso VG, Pilotto JH, Campos DP, Keruly JC, et al. (2007) Comparison of clinical response to initial highly active antiretroviral therapy in the patients in clinical care in the United States and Brazil. J Acquir Immune Defic Syndr 45: 515-520.

409. Grogl M, Kuhn RE, Davis DS, Green GE (1984) Antibodies to Trypanosoma cruzi in coyotes in texas. J Parasitol 70: 189-191.

410. Grosjean NL, Vrable RA, Murphy AJ, Mansfield LS (2003) Seroprevalence of antibodies against Leishmania spp among dogs in the United States. J Am Vet Med Assoc 222: 603-606.

411. Gross R (1978) [Change in infectious diseases: an introduction]. Internist (Berl) 19: 145-146.

412. Grosso NL, Bua J, Perrone AE, Gonzalez MN, Bustos PL, et al. Trypanosoma cruzi: biological characterization of a isolate from an endemic area and its susceptibility to conventional drugs. Exp Parasitol 126: 239-244.

413. Guernaoui S, Ramaoui K, Rahola N, Barnabe C, Sereno D, et al. Malformations of the genitalia in male Phlebotomus papatasi (Scopoli) (Diptera: Psychodidae). J Vector Ecol 35: 13-19.

414. Guertler L (2002) Virus safety of human blood, plasma, and derived products. Thromb Res 107 Suppl 1: S39-45.

415. Guhl F, Restrepo M, Angulo VM, Antunes CM, Campbell-Lendrum D, et al. (2005) Lessons from a national survey of Chagas disease transmission risk in Colombia. Trends Parasitol 21: 259-262.

416. Gurevitz JM, Ceballos LA, Gaspe MS, Alvarado-Otegui JA, Enriquez GF, et al. Factors affecting infestation by Triatoma infestans in a rural area of the humid Chaco in Argentina: a multi-model inference approach. PLoS Negl Trop Dis 5: e1349.

417. Gurevitz JM, Gaspe MS, Enriquez GF, Provecho YM, Kitron U, et al. Intensified surveillance and insecticide-based control of the Chagas disease vector Triatoma infestans in the Argentinean Chaco. PLoS Negl Trop Dis 2013 Apr 11;7(4):e2158 doi: 101371/journalpntd0002158 Print 2013.

418. Gurski KC, Ebbert MA (2003) Host age, but not host location within a stream, is correlated with the prevalence of gut parasites in water striders. J Parasitol 89: 529-534.

419. Gurtler RE (2009) Sustainability of vector control strategies in the Gran Chaco Region: current challenges and possible approaches. Mem Inst Oswaldo Cruz 104 Suppl 1: 52-59.

420. Gurtler RE, Ceballos LA, Ordonez-Krasnowski P, Lanati LA, Stariolo R, et al. (2009) Strong host-feeding preferences of the vector Triatoma infestans modified by vector density: implications for the epidemiology of Chagas disease. PLoS Negl Trop Dis 3: e447.

421. Gurtler RE, Ceballos LA, Stariolo R, Kitron U, Reithinger R (2009) Effects of topical application of fipronil spot-on on dogs against the Chagas disease vector Triatoma infestans. Trans R Soc Trop Med Hyg 103: 298-304.

422. Gurtler RE, Cecere MC, Lauricella MA, Cardinal MV, Kitron U, et al. (2007) Domestic dogs and cats as sources of Trypanosoma cruzi infection in rural northwestern Argentina. Parasitology 134: 69-82.

423. Gurtler RE, Cecere MC, Lauricella MA, Petersen RM, Chuit R, et al. (2005) Incidence of trypanosoma cruzi infection among children following domestic reinfestation after insecticide spraying in rural northwestern Argentina. Am J Trop Med Hyg 73: 95-103.

424. Gurtler RE, Kitron U, Cecere MC, Segura EL, Cohen JE (2007) Sustainable vector control and management of Chagas disease in the Gran Chaco, Argentina. Proc Natl Acad Sci U S A 104: 16194-16199.

425. Gushulak BD, MacPherson DW Health aspects of the pre-departure phase of migration. PLoS Med 8: e1001035.

426. Gutierrez C, Corbera JA, Juste MC, Doreste F, Morales I (2005) An outbreak of abortions and high neonatal mortality associated with Trypanosoma evansi infection in dromedary camels in the Canary Islands. Vet Parasitol 130: 163-168.

427. Gutierrez C, Corbera JA, Juste MC, Doreste F, Morales I (2006) Clinical, hematological, and biochemical findings in an outbreak of abortion and neonatal mortality associated with Trypanosoma evansi infection in dromedary camels. Ann N Y Acad Sci 1081: 325-327.

428. Gutierrez C, Corbera JA, Morales M, Buscher P (2006) Trypanosomosis in goats: current status. Ann N Y Acad Sci 1081: 300-310.

429. Gutierrez C, Desquesnes M, Touratier L, Buscher P Trypanosoma evansi: recent outbreaks in Europe. Vet Parasitol 174: 26-29.

430. Gutierrez C, Juste MC, Corbera JA, Magnus E, Verloo D, et al. (2000) Camel trypanosomosis in the Canary Islands: assessment of seroprevalence and infection rates using the card agglutination test (CATT/T. evansi) and parasite detection tests. Vet Parasitol 90: 155-159.

431. Guz N, Attardo GM, Wu Y, Aksoy S (2007) Molecular aspects of transferrin expression in the tsetse fly (Glossina morsitans morsitans). J Insect Physiol 53: 715-723.

432. Haas M, Lukan M, Kiskova J, Hrehova Z Occurrence of blood parasites and intensity of infection in Prunella modularis in the montane and subalpine zone in the Slovak Carpathians. Acta Parasitol 2012 Sep;57(3):221-7 doi: 102478/s11686-012-0041-6 Epub 2012 Aug 9.

433. Hagen MO, Garcia-Garcia E, Oladiran A, Karpman M, Mitchell S, et al. The acute and sub-chronic exposures of goldfish to naphthenic acids induce different host defense responses. Aquat Toxicol 2012 Mar;109:143-9 doi: 101016/jaquatox201112011 Epub 2011 Dec 23.

434. Haines LR, Lehane SM, Pearson TW, Lehane MJ Tsetse EP protein protects the fly midgut from trypanosome establishment. PLoS Pathog 6: e1000793.

435. Hamilton PB, Adams ER, Njiokou F, Gibson WC, Cuny G, et al. (2009) Phylogenetic analysis reveals the presence of the Trypanosoma cruzi clade in African terrestrial mammals. Infect Genet Evol 9: 81-86.

436. Hamilton PB, Lewis MD, Cruickshank C, Gaunt MW, Yeo M, et al. Identification and lineage genotyping of South American trypanosomes using fluorescent fragment length barcoding. Infect Genet Evol 11: 44-51.

437. Hancock K, Zajac AM, Pung OJ, Elvinger F, Rosypal AC, et al. (2005) Prevalence of antibodies to Trypanosoma cruzi in raccoons (Procyon lotor) from an urban area of northern Virginia. J Parasitol 91: 470-472.

438. Hanly JG, Walsh NM, Fisk JD, Eastwood B, Hong C, et al. (1993) Cognitive impairment and autoantibodies in systemic lupus erythematosus. Br J Rheumatol 32: 291-296.

439. Hao Z, Aksoy S (2002) Proventriculus-specific cDNAs characterized from the tsetse, Glossina morsitans morsitans. Insect Biochem Mol Biol 32: 1663-1671.

440. Hao Z, Kasumba I, Aksoy S (2003) Proventriculus (cardia) plays a crucial role in immunity in tsetse fly (Diptera: Glossinidiae). Insect Biochem Mol Biol 33: 1155-1164.

441. Hao Z, Kasumba I, Lehane MJ, Gibson WC, Kwon J, et al. (2001) Tsetse immune responses and trypanosome transmission: implications for the development of tsetse-based strategies to reduce trypanosomiasis. Proc Natl Acad Sci U S A 98: 12648-12653.

442. Hardison JL, Wrightsman RA, Carpenter PM, Kuziel WA, Lane TE, et al. (2006) The CC chemokine receptor 5 is important in control of parasite replication and acute cardiac inflammation following infection with Trypanosoma cruzi. Infect Immun 74: 135-143.

443. Harms G, Feldmeier H (2002) HIV infection and tropical parasitic diseases - deleterious interactions in both directions? Trop Med Int Health 7: 479-488.

444. Harms G, Feldmeier H (2005) The impact of HIV infection on tropical diseases. Infect Dis Clin North Am 19: 121-135, ix.

445. Hart W, Slee PH, Schipper HG, Koopmans RP, Kager PA (2004) [Clinical reasoning and decision making in practice. A depressive foreign woman with symptoms of malaise]. Ned Tijdschr Geneeskd 148: 771-776.

446. Harvey K, Esposito DH, Han P, Kozarsky P, Freedman DO, et al. Surveillance for travel-related disease--GeoSentinel Surveillance System, United States, 1997-2011. MMWR Surveill Summ 2013 Jul 19;62:1-23.

447. Hay SI, Myers MF, Burke DS, Vaughn DW, Endy T, et al. (2000) Etiology of interepidemic periods of mosquito-borne disease. Proc Natl Acad Sci U S A 97: 9335-9339.

448. Hay SI, Rogers DJ, Shanks GD, Myers MF, Snow RW (2001) Malaria early warning in Kenya. Trends Parasitol 17: 95-99.

449. Hay SI, Snow RW, Rogers DJ (1998) From predicting mosquito habitat to malaria seasons using remotely sensed data: practice, problems and perspectives. Parasitol Today 14: 306-313.

450. Hay SI, Snow RW, Rogers DJ (1998) Predicting malaria seasons in Kenya using multitemporal meteorological satellite sensor data. Trans R Soc Trop Med Hyg 92: 12-20.

451. Healing TD (1981) Infections with blood parasites in the small British rodents Apodemus sylvaticus, Clethrionomys glareolus and Microtus agrestis. Parasitology 83: 179-189.

452. Helliwell CJ, Turner AC (1980) Imported disease at point of entry. Practitioner 224: 793-796.

453. Hendrickx G, Napala A, Slingenbergh JH, De Deken R, Rogers DJ (2001) A contribution towards simplifying area-wide tsetse surveys using medium resolution meteorological satellite data. Bull Entomol Res 91: 333-346.

454. Herda LR, Felix SB, Boege F Drug-like actions of autoantibodies against receptors of the autonomous nervous system and their impact on human heart function. Br J Pharmacol 2012 Jun;166(3):847-57 doi: 101111/j1476-5381201201828x.

455. Hide G (1999) History of sleeping sickness in East Africa. Clin Microbiol Rev 12: 112-125.

456. Hide G, Angus SD, Holmes PH, Maudlin I, Welburn SC (1998) Trypanosoma brucei: comparison of circulating strains in an endemic and an epidemic area of a sleeping sickness focus. Exp Parasitol 89: 21-29.

457. Hide G, Tait A (1991) The molecular epidemiology of parasites. Experientia 47: 128-142.

458. Hide G, Tilley A, Welburn SC, Maudlin I, Tait A (2000) Trypanosoma brucei: identification of trypanosomes with genotypic similarity to human infective isolates in tsetse isolated from a region free of human sleeping sickness. Exp Parasitol 96: 67-74.

459. Hide G, Welburn SC, Tait A, Maudlin I (1994) Epidemiological relationships of Trypanosoma brucei stocks from south east Uganda: evidence for different population structures in human infective and non-human infective isolates. Parasitology 109 ( Pt 1): 95-111.

460. Hidron AI, Gilman RH, Justiniano J, Blackstock AJ, Lafuente C, et al. Chagas cardiomyopathy in the context of the chronic disease transition. PLoS Negl Trop Dis 4: e688.

461. Hochberg NS, Moro RN, Sheth AN, Montgomery SP, Steurer F, et al. High prevalence of persistent parasitic infections in foreign-born, HIV-infected persons in the United States. PLoS Negl Trop Dis 5: e1034.

462. Hoffmann M, Buscher G, Friedhoff KT (1984) Stercorarian trypanosomes from deer (Cervidae) in Germany. J Protozool 31: 581-584.

463. Hohenschild S (1999) [Babesiosis--a dangerous infection for splenectomized children and adults]. Klin Padiatr 211: 137-140.

464. Holthoff HP, Zeibig S, Jahns-Boivin V, Bauer J, Lohse MJ, et al. Detection of anti-beta1-AR autoantibodies in heart failure by a cell-based competition ELISA. Circ Res 2012 Aug 31;111(6):675-84 doi: 101161/CIRCRESAHA112272682 Epub 2012 Jul 18.

465. Hontebeyrie-Joskowicz M (1992) Immunoregulatory mechanisms and Chagas' disease. Mem Inst Oswaldo Cruz 87 Suppl 5: 101-103.

466. Hope-Rapp E, Moussa Coulibaly O, Klement E, Danis M, Bricaire F, et al. (2009) [Double trypanosomal chancre revealing West African trypanosomiasis in a Frenchman living in Gabon]. Ann Dermatol Venereol 136: 341-345.

467. Hoppenheit A, Bauer B, Steuber S, Terhalle W, Diall O, et al. Multiple host feeding in Glossina palpalis gambiensis and Glossina tachinoides in southeast Mali. Med Vet Entomol 2013 Jun;27(2):222-5 doi: 101111/j1365-2915201201046x Epub 2012 Sep 25.

468. Hotez P Enlarging the "Audacious Goal": elimination of the world's high prevalence neglected tropical diseases. Vaccine 2011 Dec 30;29 Suppl 4:D104-10 doi: 101016/jvaccine201106024 Epub 2011 Dec 19.

469. Hotez P A handful of 'antipoverty' vaccines exist for neglected diseases, but the world's poorest billion people need more. Health Aff (Millwood) 30: 1080-1087.

470. Hotez PJ (2008) Neglected infections of poverty in the United States of America. PLoS Negl Trop Dis 2: e256.

471. Hotez PJ, Gurwith M Europe's neglected infections of poverty. Int J Infect Dis 15: e611-619.

472. Hotez PJ, Kamath A (2009) Neglected tropical diseases in sub-saharan Africa: review of their prevalence, distribution, and disease burden. PLoS Negl Trop Dis 3: e412.

473. Houghton RL, Benson DR, Reynolds L, McNeill P, Sleath P, et al. (2000) Multiepitope synthetic peptide and recombinant protein for the detection of antibodies to Trypanosoma cruzi in patients with treated or untreated Chagas' disease. J Infect Dis 181: 325-330.

474. Houghton RL, Stevens YY, Hjerrild K, Guderian J, Okamoto M, et al. (2009) Lateral flow immunoassay for diagnosis of Trypanosoma cruzi infection with high correlation to the radioimmunoprecipitation assay. Clin Vaccine Immunol 16: 515-520.

475. Houk AE, Goodwin DG, Zajac AM, Barr SC, Dubey JP, et al. Prevalence of antibodies to Trypanosoma cruzi, Toxoplasma gondii, Encephalitozoon cuniculi, Sarcocystis neurona, Besnoitia darlingi, and Neospora caninum in North American opossums, Didelphis virginiana, from southern Louisiana. J Parasitol 96: 1119-1122.

476. Hoyte HM (1972) The morphology of Trypanosoma theileri in the blood of cattle, and the rediscovery of Theileria mutans in England. Z Parasitenkd 38: 183-199.

477. Hu C, Aksoy S (2006) Innate immune responses regulate trypanosome parasite infection of the tsetse fly Glossina morsitans morsitans. Mol Microbiol 60: 1194-1204.

478. Hu C, Rio RV, Medlock J, Haines LR, Nayduch D, et al. (2008) Infections with immunogenic trypanosomes reduce tsetse reproductive fitness: potential impact of different parasite strains on vector population structure. PLoS Negl Trop Dis 2: e192.

479. Hu Y, Aksoy S (2005) An antimicrobial peptide with trypanocidal activity characterized from Glossina morsitans morsitans. Insect Biochem Mol Biol 35: 105-115.

480. Huang TL, Bacchi CJ, Kode NR, Zhang Q, Wang G, et al. (2007) Trypanocidal activity of piperazine-linked bisbenzamidines and bisbenzamidoxime, an orally active prodrug. Int J Antimicrob Agents 30: 555-561.

481. Hubalek Z, Stunzner D, Halouzka J, Sixl W, Wendelin I, et al. (2003) Prevalence of borreliae in ixodid ticks from a floodplain forest ecosystem. Wien Klin Wochenschr 115: 121-124.

482. Hulsey M, Goldstein R, Scully L, Surbeck W, Reichlin M (1995) Anti-ribosomal P antibodies in systemic lupus erythematosus: a case-control study correlating hepatic and renal disease. Clin Immunol Immunopathol 74: 252-256.

483. Hunter CA, Ellis-Neyes LA, Slifer T, Kanaly S, Grunig G, et al. (1997) IL-10 is required to prevent immune hyperactivity during infection with Trypanosoma cruzi. J Immunol 158: 3311-3316.

484. Hunter GC, Borrini-Mayori K, Ancca Juarez J, Castillo Neyra R, Verastegui MR, et al. A field trial of alternative targeted screening strategies for Chagas disease in Arequipa, Peru. PLoS Negl Trop Dis 2012 Jan;6(1):e1468 doi: 101371/journalpntd0001468 Epub 2012 Jan 10.

485. Imhoof B, Schmid-Hempel P (1998) Single-clone and mixed-clone infections versus host environment in Crithidia bombi infecting bumblebees. Parasitology 117 ( Pt 4): 331-336.

486. Imrie HJ, Fowkes FJ, Migot-Nabias F, Luty AJ, Deloron P, et al. Individual variation in levels of haptoglobin-related protein in children from Gabon. PLoS One 2012;7(11):e49816 doi: 101371/journalpone0049816 Epub 2012 Nov 20.

487. Inogwabini BI, Leader-Williams N Effects of epidemic diseases on the distribution of bonobos. PLoS One 2012;7(12):e51112 doi: 101371/journalpone0051112 Epub 2012 Dec 12.

488. Intra M, Gentilini O, Brenelli F, Chagas EM, Veronesi U, et al. (2005) Breast cancer among HIV-infected patients: the experience of the European Institute of Oncology. J Surg Oncol 91: 141-142.

489. Iori A, Lanfranchi P, Manilla G (1996) Contribution to the knowledge of Ixodidae ticks of wild mammals of Somalia. Parassitologia 38: 571-573.

490. Ismach R, Cianci CM, Caulfield JP, Langer PJ, Hein A, et al. (1989) Flagellar membrane and paraxial rod proteins of Leishmania: characterization employing monoclonal antibodies. J Protozool 36: 617-624.

491. Iten M, Matovu E, Brun R, Kaminsky R (1995) Innate lack of susceptibility of Ugandan Trypanosoma brucei rhodesiense to DL-alpha-difluoromethylornithine (DFMO). Trop Med Parasitol 46: 190-194.

492. Jabari S, da Silveira AB, de Oliveira EC, Neto SG, Quint K, et al. Selective survival of calretinin- and vasoactive-intestinal-peptide-containing nerve elements in human chagasic submucosa and mucosa. Cell Tissue Res 2012 Aug;349(2):473-81 doi: 101007/s00441-012-1406-8 Epub 2012 May 5.

493. James SL (1997) Emerging parasitic infections. FEMS Immunol Med Microbiol 18: 313-317.

494. Jamonneau V, Bucheton B, Kabore J, Ilboudo H, Camara O, et al. Revisiting the immune trypanolysis test to optimise epidemiological surveillance and control of sleeping sickness in West Africa. PLoS Negl Trop Dis 4: e917.

495. Jamonneau V, Ilboudo H, Kabore J, Kaba D, Koffi M, et al. Untreated human infections by Trypanosoma brucei gambiense are not 100% fatal. PLoS Negl Trop Dis 2012;6(6):e1691 doi: 101371/journalpntd0001691 Epub 2012 Jun 12.

496. Jamonneau V, N'Guessan P, N'Dri L, Simarro P, Truc P (2000) Exploration of the distribution of Trypanosoma brucei ssp. in West Africa, by multilocus enzyme electrophoresis. Ann Trop Med Parasitol 94: 643-649.

497. Jamonneau V, Ravel S, Garcia A, Koffi M, Truc P, et al. (2004) Characterization of Trypanosoma brucei s.l. infecting asymptomatic sleeping-sickness patients in Cote d'Ivoire: a new genetic group? Ann Trop Med Parasitol 98: 329-337.

498. Jamonneau V, Ravel S, Koffi M, Kaba D, Zeze DG, et al. (2004) Mixed infections of trypanosomes in tsetse and pigs and their epidemiological significance in a sleeping sickness focus of Cote d'Ivoire. Parasitology 129: 693-702.

499. Jamonneau V, Truc P, Garcia A, Magnus E, Buscher P (2000) Preliminary evaluation of LATEX/T. b. gambiense and alternative versions of CATT/T. b. gambiense for the serodiagnosis of human african trypanosomiasis of a population at risk in Cote d'Ivoire: considerations for mass-screening. Acta Trop 76: 175-183.

500. Jannin J (2009) [Presentation of the consensus workshop about the Chagas disease in non-endemic areas (26 June 2009, Paris, France)]. Bull Soc Pathol Exot 102: 275.

501. Jannin JG (2005) Sleeping sickness--a growing problem? BMJ 331: 1242.

502. Jaskoski BJ, Plank JD (1967) Incidence of endoparasitism in a group of pigeons in the Chicago area. Avian Dis 11: 342-344.

503. Jaynes JM, Burton CA, Barr SB, Jeffers GW, Julian GR, et al. (1988) In vitro cytocidal effect of novel lytic peptides on Plasmodium falciparum and Trypanosoma cruzi. FASEB J 2: 2878-2883.

504. Jelinek T, Bisoffi Z, Bonazzi L, van Thiel P, Bronner U, et al. (2002) Cluster of African trypanosomiasis in travelers to Tanzanian national parks. Emerg Infect Dis 8: 634-635.

505. Jelinek T, Muhlberger N (2005) Surveillance of imported diseases as a window to travel health risks. Infect Dis Clin North Am 19: 1-13.

506. Jessop EG (2006) Another public health triumph. J Public Health (Oxf) 28: 297-298.

507. Johnson D, Harms NJ, Larter NC, Elkin BT, Tabel H, et al. Serum biochemistry, serology, and parasitology of boreal caribou (Rangifer tarandus caribou) in the Northwest Territories, Canada. J Wildl Dis 46: 1096-1107.

508. Johnston V, Stockley JM, Dockrell D, Warrell D, Bailey R, et al. (2009) Fever in returned travellers presenting in the United Kingdom: recommendations for investigation and initial management. J Infect 59: 1-18.

509. Johnstone DB, Shegokar V, Nihalani D, Rathore YS, Mallik L, et al. APOL1 null alleles from a rural village in India do not correlate with glomerulosclerosis. PLoS One 2012;7(12):e51546 doi: 101371/journalpone0051546 Epub 2012 Dec 26.

510. Jones DC, Alphey MS, Wyllie S, Fairlamb AH Chemical, genetic and structural assessment of pyridoxal kinase as a drug target in the African trypanosome. Mol Microbiol 2012 Oct;86(1):51-64 doi: 101111/j1365-2958201208189x Epub 2012 Aug 16.

511. Jones J (2000) African sleeping sickness returns to UK after four years. BMJ 321: 1177.

512. Julian RJ (1971) The isolation of trypanosomes from cattle in Ontario. Can J Comp Med 35: 192-194.

513. Kabiri M, Franco JR, Simarro PP, Ruiz JA, Sarsa M, et al. (1999) Detection of Trypanosoma brucei gambiense in sleeping sickness suspects by PCR amplification of expression-site-associated genes 6 and 7. Trop Med Int Health 4: 658-661.

514. Kabore J, Macleod A, Jamonneau V, Ilboudo H, Duffy C, et al. Population genetic structure of Guinea Trypanosoma brucei gambiense isolates according to host factors. Infect Genet Evol 11: 1129-1135.

515. Kadohira M, McDermott JJ, Shoukri MM, Thorburn MA (1997) Assessing infections at multiple levels of aggregation. Prev Vet Med 29: 161-177.

516. Kaiser A, Gottwald A, Wiersch C, Maier W, Seitz HM (2002) The necessity to develop drugs against parasitic diseases. Pharmazie 57: 723-728.

517. Karbowiak G, Wita I (2001) [The cases of infection of brown rats Rattus norvegicus with Trypanosoma lewisi (Kent, 1880) Laveran i Mesnil, 1901, in the area of Warsaw agglomeration]. Wiad Parazytol 47: 377-382.

518. Karbowiak G, Wita I (2001) [Ecological aspects of infection of bank vole Clethrionomys glareolus (Schreber, 1780) with Trypanosoma (Herpetosoma) evotomys Hadwen, 1912]. Wiad Parazytol 47: 789-795.

519. Karbowiak G, Wita I, Czaplinska U (2009) The occurrence and ultrastructure of Trypanosoma (Herpetosoma) lewisi (Kent, 1880) Laveran and Mesnil, 1901, the parasite of rats (Rattus norvegicus) in Poland. Wiad Parazytol 55: 249-258.

520. Karimuribo ED, Morrison LJ, Black A, Turner CM, Kambarage DM, et al. Analysis of host genetic factors influencing African trypanosome species infection in a cohort of Tanzanian Bos indicus cattle. Vet Parasitol 179: 35-42.

521. Karsten V, Davis C, Kuhn R (1992) Trypanosoma cruzi in wild raccoons and opossums in North Carolina. J Parasitol 78: 547-549.

522. Katunguka-Rwakishaya E, Murray M, Holmes PH (1997) Susceptibility of three breeds of Ugandan goats to experimental infection with Trypanosoma congolense. Trop Anim Health Prod 29: 7-14.

523. Katzenback BA, Plouffe DA, Belosevic M Goldfish (Carassius auratus L.) possess natural antibodies with trypanocidal activity towards Trypanosoma carassii in vitro. Fish Shellfish Immunol 2013 May;34(5):1025-32 doi: 101016/jfsi201212018 Epub 2013 Jan 18.

524. Kaufmann J, Dwinger RH, Hallebeek A, van Dijk B, Pfister K (1992) The interaction of Trypanosoma congolense and Haemonchus contortus infections in trypanotolerant N'Dama cattle. Vet Parasitol 43: 157-170.

525. Keck N, Herder S, Kaba D, Solano P, Gomez J, et al. (2009) Epidemiological study of canine trypanosomosis in an urban area of Ivory Coast. Parasite 16: 305-308.

526. Keku TO, Seed JR, Sechelski JB, Balber A (1993) Trypanosoma brucei rhodesiense: the inhibition of HL-60 cell growth by the African trypanosomes in vitro. Exp Parasitol 77: 306-314.

527. Khan OA, Davenhall W, Ali M, Castillo-Salgado C, Vazquez-Prokopec G, et al. Geographical information systems and tropical medicine. Ann Trop Med Parasitol 104: 303-318.

528. Khan RA (1988) Experimental transmission, development, and effects of a parasitic copepod, Lernaeocera branchialis, on Atlantic cod, Gadus morhua. J Parasitol 74: 586-599.

529. Khonde N, Pepin J, Niyonsenga T, De Wals P (1997) Familial aggregation of Trypanosoma brucei gambiense trypanosomiasis in a very high incidence community in Zaire. Trans R Soc Trop Med Hyg 91: 521-524.

530. Khonde N, Pepin J, Niyonsenga T, Milord F, De Wals P (1995) Epidemiological evidence for immunity following Trypanosoma brucei gambiense sleeping sickness. Trans R Soc Trop Med Hyg 89: 607-611.

531. King RJ, Cordon-Rosales C, Cox J, Davies CR, Kitron UD Triatoma dimidiata infestation in Chagas disease endemic regions of Guatemala: comparison of random and targeted cross-sectional surveys. PLoS Negl Trop Dis 5: e1035.

532. Kingston N (1991) A brief review of Trypanosoma (Megatrypanum) infections in ruminants in North America and Europe. Wiad Parazytol 37: 211-218.

533. Kioy D, Mattock N (2005) Control of sleeping sickness--time to integrate approaches. Lancet 366: 695-696.

534. Kirchhoff LV (1993) Chagas disease. American trypanosomiasis. Infect Dis Clin North Am 7: 487-502.

535. Kirchhoff LV (1993) American trypanosomiasis (Chagas' disease)--a tropical disease now in the United States. N Engl J Med 329: 639-644.

536. Kirkpatrick CE, Lauer DM (1985) Hematozoa of raptors from southern New Jersey and adjacent areas. J Wildl Dis 21: 1-6.

537. Kistner TP, Hanson WL (1969) Trypanosomiasis in white-tailed deer. Wildl Dis 5: 398-399.

538. Kitron U, Clennon JA, Cecere MC, Gurtler RE, King CH, et al. (2006) Upscale or downscale: applications of fine scale remotely sensed data to Chagas disease in Argentina and schistosomiasis in Kenya. Geospat Health 1: 49-58.

539. Kjos SA, Marcet PL, Yabsley MJ, Kitron U, Snowden KF, et al. Identification of bloodmeal sources and Trypanosoma cruzi infection in triatomine bugs (Hemiptera: Reduviidae) from residential settings in Texas, the United States. J Med Entomol 2013 Sep;50(5):1126-39.

540. Kjos SA, Snowden KF, Craig TM, Lewis B, Ronald N, et al. (2008) Distribution and characterization of canine Chagas disease in Texas. Vet Parasitol 152: 249-256.

541. Kjos SA, Snowden KF, Olson JK (2009) Biogeography and Trypanosoma cruzi infection prevalence of Chagas disease vectors in Texas, USA. Vector Borne Zoonotic Dis 9: 41-50.

542. Kleinman S, King MR, Busch MP, Murphy EL, Glynn SA The National Heart, Lung, and Blood Institute retrovirus epidemiology donor studies (Retrovirus Epidemiology Donor Study and Retrovirus Epidemiology Donor Study-II): twenty years of research to advance blood product safety and availability. Transfus Med Rev 2012 Oct;26(4):281-304, 304e1-2 doi: 101016/jtmrv201204004 Epub 2012 May 24.

543. Kliukiene R, Maroziene A, Cenas N, Becker K, Blanchard JS (1996) Photoinactivation of trypanothione reductase and glutathione reductase by Al-phthalocyanine tetrasulfonate and hematoporphyrin. Biochem Biophys Res Commun 218: 629-632.

544. Klotz SA, Dorn PL, Klotz JH, Pinnas JL, Weirauch C, et al. (2009) Feeding behavior of triatomines from the southwestern United States: an update on potential risk for transmission of Chagas disease. Acta Trop 111: 114-118.

545. Knopper LD, Mineau P (2004) Organismal effects of pesticide exposure on meadow voles (Microtus pennsylvanicus) living in golf course ecosystems: developmental instability, clinical hematology, body condition, and blood parasitology. Environ Toxicol Chem 23: 1512-1519.

546. Ko WY, Rajan P, Gomez F, Scheinfeldt L, An P, et al. Identifying Darwinian selection acting on different human APOL1 variants among diverse African populations. Am J Hum Genet 2013 Jul 11;93(1):54-66 doi: 101016/jajhg201305014 Epub 2013 Jun 13.

547. Kocher C, Segerer S, Schleich A, Caduff R, Wyler LG, et al. Skin lesions, malaise, and heart failure in a renal transplant recipient. Transpl Infect Dis 2012 Aug;14(4):391-7 doi: 101111/j1399-3062201200731x Epub 2012 Apr 8.

548. Koffi M, De Meeus T, Bucheton B, Solano P, Camara M, et al. (2009) Population genetics of Trypanosoma brucei gambiense, the agent of sleeping sickness in Western Africa. Proc Natl Acad Sci U S A 106: 209-214.

549. Koffi M, Solano P, Barnabe C, de Meeus T, Bucheton B, et al. (2007) Genetic characterisation of Trypanosoma brucei s.l. using microsatellite typing: new perspectives for the molecular epidemiology of human African trypanosomiasis. Infect Genet Evol 7: 675-684.

550. Kolev NG, Ramey-Butler K, Cross GA, Ullu E, Tschudi C Developmental progression to infectivity in Trypanosoma brucei triggered by an RNA-binding protein. Science 2012 Dec 7;338(6112):1352-3 doi: 101126/science1229641.

551. Kollien AH, Goncalves TC, De Azambuja P, Garcia ES, Schaub GA (1998) The effect of azadirachtin on fresh isolates of Trypanosoma cruzi in different species of triatomines. Parasitol Res 84: 286-290.

552. Kone N, N'Goran E K, Sidibe I, Kombassere AW, Bouyer J Spatio-temporal distribution of tsetse and other biting flies in the Mouhoun River basin, Burkina Faso. Med Vet Entomol 25: 156-168.

553. Kotsikorou E, Song Y, Chan JM, Faelens S, Tovian Z, et al. (2005) Bisphosphonate inhibition of the exopolyphosphatase activity of the Trypanosoma brucei soluble vacuolar pyrophosphatase. J Med Chem 48: 6128-6139.

554. Krafsur ES (2003) Tsetse fly population genetics: an indirect approach to dispersal. Trends Parasitol 19: 162-166.

555. Krafsur ES (2009) Tsetse flies: genetics, evolution, and role as vectors. Infect Genet Evol 9: 124-141.

556. Kribs-Zaleta C Estimating contact process saturation in sylvatic transmission of Trypanosoma cruzi in the United States. PLoS Negl Trop Dis 4: e656.

557. Kribs-Zaleta CM Alternative transmission modes for Trypanosoma cruzi. Math Biosci Eng 7: 657-673.

558. Kroeger A, Villegas E, Ordonez-Gonzalez J, Pabon E, Scorza JV (2003) Prevention of the transmission of Chagas' disease with pyrethroid-impregnated materials. Am J Trop Med Hyg 68: 307-311.

559. Kuepfer I, Schmid C, Allan M, Edielu A, Haary EP, et al. Safety and efficacy of the 10-day melarsoprol schedule for the treatment of second stage Rhodesiense sleeping sickness. PLoS Negl Trop Dis 2012 Aug;6(8):e1695 doi: 101371/journalpntd0001695 Epub 2012 Aug 28.

560. Kumar S, Tarleton RL (2001) Antigen-specific Th1 but not Th2 cells provide protection from lethal Trypanosoma cruzi infection in mice. J Immunol 166: 4596-4603.

561. Kurland LT (1977) 25 years of neuroepidemiology in the Americas. Neurol Neurocir Psiquiatr 18: 129-144.

562. Kuzoe FA (1993) Current situation of African trypanosomiasis. Acta Trop 54: 153-162.

563. Laakkonen J, Haukisalmi V, Merritt JF (1998) Blood parasites of shrews from Pennsylvania. J Parasitol 84: 1300-1303.

564. Lainson R, Franco CM, Da Matta R Plasmodium carmelinoi n. sp. (Haemosporida: Plasmodiidae) of the lizard Ameiva ameiva (Squamata: Teiidae) in Amazonian Brazil. Parasite 17: 129-132.

565. Lainson R, Rangel EF (2005) Lutzomyia longipalpis and the eco-epidemiology of American visceral leishmaniasis, with particular reference to Brazil: a review. Mem Inst Oswaldo Cruz 100: 811-827.

566. Lancien J (1991) [Campaign against sleeping sickness in South-West Uganda by trapping tsetse flies]. Ann Soc Belg Med Trop 71 Suppl 1: 35-47.

567. Lane JE, Olivares-Villagomez D, Vnencak-Jones CL, McCurley TL, Carter CE (1997) Detection of Trypanosoma cruzi with the polymerase chain reaction and in situ hybridization in infected murine cardiac tissue. Am J Trop Med Hyg 56: 588-595.

568. Larcan A, Huriet C (1964) [APROPOS OF 2 OBSERVATIONS OF IMPORTED TRYPANOSOMIASIS]. Ann Med Nancy 46: 1432-1437.

569. Lathrop GD, Ominsky AJ (1965) Chagas' disease study in a group of individuals bitten by North American triatomids. Aeromed Rev 9: 1-5.

570. Lauricella MA, Stariolo RL, Riarte AR, Segura EL, Gurtler RE (2005) Distribution and pathogenicity of Trypanosoma cruzi isolated from peridomestic populations of Triatoma infestans and Triatoma guasayana from rural Western Argentina. Mem Inst Oswaldo Cruz 100: 123-129.

571. Le Bras M, Mazaudier E, Bigaignon-Receveur MC, Schmitt de la Brelie N, Becquart JP, et al. (1992) [Epidemiology and clinical aspects of imported tropical diseases]. Rev Med Interne 13: 205-210.

572. Leder K, Torresi J, Libman MD, Cramer JP, Castelli F, et al. GeoSentinel surveillance of illness in returned travelers, 2007-2011. Ann Intern Med 2013 Mar 19;158(6):456-68 doi: 107326/0003-4819-158-6-201303190-00005.

573. Lee BY, Bacon KM, Bottazzi ME, Hotez PJ Global economic burden of Chagas disease: a computational simulation model. Lancet Infect Dis 2013 Apr;13(4):342-8 doi: 101016/S1473-3099(13)70002-1 Epub 2013 Feb 8.

574. Lee BY, Bacon KM, Connor DL, Willig AM, Bailey RR The potential economic value of a Trypanosoma cruzi (Chagas disease) vaccine in Latin America. PLoS Negl Trop Dis 4: e916.

575. Lefebvre MF, Semalulu SS, Oatway AE, Nolan JW (1997) Trypanosomiasis in woodland caribou of northern Alberta. J Wildl Dis 33: 271-277.

576. Lefrancois T, Solano P, de la Rocque S, Bengaly Z, Reifenberg JM, et al. (1998) New epidemiological features on animal trypanosomiasis by molecular analysis in the pastoral zone of Sideradougou, Burkina Faso. Mol Ecol 7: 897-904.

577. Leiby DA, Rentas FJ, Nelson KE, Stambolis VA, Ness PM, et al. (2000) Evidence of Trypanosoma cruzi infection (Chagas' disease) among patients undergoing cardiac surgery. Circulation 102: 2978-2982.

578. Lejon V, Boelaert M, Jannin J, Moore A, Buscher P (2003) The challenge of Trypanosoma brucei gambiense sleeping sickness diagnosis outside Africa. Lancet Infect Dis 3: 804-808.

579. Lennox HA, Karcz DA, Tales H, El Masri M (2007) Chagas disease: clinical overview and implications for nursing. Medsurg Nurs 16: 229-235; quiz 236.

580. Leppert LL, Dufty AM, Jr., Stock S, Oleyar MD, Kaltenecker GS (2008) Survey of blood parasites in two forest owls, Northern Saw-whet Owls and Flammulated Owls, of western North America. J Wildl Dis 44: 475-479.

581. Lerdthusnee K, Nigro J, Monkanna T, Leepitakrat W, Leepitakrat S, et al. (2008) Surveys of rodent-borne disease in Thailand with a focus on scrub typhus assessment. Integr Zool 3: 267-273.

582. Leslie M Infectious diseases. A tropical disease hits the road. Science 333: 934.

583. Levine S, Fish D, Magnarelli LA, Anderson JF (1987) Choroid plexitis in white-tailed deer (Odocoileus virginianus) in southern New York State. Vet Pathol 24: 207-210.

584. Levy MZ, Bowman NM, Kawai V, Plotkin JB, Waller LA, et al. (2009) Spatial patterns in discordant diagnostic test results for Chagas disease: links to transmission hotspots. Clin Infect Dis 48: 1104-1106.

585. Li H, Tschudi C (2005) Novel and essential subunits in the 300-kilodalton nuclear cap binding complex of Trypanosoma brucei. Mol Cell Biol 25: 2216-2226.

586. Li S, Aksoy S (2000) A family of genes with growth factor and adenosine deaminase similarity are preferentially expressed in the salivary glands of Glossina m. morsitans. Gene 252: 83-93.

587. Li S, Kwon J, Aksoy S (2001) Characterization of genes expressed in the salivary glands of the tsetse fly, Glossina morsitans morsitans. Insect Mol Biol 10: 69-76.

588. Lima EC, Garcia I, Vicentelli MH, Vassalli P, Minoprio P (1997) Evidence for a protective role of tumor necrosis factor in the acute phase of Trypanosoma cruzi infection in mice. Infect Immun 65: 457-465.

589. Lima-Costa MF, Castro-Costa E, Uchoa E, Firmo J, Ribeiro AL, et al. (2009) A population-based study of the association between Trypanosoma cruzi infection and cognitive impairment in old age (the Bambui Study). Neuroepidemiology 32: 122-128.

590. Lindner AK, Priotto G The unknown risk of vertical transmission in sleeping sickness--a literature review. PLoS Negl Trop Dis 4: e783.

591. Linetzky B, Konfino J, Castellana N, De Maio F, Bahit MC, et al. Risk of cardiovascular events associated with positive serology for Chagas: a systematic review. Int J Epidemiol 2012 Oct;41(5):1356-66 doi: 101093/ije/dys125.

592. Linhares AC (2000) [Rotavirus infection in Brazil: epidemiology and challenges for its control]. Cad Saude Publica 16: 629-646.

593. Linhares AC, Bresee JS (2000) Rotavirus vaccines and vaccination in Latin America. Rev Panam Salud Publica 8: 305-331.

594. Liu Y, Mustafa M, Li HL, Nuortio L, Mustafa A, et al. (2000) Modulation of early immune responses and suppression of Trypanosoma brucei brucei infections by surgical denervation of the spleen. Neuroimmunomodulation 8: 31-38.

595. Lizundia R, Newman C, Buesching CD, Ngugi D, Blake D, et al. Evidence for a role of the host-specific flea (Paraceras melis) in the transmission of Trypanosoma (Megatrypanum) pestanai to the European badger. PLoS One 6: e16977.

596. Llenas-Garcia J, Hernando A, Fiorante S, Maseda D, Matarranz M, et al. Chagas disease screening among HIV-positive Latin American immigrants: an emerging problem. Eur J Clin Microbiol Infect Dis 2012 Aug;31(8):1991-7 doi: 101007/s10096-011-1531-4 Epub 2012 Jan 19.

597. Llewellyn MS, Miles MA, Carrasco HJ, Lewis MD, Yeo M, et al. (2009) Genome-scale multilocus microsatellite typing of Trypanosoma cruzi discrete typing unit I reveals phylogeographic structure and specific genotypes linked to human infection. PLoS Pathog 5: e1000410.

598. Lloyd-Smith JO, George D, Pepin KM, Pitzer VE, Pulliam JR, et al. (2009) Epidemic dynamics at the human-animal interface. Science 326: 1362-1367.

599. Lloyd-Smith JO, Poss M, Grenfell BT (2008) HIV-1/parasite co-infection and the emergence of new parasite strains. Parasitology 135: 795-806.

600. Loiseau PM, Gutierrez-Rios MT, De Frutos MI, Craciunescu DG (2001) Structure-activity relationships for new organometallic complexes active against bloodstream forms of Trypanosoma brucei brucei. Parasitol Res 87: 566-569.

601. Longoni SS, Lopez-Cespedes A, Sanchez-Moreno M, Bolio-Gonzalez ME, Sauri-Arceo CH, et al. Detection of different Leishmania spp. and Trypanosoma cruzi antibodies in cats from the Yucatan Peninsula (Mexico) using an iron superoxide dismutase excreted as antigen. Comp Immunol Microbiol Infect Dis 2012 Sep;35(5):469-76 doi: 101016/jcimid201204003 Epub 2012 May 5.

602. Longoni SS, Marin C, Sauri-Arceo CH, Lopez-Cespedes A, Rodriguez-Vivas RI, et al. An iron-superoxide dismutase antigen-based serological screening of dogs indicates their potential role in the transmission of cutaneous leishmaniasis and trypanosomiasis in Yucatan, Mexico. Vector Borne Zoonotic Dis 11: 815-821.

603. Lopes AH, McMahon-Pratt D (1989) Monoclonal antibodies specific for members of the genus Endotrypanum. J Protozool 36: 354-361.

604. Lopez-Cespedes A, Longoni SS, Sauri-Arceo CH, Rodriguez-Vivas RI, Villegas N, et al. Seroprevalence of antibodies against the excreted antigen superoxide dismutase by Trypanosoma cruzi in dogs from the Yucatan Peninsula (Mexico). Zoonoses Public Health 2013 Jun;60(4):277-83 doi: 101111/j1863-2378201201520x Epub 2012 Jul 10.

605. Lopez-Cespedes A, Longoni SS, Sauri-Arceo CH, Sanchez-Moreno M, Rodriguez-Vivas RI, et al. Leishmania spp. epidemiology of canine leishmaniasis in the Yucatan Peninsula. ScientificWorldJournal 2012;2012:945871 doi: 101100/2012/945871 Epub 2012 May 9.

606. Lopez-Cespedes A, Villagran E, Briceno Alvarez K, de Diego JA, Hernandez-Montiel HL, et al. Trypanosoma cruzi: seroprevalence detection in suburban population of Santiago de Queretaro (Mexico). ScientificWorldJournal 2012;2012:914129 doi: 101100/2012/914129 Epub 2012 May 3.

607. Lorca M, Soto F, Soto P, Padilla G, Nunez E, et al. (2008) [Chagas disease in the rural area of Metropolitan Region (Santiago) and V Region (Aconcagua), Chile]. Rev Med Chil 136: 945-946.

608. Loscher T, Nothdurft HD, Taelman H, Boogaerts M, Omar M, et al. (1989) [Sleeping sickness in German travelers to the tropics]. Dtsch Med Wochenschr 114: 1203-1206.

609. Louis FJ, Simarro PP (2005) [Rough start for the fight against sleeping sickness in French equatorial Africa]. Med Trop (Mars) 65: 251-257.

610. Lozano R, Naghavi M, Foreman K, Lim S, Shibuya K, et al. Global and regional mortality from 235 causes of death for 20 age groups in 1990 and 2010: a systematic analysis for the Global Burden of Disease Study 2010. Lancet 2012 Dec 15;380(9859):2095-128 doi: 101016/S0140-6736(12)61728-0.

611. Luna C, Bonizzoni M, Cheng Q, Robinson AS, Aksoy S, et al. (2001) Microsatellite polymorphism in tsetse flies (Diptera: Glossinidae). J Med Entomol 38: 376-381.

612. Luquetti AO, Ponce C, Ponce E, Esfandiari J, Schijman A, et al. (2003) Chagas' disease diagnosis: a multicentric evaluation of Chagas Stat-Pak, a rapid immunochromatographic assay with recombinant proteins of Trypanosoma cruzi. Diagn Microbiol Infect Dis 46: 265-271.

613. MacGregor P, Matthews KR New discoveries in the transmission biology of sleeping sickness parasites: applying the basics. J Mol Med (Berl) 88: 865-871.

614. Machado FS, Dutra WO, Esper L, Gollob KJ, Teixeira MM, et al. Current understanding of immunity to Trypanosoma cruzi infection and pathogenesis of Chagas disease. Semin Immunopathol 2012 Nov;34(6):753-70 doi: 101007/s00281-012-0351-7 Epub 2012 Oct 18.

615. MacLean LM, Odiit M, Chisi JE, Kennedy PG, Sternberg JM Focus-specific clinical profiles in human African Trypanosomiasis caused by Trypanosoma brucei rhodesiense. PLoS Negl Trop Dis 4: e906.

616. MacLeod A, Tweedie A, Welburn SC, Maudlin I, Turner CM, et al. (2000) Minisatellite marker analysis of Trypanosoma brucei: reconciliation of clonal, panmictic, and epidemic population genetic structures. Proc Natl Acad Sci U S A 97: 13442-13447.

617. Maguire JH, Hoff R, Sherlock I, Guimaraes AC, Sleigh AC, et al. (1987) Cardiac morbidity and mortality due to Chagas' disease: prospective electrocardiographic study of a Brazilian community. Circulation 75: 1140-1145.

618. Maguire JH, Hoff R, Sleigh AC, Mott KE, Ramos NB, et al. (1986) An outbreak of Chagas' disease in southwestern Bahia, Brazil. Am J Trop Med Hyg 35: 931-936.

619. Maguire JH, Mott KE, Hoff R, Guimaraes A, Franca JT, et al. (1982) A three-year follow-up study of infection with Trypanosoma cruzi and electrocardiographic abnormalities in a rural community in northeast Brazil. Am J Trop Med Hyg 31: 42-47.

620. Maguire JH, Mott KE, Lehman JS, Hoff R, Muniz TM, et al. (1983) Relationship of electrocardiographic abnormalities and seropositivity to Trypanosoma cruzi within a rural community in northeast Brazil. Am Heart J 105: 287-294.

621. Mahrt JL (1981) Seasonal prevalence of hematozoa in Willow Ptarmigan (Lagopus lagopus) from northwestern British Columbia. J Parasitol 67: 277-278.

622. Mahrt JL, Zwickel FC, Tessier TG (1991) Blood parasites of blue grouse (Dendragapus Obscurus) in western North America. J Wildl Dis 27: 482-485.

623. Maia da Silva F, Naiff RD, Marcili A, Gordo M, D'Affonseca Neto JA, et al. (2008) Infection rates and genotypes of Trypanosoma rangeli and T. cruzi infecting free-ranging Saguinus bicolor (Callitrichidae), a critically endangered primate of the Amazon Rainforest. Acta Trop 107: 168-173.

624. Majekodunmi AO, Fajinmi A, Dongkum C, Picozzi K, Thrusfield MV, et al. A longitudinal survey of African animal trypanosomiasis in domestic cattle on the Jos Plateau, Nigeria: prevalence, distribution and risk factors. Parasit Vectors 2013 Aug 19;6(1):239 doi: 101186/1756-3305-6-239.

625. Maloney J, Newsome A, Huang J, Kirby J, Kranz M, et al. Seroprevalence of Trypanosoma cruzi in raccoons from Tennessee. J Parasitol 96: 353-358.

626. Maltz MA, Weiss BL, O'Neill M, Wu Y, Aksoy S OmpA-mediated biofilm formation is essential for the commensal bacterium Sodalis glossinidius to colonize the tsetse fly gut. Appl Environ Microbiol 2012 Nov;78(21):7760-8 doi: 101128/AEM01858-12 Epub 2012 Aug 31.

627. Malvy D, Chappuis F Sleeping sickness. Clin Microbiol Infect 17: 986-995.

628. Malvy D, Djossou F, Weill FX, Chapuis P, Longy-Boursier M, et al. (2001) [Human African trypanosomiasis from Trypanosoma brucei gambiense with inoculation chancre in a French expatriate]. Med Trop (Mars) 61: 323-327.

629. Manne JM, Snively CS, Ramsey JM, Salgado MO, Barnighausen T, et al. Barriers to treatment access for Chagas disease in Mexico. PLoS Negl Trop Dis 2013 Oct 17;7(10):e2488 doi: 101371/journalpntd0002488.

630. Manner J, Heinicke F (2003) A model for left juxtaposition of the atrial appendages in the chick. Cardiol Young 13: 152-160.

631. Manner J, Seidl W, Heinicke F, Hesse H (2003) Teratogenic effects of suramin on the chick embryo. Anat Embryol (Berl) 206: 229-237.

632. Manzardo C, Trevino B, Gomez i Prat J, Cabezos J, Mongui E, et al. (2008) Communicable diseases in the immigrant population attended to in a tropical medicine unit: epidemiological aspects and public health issues. Travel Med Infect Dis 6: 4-11.

633. Marcet PL, Lehmann T, Groner G, Gurtler RE, Kitron U, et al. (2006) Identification and characterization of microsatellite markers in the Chagas disease vector Triatoma infestans (Heteroptera: Reduviidae). Infect Genet Evol 6: 32-37.

634. Marcet PL, Mora MS, Cutrera AP, Jones L, Gurtler RE, et al. (2008) Genetic structure of Triatoma infestans populations in rural communities of Santiago del Estero, northern Argentina. Infect Genet Evol 8: 835-846.

635. Maroli M, Jalouk L, Al Ahmed M, Bianchi R, Bongiorno G, et al. (2009) Aspects of the bionomics of Phlebotomus sergenti sandflies from an endemic area of anthroponotic cutaneous leishmaniasis in Aleppo Governorate, Syria. Med Vet Entomol 23: 148-154.

636. Maroli M, Mizzon V, Siragusa C, D'Oorazi A, Gradoni L (2001) Evidence for an impact on the incidence of canine leishmaniasis by the mass use of deltamethrin-impregnated dog collars in southern Italy. Med Vet Entomol 15: 358-363.

637. Marsden PD (1982) The treatment and control of parasitic diseases. Rev Infect Dis 4: 885-890.

638. Martin DL, Postan M, Lucas P, Gress R, Tarleton RL (2007) TGF-beta regulates pathology but not tissue CD8+ T cell dysfunction during experimental Trypanosoma cruzi infection. Eur J Immunol 37: 2764-2771.

639. Martinez-De la Puente J, Martinez J, Rivero-De-Aguilar J, Del Cerro S, Merino S Vector abundance determines Trypanosoma prevalence in nestling blue tits. Parasitology 2013 Jul;140(8):1009-15 doi: 101017/S0031182013000371 Epub 2013 Apr 18.

640. Martinez-Salio A, Calleja-Castano P, Valle-Arcos MD, Sanchez-Sanchez C, Diaz-Guzman J, et al. [Chagas disease and stroke code: an imported case]. Rev Neurol 53: 60-61.

641. Martins-Melo FR, Ramos AN, Jr., Alencar CH, Heukelbach J Prevalence of Chagas disease in Brazil: A systematic review and meta-analysis. Acta Trop 2013 Oct 15 pii: S0001-706X(13)00276-3 doi: 101016/jactatropica201310002.

642. Mas-Coma S, Bargues MD (2009) Populations, hybrids and the systematic concepts of species and subspecies in Chagas disease triatomine vectors inferred from nuclear ribosomal and mitochondrial DNA. Acta Trop 110: 112-136.

643. Masiga DK, Okech G, Irungu P, Ouma J, Wekesa S, et al. (2002) Growth and mortality in sheep and goats under high tsetse challenge in Kenya. Trop Anim Health Prod 34: 489-501.

644. Maslov DA, Westenberger SJ, Xu X, Campbell DA, Sturm NR (2007) Discovery and barcoding by analysis of spliced leader RNA gene sequences of new isolates of Trypanosomatidae from Heteroptera in Costa Rica and Ecuador. J Eukaryot Microbiol 54: 57-65.

645. Masumu J, Geysen D, Van den Bossche P (2009) Endemic type of animal trypanosomiasis is not associated with lower genotype variability of Trypanosoma congolense isolates circulating in livestock. Res Vet Sci 87: 265-269.

646. Mathers CD, Ezzati M, Lopez AD (2007) Measuring the burden of neglected tropical diseases: the global burden of disease framework. PLoS Negl Trop Dis 1: e114.

647. Mathieu-Daude F, Tibayrenc M (1994) Isozyme variability of Trypanosoma brucei s.l.: genetic, taxonomic, and epidemiological significance. Exp Parasitol 78: 1-19.

648. Matthews DM, Kingston N, Maki L, Nelms G (1979) Trypanosoma theileri Laveran, 1902, in Wyoming cattle. Am J Vet Res 40: 623-629.

649. Maudlin I, Welburn SC, Mehlitz D (1990) The relationship between rickettsia-like-organisms and trypanosome infections in natural populations of tsetse in Liberia. Trop Med Parasitol 41: 265-267.

650. Mayer DA, Fried B (2002) Aspects of human parasites in which surgical intervention may be important. Adv Parasitol 51: 1-94.

651. Mayer JP, Biancardi M, Altcheh J, Freilij H, Weinke T, et al. Congenital infections with Trypanosoma cruzi or Toxoplasma gondii are associated with decreased serum concentrations of interferon-gamma and interleukin-18 but increased concentrations of interleukin-10. Ann Trop Med Parasitol 104: 485-492.

652. McCarthy AE, Weld LH, Barnett ED, So H, Coyle C, et al. Spectrum of illness in international migrants seen at GeoSentinel clinics in 1997-2009, part 2: migrants resettled internationally and evaluated for specific health concerns. Clin Infect Dis 2013 Apr;56(7):925-33 doi: 101093/cid/cis1016 Epub 2012 Dec 7.

653. McCarthy M (2003) American Red Cross to screen blood for Chagas' disease. Lancet 362: 1988.

654. McKown RD, Upton SJ, Klemm RD, Ridley RK (1990) New host and locality record for Trypanosoma peromysci. J Parasitol 76: 281-283.

655. McMahon-Pratt D, Traub-Cseko Y, Lohman KL, Rogers DD, Beverley SM (1992) Loss of the GP46/M-2 surface membrane glycoprotein gene family in the Leishmania braziliensis complex. Mol Biochem Parasitol 50: 151-160.

656. McPhatter L, Roachell W, Mahmood F, Hoffman L, Lockwood N, et al. Vector surveillance to determine species composition and occurrence of trypanosoma cruzi at three military installations in San Antonio, Texas. US Army Med Dep J 2012 Jul-Sep:12-21.

657. Medei E, Pedrosa RC, Benchimol Barbosa PR, Costa PC, Hernandez CC, et al. (2007) Human antibodies with muscarinic activity modulate ventricular repolarization: basis for electrical disturbance. Int J Cardiol 115: 373-380.

658. Medina FA, Cohen AW, de Almeida CJ, Nagajyothi F, Braunstein VL, et al. (2007) Immune dysfunction in caveolin-1 null mice following infection with Trypanosoma cruzi (Tulahuen strain). Microbes Infect 9: 325-333.

659. Medina JW, de Escobar DM, de Vizcaino MA, Sanchez OG, Ballas SK (1987) Characteristics of a donor population in western Venezuela. Transfusion 27: 488-490.

660. Mendez GF, Cowie MR (2001) The epidemiological features of heart failure in developing countries: a review of the literature. Int J Cardiol 80: 213-219.

661. Mendonca Melo M, Rasica M, van Thiel PP, Richter C, Kager PA, et al. (2002) [Three patients with African sleeping sickness following a visit to Tanzania]. Ned Tijdschr Geneeskd 146: 2552-2556.

662. Meneghelli UG, de Rezende JM, Troncon LE, Madrid N, de Moura ML (1998) [A missing name in the history of chagasic megaesophagus: Joseph Cooper Reinhardt (1809/10-1873)]. Arq Gastroenterol 35: 1-8.

663. Menu F, Ginoux M, Rajon E, Lazzari CR, Rabinovich JE Adaptive developmental delay in Chagas disease vectors: an evolutionary ecology approach. PLoS Negl Trop Dis 4: e691.

664. Merino S, Potti J, Fargallo JA (1997) Blood parasites of passerine birds from central Spain. J Wildl Dis 33: 638-641.

665. Merino S, Potti J, Moreno J (1996) Maternal effort mediates the prevalence of trypanosomes in the offspring of a passerine bird. Proc Natl Acad Sci U S A 93: 5726-5730.

666. Meurs KM, Anthony MA, Slater M, Miller MW (1998) Chronic Trypanosoma cruzi infection in dogs: 11 cases (1987-1996). J Am Vet Med Assoc 213: 497-500.

667. Mhlanga JD (1996) Sleeping sickness: perspectives in African trypanosomiasis. Sci Prog 79 ( Pt 3): 183-214.

668. Michel JF, Dray S, de La Rocque S, Desquesnes M, Solano P, et al. (2002) Modelling bovine trypanosomosis spatial distribution by GIS in an agro-pastoral zone of Burkina Faso. Prev Vet Med 56: 5-18.

669. Michot TC, Garvin MC, Weidner EH (1995) Survey for blood parasites in redheads (Aythya americana) wintering at the Chandeleur Islands, Louisiana. J Wildl Dis 31: 90-92.

670. Migchelsen SJ, Buscher P, Hoepelman AI, Schallig HD, Adams ER Human African trypanosomiasis: a review of non-endemic cases in the past 20 years. Int J Infect Dis 15: e517-524.

671. Milei J, Mautner B, Storino R, Sanchez JA, Ferrans VJ (1992) Does Chagas' disease exist as an undiagnosed form of cardiomyopathy in the United States? Am Heart J 123: 1732-1735.

672. Miles MA, Llewellyn MS, Lewis MD, Yeo M, Baleela R, et al. (2009) The molecular epidemiology and phylogeography of Trypanosoma cruzi and parallel research on Leishmania: looking back and to the future. Parasitology 136: 1509-1528.

673. Milord F, Pepin J, Loko L, Ethier L, Mpia B (1992) Efficacy and toxicity of eflornithine for treatment of Trypanosoma brucei gambiense sleeping sickness. Lancet 340: 652-655.

674. Minneman RM, Hennink MM, Nicholls A, Salek SS, Palomeque FS, et al. Barriers to Testing and Treatment for Chagas Disease among Latino Immigrants in Georgia. J Parasitol Res 2012;2012:295034 doi: 101155/2012/295034 Epub 2012 Dec 30.

675. Miro JM, Blanes M, Norman F, Martin-Davila P Infections in solid organ transplantation in special situations: HIV-infection and immigration. Enferm Infecc Microbiol Clin 2012 Mar;30 Suppl 2:76-85 doi: 101016/S0213-005X(12)70086-1.

676. Moffitt JE, Venarske D, Goddard J, Yates AB, deShazo RD (2003) Allergic reactions to Triatoma bites. Ann Allergy Asthma Immunol 91: 122-128; quiz 128-130, 194.

677. Mohr W (1978) [Imported protozoan and worm infections]. Med Klin 73: 1598-1602.

678. Molina JM, Ruiz A, Juste MC, Corbera JA, Amador R, et al. (1999) Seroprevalence of Trypanosoma evansi in dromedaries (Camelus dromedarius) from the Canary Islands (Spain) using an antibody Ab-ELISA. Prev Vet Med 47: 53-59.

679. Moller AP, Nielsen JT (2007) Malaria and risk of predation: a comparative study of birds. Ecology 88: 871-881.

680. Molyneux D, Ndung'u J, Maudlin I Controlling sleeping sickness--"when will they ever learn?". PLoS Negl Trop Dis 4: e609.

681. Moncayo A Carlos Chagas: biographical sketch. Acta Trop 115: 1-4.

682. Moncayo A (1999) Progress towards interruption of transmission of Chagas disease. Mem Inst Oswaldo Cruz 94 Suppl 1: 401-404.

683. Moon TD, Oberhelman RA (2005) Antiparasitic therapy in children. Pediatr Clin North Am 52: 917-948, viii.

684. Moore DA, Edwards M, Escombe R, Agranoff D, Bailey JW, et al. (2002) African trypanosomiasis in travelers returning to the United Kingdom. Emerg Infect Dis 8: 74-76.

685. Moraes-Souza H, Bordin JO, Bardossy L, Blajchman MA (1996) Treatment of T. cruzi infected human platelet concentrates with aminomethyltrimethyl psoralen (AMT) and ultraviolet A (UV-A) light: preliminary results. Rev Soc Bras Med Trop 29: 47-49.

686. Moreno SN (1988) Metabolism and mode of action of gentian violet. Mem Inst Oswaldo Cruz 83 Suppl 1: 308-320.

687. Moretti G (1969) [African trypanosomiasis detected in France. Difficulties of diagnosis]. Presse Med 77: 1404.

688. Morlais I, Grebaut P, Bodo JM, Djoha S, Cuny G, et al. (1998) Detection and identification of trypanosomes by polymerase chain reaction in wild tsetse flies in Cameroon. Acta Trop 70: 109-117.

689. Morris SA, Weiss LM, Factor S, Bilezikian JP, Tanowitz H, et al. (1989) Verapamil ameliorates clinical, pathologic and biochemical manifestations of experimental chagasic cardiomyopathy in mice. J Am Coll Cardiol 14: 782-789.

690. Morrison LJ Parasite-driven pathogenesis in Trypanosoma brucei infections. Parasite Immunol 33: 448-455.

691. Morrison LJ, Tait A, McCormack G, Sweeney L, Black A, et al. (2008) Trypanosoma brucei gambiense Type 1 populations from human patients are clonal and display geographical genetic differentiation. Infect Genet Evol 8: 847-854.

692. Morrison LJ, Tweedie A, Black A, Pinchbeck GL, Christley RM, et al. (2009) Discovery of mating in the major African livestock pathogen Trypanosoma congolense. PLoS One 4: e5564.

693. Morsy TA, Schnur LF, Feinsod FM, Michael SA, Saah A, et al. (1988) The discovery and preliminary characterization of a novel trypanosomatid parasite from Rattus norvegicus and stray dogs from Alexandria, Egypt. Ann Trop Med Parasitol 82: 437-444.

694. Moser DR, Cook GA, Ochs DE, Bailey CP, McKane MR, et al. (1989) Detection of Trypanosoma congolense and Trypanosoma brucei subspecies by DNA amplification using the polymerase chain reaction. Parasitology 99 Pt 1: 57-66.

695. Moser DR, Kirchhoff LV, Donelson JE (1989) Detection of Trypanosoma cruzi by DNA amplification using the polymerase chain reaction. J Clin Microbiol 27: 1477-1482.

696. Mota E, Todd CW, Maguire JH, Portugal D, Santana O, et al. (1984) Megaesophagus and seroreactivity to Trypanosoma cruzi in a rural community in northeast Brazil. Am J Trop Med Hyg 33: 820-826.

697. Mota EA, Guimaraes AC, Santana OO, Sherlock I, Hoff R, et al. (1990) A nine year prospective study of Chagas' disease in a defined rural population in northeast Brazil. Am J Trop Med Hyg 42: 429-440.

698. Mott KE, Desjeux P, Moncayo A, Ranque P, de Raadt P (1990) Parasitic diseases and urban development. Bull World Health Organ 68: 691-698.

699. Mott KE, Nuttall I, Desjeux P, Cattand P (1995) New geographical approaches to control of some parasitic zoonoses. Bull World Health Organ 73: 247-257.

700. Mountain JC (1968) Chagas' disease. Proc R Soc Med 61: 444-445.

701. Muller E, Gargani D, Banuls AL, Tibayrenc M, Dollet M (1997) Classification of plant trypanosomatids (Phytomonas spp.): parity between random-primer DNA typing and multilocus enzyme electrophoresis. Parasitology 115 ( Pt 4): 403-409.

702. Muller G, Grebaut P, Gouteux JP (2004) An agent-based model of sleeping sickness: simulation trials of a forest focus in southern Cameroon. C R Biol 327: 1-11.

703. Mumba D, Bohorquez E, Messina J, Kande V, Taylor SM, et al. Prevalence of human African trypanosomiasis in the Democratic Republic of the Congo. PLoS Negl Trop Dis 5: e1246.

704. Mungube EO, Diall O, Baumann MP, Hoppenheit A, Hinney B, et al. Best-bet integrated strategies for containing drug-resistant trypanosomes in cattle. Parasit Vectors 2012 Aug 8;5:164 doi: 101186/1756-3305-5-164.

705. Mungube EO, Vitouley HS, Allegye-Cudjoe E, Diall O, Boucoum Z, et al. Detection of multiple drug-resistant Trypanosoma congolense populations in village cattle of south-east Mali. Parasit Vectors 2012 Aug 1;5:155 doi: 101186/1756-3305-5-155.

706. Muniz Mde M, Morais ESTP, Meyer W, Nosanchuk JD, Zancope-Oliveira RM Comparison of different DNA-based methods for molecular typing of Histoplasma capsulatum. Appl Environ Microbiol 76: 4438-4447.

707. Munoz E, Ferrer D, Molina R, Adlard RD (1999) Prevalence of haematozoa in birds of prey in Catalonia, north-east Spain. Vet Rec 144: 632-636.

708. Murthy S, Keystone J, Kissoon N Infections of the developing world. Crit Care Clin 2013 Jul;29(3):485-507 doi: 101016/jccc201303005.

709. Mwangi EK, Stevenson P, Ndung UJ, Stear MJ, Reid SW, et al. (1998) Studies on host resistance to tick infestations among trypanotolerant Bos indicus cattle breeds in east Africa. Ann N Y Acad Sci 849: 195-208.

710. Nadjm B, Van Tulleken C, Macdonald D, Chiodini PL (2009) East African trypanosomiasis in a pregnant traveler. Emerg Infect Dis 15: 1866-1867.

711. Nagajyothi F, Zhao D, Machado FS, Weiss LM, Schwartz GJ, et al. Crucial role of the central leptin receptor in murine Trypanosoma cruzi (Brazil strain) infection. J Infect Dis 202: 1104-1113.

712. Nagajyothi F, Zhao D, Weiss LM, Tanowitz HB Curcumin treatment provides protection against Trypanosoma cruzi infection. Parasitol Res 2012 Jun;110(6):2491-9 doi: 101007/s00436-011-2790-9 Epub 2012 Jan 4.

713. Nagarkatti R, Bist V, Sun S, Fortes de Araujo F, Nakhasi HL, et al. Development of an aptamer-based concentration method for the detection of Trypanosoma cruzi in blood. PLoS One 2012;7(8):e43533 doi: 101371/journalpone0043533 Epub 2012 Aug 22.

714. Navin TR, Roberto RR, Juranek DD, Limpakarnjanarat K, Mortenson EW, et al. (1985) Human and sylvatic Trypanosoma cruzi infection in California. Am J Public Health 75: 366-369.

715. Nayduch D, Aksoy S (2007) Refractoriness in tsetse flies (Diptera: Glossinidae) may be a matter of timing. J Med Entomol 44: 660-665.

716. Ndao M, Rainczuk A, Rioux MC, Spithill TW, Ward BJ Is SELDI-TOF a valid tool for diagnostic biomarkers? Trends Parasitol 26: 561-567.

717. Ndung'u JM, Bieler S, Roscigno G "Piggy-backing" on diagnostic platforms brings hope to neglected diseases: the case of sleeping sickness. PLoS Negl Trop Dis 4: e715.

718. Nelder MP, Reeves WK, Adler PH, Wozniak A, Wills W (2009) Ectoparasites and associated pathogens of free-roaming and captive animals in zoos of South Carolina. Vector Borne Zoonotic Dis 9: 469-477.

719. Neves SF, Eloi-Santos S, Ramos R, Rigueirinho S, Gazzinelli G, et al. (1999) In utero sensitization in Chagas' disease leads to altered lymphocyte phenotypic patterns in the newborn cord blood mononuclear cells. Parasite Immunol 21: 631-639.

720. Nickerson P, Orr P, Schroeder ML, Sekla L, Johnston JB (1989) Transfusion-associated Trypanosoma cruzi infection in a non-endemic area. Ann Intern Med 111: 851-853.

721. Nieto PD, Boughton R, Dorn PL, Steurer F, Raychaudhuri S, et al. (2009) Comparison of two immunochromatographic assays and the indirect immunofluorescence antibody test for diagnosis of Trypanosoma cruzi infection in dogs in south central Louisiana. Vet Parasitol 165: 241-247.

722. Nishioka S, Gyorkos TW (2001) Tattoos as risk factors for transfusion-transmitted diseases. Int J Infect Dis 5: 27-34.

723. Noireau F, Lemesre JL, Vervoort T (1991) Absence of serological markers of infection with Trypanosoma brucei gambiense in domestic animals in a sleeping sickness focus in south Congo. Trop Med Parasitol 42: 195-196.

724. Norman FF, Perez de Ayala A, Perez-Molina JA, Monge-Maillo B, Zamarron P, et al. Neglected tropical diseases outside the tropics. PLoS Negl Trop Dis 4: e762.

725. Norris A, Galea G (2001) The impact of the new tick-box questionnaire, and the personal donor interview, on donor deferrals in the East of Scotland. Transfus Med 11: 183-187.

726. Nouvellet P, Dumonteil E, Gourbiere S The Improbable Transmission of Trypanosoma cruzi to Human: The Missing Link in the Dynamics and Control of Chagas Disease. PLoS Negl Trop Dis 2013 Nov 7;7(11):e2505 doi: 101371/journalpntd0002505.

727. Nunes MR, Travassos da Rosa AP, Weaver SC, Tesh RB, Vasconcelos PF (2005) Molecular epidemiology of group C viruses (Bunyaviridae, Orthobunyavirus) isolated in the Americas. J Virol 79: 10561-10570.

728. Ocana-Mayorga S, Llewellyn MS, Costales JA, Miles MA, Grijalva MJ Sex, subdivision, and domestic dispersal of Trypanosoma cruzi lineage I in southern Ecuador. PLoS Negl Trop Dis 4: e915.

729. Older JJ (1969) Clinical study of Chagas' disease (trypanosomiasis cruzi). South Med J 62: 729-733.

730. Oliveira BG, Velasquez-Melendez G, Rincon LG, Ciconelli RM, Sousa LA, et al. (2008) Health-related quality of life in Brazilian pacemaker patients. Pacing Clin Electrophysiol 31: 1178-1183.

731. Oliveira FL, Duczmal LH, Cancado AL, Tavares R Nonparametric intensity bounds for the delineation of spatial clusters. Int J Health Geogr 10: 1.

732. Oliveira I, Torrico F, Munoz J, Gascon J Congenital transmission of Chagas disease: a clinical approach. Expert Rev Anti Infect Ther 8: 945-956.

733. Oliveira-Filho J, Viana LC, Vieira-de-Melo RM, Faical F, Torreao JA, et al. (2005) Chagas disease is an independent risk factor for stroke: baseline characteristics of a Chagas Disease cohort. Stroke 36: 2015-2017.

734. Ollivier G, Legros D (2001) [Human african trypanosomiasis: A history of its therapies and their failures]. Trop Med Int Health 6: 855-863.

735. Olsen PF (1966) Epizoology of Chagas' disease in the southeastern United States. Wildl Dis 47: Suppl:1-108.

736. Orecchia P, Bianchini M, Catalini N, Cataudella S, Paggi L (1987) Parasitological study of a population of Tiber River eels (Anguilla anguilla). Parassitologia 29: 37-47.

737. Orozco MM, Enriquez GF, Alvarado-Otegui JA, Cardinal MV, Schijman AG, et al. New sylvatic hosts of Trypanosoma cruzi and their reservoir competence in the humid Chaco of Argentina: a longitudinal study. Am J Trop Med Hyg 2013 May;88(5):872-82 doi: 104269/ajtmh12-0519 Epub 2013 Mar 25.

738. Ouaissi A, Guilvard E, Delneste Y, Caron G, Magistrelli G, et al. (2002) The Trypanosoma cruzi Tc52-released protein induces human dendritic cell maturation, signals via Toll-like receptor 2, and confers protection against lethal infection. J Immunol 168: 6366-6374.

739. Overath P, Haag J, Mameza MG, Lischke A (1999) Freshwater fish trypanosomes: definition of two types, host control by antibodies and lack of antigenic variation. Parasitology 119 ( Pt 6): 591-601.

740. Paige CF, Scholl DT, Truman RW (2002) Prevalence and incidence density of Mycobacterium leprae and Trypanosoma cruzi infections within a population of wild nine-banded armadillos. Am J Trop Med Hyg 67: 528-532.

741. Pais R, Lohs C, Wu Y, Wang J, Aksoy S (2008) The obligate mutualist Wigglesworthia glossinidia influences reproduction, digestion, and immunity processes of its host, the tsetse fly. Appl Environ Microbiol 74: 5965-5974.

742. Pan AA, McMahon-Pratt D (1988) Monoclonal antibodies specific for the amastigote stage of Leishmania pifanoi. I. Characterization of antigens associated with stage- and species-specific determinants. J Immunol 140: 2406-2414.

743. Pan AA, McMahon-Pratt D (1989) Amastigote and epimastigote stage-specific components of Trypanosoma cruzi characterized by using monoclonal antibodies. Purification and molecular characterization of an 83-kilodalton amastigote protein. J Immunol 143: 1001-1008.

744. Parija SC, Giri S Emerging protozoal pathogens in India: How prepared are we to face the threat? Trop Parasitol 2012 Jan;2(1):13-9 doi: 104103/2229-507097233.

745. Pascucci I, Di Provvido A, Camma C, Di Francesco G, Calistri P, et al. Diagnosis of dourine in outbreaks in Italy. Vet Parasitol 2013 Mar 31;193(1-3):30-8 doi: 101016/jvetpar201212006 Epub 2012 Dec 20.

746. Patel JM, Rosypal AC, Zimmerman KL, Monroe WE, Sriranganathan N, et al. Isolation, mouse pathogenicity, and genotyping of Trypanosoma cruzi from an English Cocker Spaniel from Virginia, USA. Vet Parasitol 2012 Jul 6;187(3-4):394-8 doi: 101016/jvetpar201201031 Epub 2012 Jan 28.

747. Patnaik MM, Stauffer WM, Campagna A, Stauffer CI, Walker PF (2007) When the market fails, the poor pay. Minn Med 90: 38-40.

748. Patrick KL, Luz PM, Ruan JP, Shi H, Ullu E, et al. (2008) Genomic rearrangements and transcriptional analysis of the spliced leader-associated retrotransposon in RNA interference-deficient Trypanosoma brucei. Mol Microbiol 67: 435-447.

749. Patrick KL, Shi H, Kolev NG, Ersfeld K, Tschudi C, et al. (2009) Distinct and overlapping roles for two Dicer-like proteins in the RNA interference pathways of the ancient eukaryote Trypanosoma brucei. Proc Natl Acad Sci U S A 106: 17933-17938.

750. Patterson JS, Barbosa SE, Feliciangeli MD (2009) On the genus Panstrongylus Berg 1879: evolution, ecology and epidemiological significance. Acta Trop 110: 187-199.

751. Pawelczyk A, Bajer A, Behnke JM, Gilbert FS, Sinski E (2004) Factors affecting the component community structure of haemoparasites in common voles ( Microtus arvalis) from the Mazury Lake District region of Poland. Parasitol Res 92: 270-284.

752. Pays J-F [Chagas disease: when to think of it in France?]. Rev Prat 2013 Sep;63(7):913-8.

753. Pays JF, Saliou P (2005) A comparative approach to the French medical missions in Brazil and in sub-Saharan Africa before the Second World War. Parassitologia 47: 361-368.

754. Peirce MA, Neal C (1974) Trypanosoma (Megatrypanum) pestanai in British badgers (Meles meles). Int J Parasitol 4: 439-440.

755. Pelosse P, Kribs-Zaleta CM, Ginoux M, Rabinovich JE, Gourbiere S, et al. Influence of vectors' risk-spreading strategies and environmental stochasticity on the epidemiology and evolution of vector-borne diseases: the example of Chagas' disease. PLoS One 2013;8(8):e70830 doi: 101371/journalpone0070830 eCollection 2013.

756. Penchenier L, Mathleu-Daude F, Brengues C, Banuls AL, Tibayrenc M (1997) Population structure of Trypanosoma brucei S. L. in Cote d'Ivoire assayed by multilocus enzyme electrophoresis: epidemiological and taxonomical considerations. J Parasitol 83: 19-22.

757. Pepin J, Guern C, Milord F, Ethier L, Bokelo M, et al. (1989) [The use of difluoromethylornithine in congenital trypanosomiasis due to Trypanosoma brucei-gambiense]. Med Trop (Mars) 49: 83-85.

758. Pepin J, Khonde N, Maiso F, Doua F, Jaffar S, et al. (2000) Short-course eflornithine in Gambian trypanosomiasis: a multicentre randomized controlled trial. Bull World Health Organ 78: 1284-1295.

759. Pepin J, Labbe AC (2008) Noble goals, unforeseen consequences: control of tropical diseases in colonial Central Africa and the iatrogenic transmission of blood-borne viruses. Trop Med Int Health 13: 744-753.

760. Pepin J, Labbe AC, Mamadou-Yaya F, Mbelesso P, Mbadingai S, et al. Iatrogenic transmission of human T cell lymphotropic virus type 1 and hepatitis C virus through parenteral treatment and chemoprophylaxis of sleeping sickness in colonial Equatorial Africa. Clin Infect Dis 51: 777-784.

761. Pepin J, Meda HA (2001) The epidemiology and control of human African trypanosomiasis. Adv Parasitol 49: 71-132.

762. Pepin J, Milord F, Guern C, Schechter PJ (1987) Difluoromethylornithine for arseno-resistant Trypanosoma brucei gambiense sleeping sickness. Lancet 2: 1431-1433.

763. Pepin J, Milord F, Khonde AN, Niyonsenga T, Loko L, et al. (1995) Risk factors for encephalopathy and mortality during melarsoprol treatment of Trypanosoma brucei gambiense sleeping sickness. Trans R Soc Trop Med Hyg 89: 92-97.

764. Pepin J, Mpia B, Iloasebe M (2002) Trypanosoma brucei gambiense African trypanosomiasis: differences between men and women in severity of disease and response to treatment. Trans R Soc Trop Med Hyg 96: 421-426.

765. Pepin J, Plamondon M, Alves AC, Beaudet M, Labbe AC (2006) Parenteral transmission during excision and treatment of tuberculosis and trypanosomiasis may be responsible for the HIV-2 epidemic in Guinea-Bissau. AIDS 20: 1303-1311.

766. Pereira BI, Nazareth C, Malcata L, Alves H, Fernandez JR, et al. [Transfusion-transmitted protozoal infections: what is the risk in non-endemic countries?]. Acta Med Port 2011 Dec;24 Suppl 4:897-906 Epub 2011 Dec 31.

767. Perera DR, Donovan DL, Stroud GM, Schultz MG (1969) Imported African sleeping sickness. JAMA 209: 270.

768. Perez-Ramirez L, Barnabe C, Sartori AM, Ferreira MS, Tolezano JE, et al. (1999) Clinical analysis and parasite genetic diversity in human immunodeficiency virus/Chagas' disease coinfections in Brazil. Am J Trop Med Hyg 61: 198-206.

769. Perleth M (1997) The discovery of Chagas' disease and the formation of the early Chagas' disease concept. Hist Philos Life Sci 19: 211-236.

770. Perret JL, Kombila M, Pemba LF (1997) [Gabon: panoply syndrome]. Med Trop (Mars) 57: 337-342.

771. Petana WB (1978) American trypanosomiasis (Chagas' disease) in the Caribbean. Bull Pan Am Health Organ 12: 45-50.

772. Petherick A Campaigning for Chagas disease. Nature 465: S21-22.

773. Petru AM, Azimi PH, Cummins SK, Sjoerdsma A (1988) African sleeping sickness in the United States. Successful treatment with eflornithine. Am J Dis Child 142: 224-228.

774. Phalen DN, Taylor C, Phalen SW, Bennett GF (1995) Hemograms and hematozoa of sharp-shinned (Accipiter striatus) and Cooper's hawks (Accipiter cooperii) captured during spring migration in northern New York. J Wildl Dis 31: 216-222.

775. Piacenza L, Peluffo G, Alvarez MN, Kelly JM, Wilkinson SR, et al. (2008) Peroxiredoxins play a major role in protecting Trypanosoma cruzi against macrophage- and endogenously-derived peroxynitrite. Biochem J 410: 359-368.

776. Piacenza L, Peluffo G, Alvarez MN, Martinez A, Radi R Trypanosoma cruzi antioxidant enzymes as virulence factors in Chagas disease. Antioxid Redox Signal 2013 Sep 1;19(7):723-34 doi: 101089/ars20124618 Epub 2012 May 21.

777. Piccinali RV, Canale DM, Sandoval AE, Cardinal MV, Jensen O, et al. Triatoma infestans bugs in Southern Patagonia, Argentina. Emerg Infect Dis 16: 887-889.

778. Piesman J, Mota E, Sherlock IA, Todd CW (1985) Trypanosoma cruzi: association between seroreactivity of children and infection rates in domestic Panstrongylus megistus (Hemiptera: Reduviidae). J Med Entomol 22: 130-133.

779. Piesman J, Sherlock IA, Mota E, Todd CW, Hoff R, et al. (1985) Association between household triatomine density and incidence of Trypanosoma cruzi infection during a nine-year study in Castro Alves, Bahia, Brazil. Am J Trop Med Hyg 34: 866-869.

780. Pietrzak SM, Pung OJ (1998) Trypanosomiasis in raccoons from Georgia. J Wildl Dis 34: 132-136.

781. Pinazo MJ, Canas E, Elizalde JI, Garcia M, Gascon J, et al. Diagnosis, management and treatment of chronic Chagas' gastrointestinal disease in areas where Trypanosoma cruzi infection is not endemic. Gastroenterol Hepatol 33: 191-200.

782. Pinazo MJ, Ignacio Elizalde J, de Jesus Posada E, Gascon J Co-infection with two emergent old pathogens: Trypanosoma cruzi and Helicobacter pylori. Enferm Infecc Microbiol Clin 28: 751-752.

783. Pinchbeck GL, Morrison LJ, Tait A, Langford J, Meehan L, et al. (2008) Trypanosomosis in The Gambia: prevalence in working horses and donkeys detected by whole genome amplification and PCR, and evidence for interactions between trypanosome species. BMC Vet Res 4: 7.

784. Pinto CM, Baxter BD, Hanson JD, Mendez-Harclerode FM, Suchecki JR, et al. Using museum collections to detect pathogens. Emerg Infect Dis 16: 356-357.

785. Piron M, Fisa R, Casamitjana N, Lopez-Chejade P, Puig L, et al. (2007) Development of a real-time PCR assay for Trypanosoma cruzi detection in blood samples. Acta Trop 103: 195-200.

786. Pisharath H, Zao CL, Kreeger J, Portugal S, Kawabe T, et al. Immunopathologic characterization of naturally acquired Trypanosoma cruzi infection and cardiac sequalae in cynomolgus macaques (Macaca fascicularis). J Am Assoc Lab Anim Sci 2013 Sep;52(5):545-52.

787. Pizzini CV, Zancope-Oliveira RM, Reiss E, Hajjeh R, Kaufman L, et al. (1999) Evaluation of a western blot test in an outbreak of acute pulmonary histoplasmosis. Clin Diagn Lab Immunol 6: 20-23.

788. Pomper GJ, Wu Y, Snyder EL (2003) Risks of transfusion-transmitted infections: 2003. Curr Opin Hematol 10: 412-418.

789. Ponte-Sucre A, Moll H (2005) Parasites, flies and men--21st Meeting of the German Society of Parasitology in Wurzburg. Eur J Cell Biol 84: 7-13.

790. Popp M, Erler S, Lattorff HM Seasonal variability of prevalence and occurrence of multiple infections shape the population structure of Crithidia bombi, an intestinal parasite of bumblebees (Bombus spp.). Microbiologyopen 2012 Dec;1(4):362-72 doi: 101002/mbo335 Epub 2012 Sep 23.

791. Povoa MM, Conn JE, Schlichting CD, Amaral JC, Segura MN, et al. (2003) Malaria vectors, epidemiology, and the re-emergence of Anopheles darlingi in Belem, Para, Brazil. J Med Entomol 40: 379-386.

792. Priotto G, Kasparian S, Mutombo W, Ngouama D, Ghorashian S, et al. (2009) Nifurtimox-eflornithine combination therapy for second-stage African Trypanosoma brucei gambiense trypanosomiasis: a multicentre, randomised, phase III, non-inferiority trial. Lancet 374: 56-64.

793. Provost A, Bezuidenhout JD (1987) The historical background and global importance of heartwater. Onderstepoort J Vet Res 54: 165-169.

794. Pruvot M, Kamyingkird K, Desquesnes M, Sarataphan N, Jittapalapong S A comparison of six primer sets for detection of Trypanosoma evansi by polymerase chain reaction in rodents and Thai livestock. Vet Parasitol 171: 185-193.

795. Pung OJ, Banks CW, Jones DN, Krissinger MW (1995) Trypanosoma cruzi in wild raccoons, opossums, and triatomine bugs in southeast Georgia, U.S.A. J Parasitol 81: 324-326.

796. Pung OJ, Spratt J, Clark CG, Norton TM, Carter J (1998) Trypanosoma cruzi infection of free-ranging lion-tailed macaques (Macaca silenus) and ring-tailed lemurs (Lemur catta) on St. Catherine's Island, Georgia, USA. J Zoo Wildl Med 29: 25-30.

797. Punukollu G, Gowda RM, Khan IA, Navarro VS, Vasavada BC (2007) Clinical aspects of the Chagas' heart disease. Int J Cardiol 115: 279-283.

798. Qvarnstrom Y, Schijman AG, Veron V, Aznar C, Steurer F, et al. Sensitive and specific detection of Trypanosoma cruzi DNA in clinical specimens using a multi-target real-time PCR approach. PLoS Negl Trop Dis 2012;6(7):e1689 doi: 101371/journalpntd0001689 Epub 2012 Jul 3.

799. Rascalou G, Pontier D, Menu F, Gourbiere S Emergence and prevalence of human vector-borne diseases in sink vector populations. PLoS One 2012;7(5):e36858 doi: 101371/journalpone0036858 Epub 2012 May 18.

800. Rassi A, Jr., Rassi A, Little WC (2000) Chagas' heart disease. Clin Cardiol 23: 883-889.

801. Rathkolb B, Noyes HA, Brass A, Dark P, Fuchs H, et al. (2009) Clinical chemistry of congenic mice with quantitative trait loci for predicted responses to Trypanosoma congolense infection. Infect Immun 77: 3948-3957.

802. Ratmanov P, Mediannikov O, Raoult D Vectorborne diseases in West Africa: geographic distribution and geospatial characteristics. Trans R Soc Trop Med Hyg 2013 May;107(5):273-84 doi: 101093/trstmh/trt020 Epub 2013 Mar 10.

803. Raveenthiran V, Madiba TE, Atamanalp SS, De U Volvulus of the sigmoid colon. Colorectal Dis 12: e1-17.

804. Ravel S, de Meeus T, Dujardin JP, Zeze DG, Gooding RH, et al. (2007) The tsetse fly Glossina palpalis palpalis is composed of several genetically differentiated small populations in the sleeping sickness focus of Bonon, Cote d'Ivoire. Infect Genet Evol 7: 116-125.

805. Ravel S, Grebaut P, Mariani C, Jamonneau V, Cuisance D, et al. (2004) Monitoring the susceptibility of Glossina palpalis gambiensis and G. morsitans morsitans to experimental infection with savannah-type Trypanosoma congolense, using the polymerase chain reaction. Ann Trop Med Parasitol 98: 29-36.

806. Ravel S, Herve JP, Diarrassouba S, Kone A, Cuny G (2002) Microsatellite markers for population genetic studies in Aedes aegypti (Diptera: Culicidae) from Cote d'Ivoire: evidence for a microgeographic genetic differentiation of mosquitoes from Bouake. Acta Trop 82: 39-49.

807. Ravel S, Patrel D, Koffi M, Jamonneau V, Cuny G (2006) Cyclical transmission of Trypanosoma brucei gambiense in Glossina palpalis gambiensis displays great differences among field isolates. Acta Trop 100: 151-155.

808. Reddy HL, Doane SK, Keil SD, Marschner S, Goodrich RP Development of a riboflavin and ultraviolet light-based device to treat whole blood. Transfusion 2013 Jan;53 Suppl 1:131S-136S doi: 101111/trf12047.

809. Reed SG (1988) In vivo administration of recombinant IFN-gamma induces macrophage activation, and prevents acute disease, immune suppression, and death in experimental Trypanosoma cruzi infections. J Immunol 140: 4342-4347.

810. Reesink HW (2005) European strategies against the parasite transfusion risk. Transfus Clin Biol 12: 1-4.

811. Reglero M, Vicente J, Rouco C, Villafuerte R, Gortazar C (2007) Trypanosoma spp. infection in wild rabbits (Oryctolagus cuniculus) during a restocking program in Southern Spain. Vet Parasitol 149: 178-184.

812. Reguera RM, Redondo CM, Gutierrez de Prado R, Perez-Pertejo Y, Balana-Fouce R (2006) DNA topoisomerase I from parasitic protozoa: a potential target for chemotherapy. Biochim Biophys Acta 1759: 117-131.

813. Reincke M, Arlt W, Heppner C, Petzke F, Chrousos GP, et al. (1998) Neuroendocrine dysfunction in African trypanosomiasis. The role of cytokines. Ann N Y Acad Sci 840: 809-821.

814. Reis RS, Almeida-Paes R, Muniz Mde M, Tavares PM, Monteiro PC, et al. (2009) Molecular characterisation of Sporothrix schenckii isolates from humans and cats involved in the sporotrichosis epidemic in Rio de Janeiro, Brazil. Mem Inst Oswaldo Cruz 104: 769-774.

815. Reisenman CE, Gregory T, Guerenstein PG, Hildebrand JG Feeding and defecation behavior of Triatoma rubida (Uhler, 1894) (Hemiptera: Reduviidae) under laboratory conditions, and its potential role as a vector of Chagas disease in Arizona, USA. Am J Trop Med Hyg 85: 648-656.

816. Reithinger R, Tarleton RL, Urbina JA, Kitron U, Gurtler RE (2009) Eliminating Chagas disease: challenges and a roadmap. BMJ 338: b1283.

817. Remais JV, Xiao N, Akullian A, Qiu D, Blair D Genetic assignment methods for gaining insight into the management of infectious disease by understanding pathogen, vector, and host movement. PLoS Pathog 7: e1002013.

818. Remesar MC, Gamba C, Colaianni IF, Puppo M, Sartor PA, et al. (2009) Estimation of sensitivity and specificity of several Trypanosoma cruzi antibody assays in blood donors in Argentina. Transfusion 49: 2352-2358.

819. Remme JH, De Raadt P, Godal T (1993) The burden of tropical diseases. Med J Aust 158: 465-469.

820. Ribeiro AL, Cavalvanti PS, Lombardi F, Nunes Mdo C, Barros MV, et al. (2008) Prognostic value of signal-averaged electrocardiogram in Chagas disease. J Cardiovasc Electrophysiol 19: 502-509.

821. Ribeiro AL, Nunes MP, Teixeira MM, Rocha MO Diagnosis and management of Chagas disease and cardiomyopathy. Nat Rev Cardiol 2012 Oct;9(10):576-89 doi: 101038/nrcardio2012109 Epub 2012 Jul 31.

822. Ribeiro AL, Sabino EC, Marcolino MS, Salemi VM, Ianni BM, et al. Electrocardiographic abnormalities in Trypanosoma cruzi seropositive and seronegative former blood donors. PLoS Negl Trop Dis 2013;7(2):e2078 doi: 101371/journalpntd0002078 Epub 2013 Feb 28.

823. Richer W, Kengne P, Cortez MR, Perrineau MM, Cohuet A, et al. (2007) Active dispersal by wild Triatoma infestans in the Bolivian Andes. Trop Med Int Health 12: 759-764.

824. Richman TB, Kerdel FA (1989) Amebiasis and trypanosomiasis. Dermatol Clin 7: 301-311.

825. Rintamaki PT, Huhta E, Jokimaki J, Squires-Parsons D (1999) Leucocytozoonosis and trypanosomiasis in redstarts in Finland. J Wildl Dis 35: 603-607.

826. Rio RV, Wu YN, Filardo G, Aksoy S (2006) Dynamics of multiple symbiont density regulation during host development: tsetse fly and its microbial flora. Proc Biol Sci 273: 805-814.

827. Rioux JA, Albaret JL, Bres A, Dumas A (1966) [Presence of trypanosoma pestanai Bettencourt and Franca, 1905, in badgers from the south of France]. Ann Parasitol Hum Comp 41: 281-288.

828. Ripamonti D, Massari M, Arici C, Gabbi E, Farina C, et al. (2002) African sleeping sickness in tourists returning from Tanzania: the first 2 Italian cases from a small outbreak among European travelers. Clin Infect Dis 34: E18-22.

829. Rislakki V (1971) Studies on the prevelence and effect of Trypanosoma lewisi infection in Finnish rats. Acta Vet Scand 12: 448-450.

830. Rodrigues JC, Godinho JL, de Souza W Biology of human pathogenic trypanosomatids: epidemiology, lifecycle and ultrastructure. Subcell Biochem 2014;74:1-42 doi: 101007/978-94-007-7305-9_1.

831. Rodriguez NF, Tejedor-Junco MT, Gonzalez-Martin M, Santana del Pino A, Gutierrez C Cross-sectional study on prevalence of Trypanosoma evansi infection in domestic ruminants in an endemic area of the Canary Islands (Spain). Prev Vet Med 2012 Jun 1;105(1-2):144-8 doi: 101016/jprevetmed201202006 Epub 2012 Mar 3.

832. Rodriguez NF, Tejedor-Junco MT, Hernandez-Trujillo Y, Gonzalez M, Gutierrez C The role of wild rodents in the transmission of Trypanosoma evansi infection in an endemic area of the Canary Islands (Spain). Vet Parasitol 174: 323-327.

833. Rodriguez-Gonzalez I, Marin C, Hitos AB, Rosales MJ, Gutierrez-Sanchez R, et al. (2004) Biochemical characterization of new strains of Trypanosoma cruzi and T. rangeli isolates from Peru and Mexico. Parasitol Res 94: 294-300.

834. Roellig DM, Brown EL, Barnabe C, Tibayrenc M, Steurer FJ, et al. (2008) Molecular typing of Trypanosoma cruzi isolates, United States. Emerg Infect Dis 14: 1123-1125.

835. Roellig DM, Gomez-Puerta LA, Mead DG, Pinto J, Ancca-Juarez J, et al. Hemi-nested PCR and RFLP methodologies for identifying blood meals of the Chagas disease vector, Triatoma infestans. PLoS One 2013 Sep 11;8(9):e74713 doi: 101371/journalpone0074713.

836. Roellig DM, McMillan K, Ellis AE, Vandeberg JL, Champagne DE, et al. Experimental infection of two South American reservoirs with four distinct strains of Trypanosoma cruzi. Parasitology 137: 959-966.

837. Roffe E, Rothfuchs AG, Santiago HC, Marino AP, Ribeiro-Gomes FL, et al. IL-10 limits parasite burden and protects against fatal myocarditis in a mouse model of Trypanosoma cruzi infection. J Immunol 2012 Jan 15;188(2):649-60 doi: 104049/jimmunol1003845 Epub 2011 Dec 12.

838. Rogers DJ, Randolph SE (2000) The global spread of malaria in a future, warmer world. Science 289: 1763-1766.

839. Rossi L (1995) Structural and non-structural disease underlying high-risk cardiac arrhythmias relevant to sports medicine. J Sports Med Phys Fitness 35: 79-86.

840. Rosypal AC, Cortes-Vecino JA, Gennari SM, Dubey JP, Tidwell RR, et al. (2007) Serological survey of Leishmania infantum and Trypanosoma cruzi in dogs from urban areas of Brazil and Colombia. Vet Parasitol 149: 172-177.

841. Rosypal AC, Hill R, Lewis S, Braxton K, Zajac AM, et al. Toxoplasma gondii and Trypanosoma cruzi antibodies in dogs from Virginia. Zoonoses Public Health 57: e76-80.

842. Rosypal AC, Tidwell RR, Lindsay DS (2007) Prevalence of antibodies to Leishmania infantum and Trypanosoma cruzi in wild canids from South Carolina. J Parasitol 93: 955-957.

843. Rosypal AC, Tripp S, Lewis S, Francis J, Stoskopf MK, et al. Survey of antibodies to Trypanosoma cruzi and Leishmania spp. in gray and red fox populations from North Carolina and Virginia. J Parasitol 96: 1230-1231.

844. Rowland ME, Maloney J, Cohen S, Yabsley MJ, Huang J, et al. Factors associated with Trypanosoma cruzi exposure among domestic canines in Tennessee. J Parasitol 96: 547-551.

845. Rowlands GJ, Mulatu W, Leak SG, Nagda SM, d'Ieteren GD (1999) Estimating the effects of tsetse control on livestock productivity--a case study in southwest Ethiopia. Trop Anim Health Prod 31: 279-294.

846. Ruan JP, Arhin GK, Ullu E, Tschudi C (2004) Functional characterization of a Trypanosoma brucei TATA-binding protein-related factor points to a universal regulator of transcription in trypanosomes. Mol Cell Biol 24: 9610-9618.

847. Ruan JP, Shen S, Ullu E, Tschudi C (2007) Evidence for a capping enzyme with specificity for the trypanosome spliced leader RNA. Mol Biochem Parasitol 156: 246-254.

848. Ruan JP, Ullu E, Tschudi C (2007) Characterization of the Trypanosoma brucei cap hypermethylase Tgs1. Mol Biochem Parasitol 155: 66-69.

849. Rudenko G (1999) Genes involved in phenotypic and antigenic variation in African trypanosomes and malaria. Curr Opin Microbiol 2: 651-656.

850. Rudenko G (1999) Mechanisms mediating antigenic variation in Trypanosoma brucei. Mem Inst Oswaldo Cruz 94: 235-237.

851. Rudenko G (2000) The polymorphic telomeres of the African Trypanosome trypanosoma brucei. Biochem Soc Trans 28: 536-540.

852. Ruiz Postigo JA, Franco JR, Simarro PP, Bassets G, Nangouma A (2001) [Cost of a national program to control human African trypanosomiasis in the high Mbomou region, Central African Republic]. Med Trop (Mars) 61: 422-424.

853. Ruiz-Narvaez EA What is a functional locus? Understanding the genetic basis of complex phenotypic traits. Med Hypotheses 76: 638-642.

854. Runckel C, Flenniken ML, Engel JC, Ruby JG, Ganem D, et al. Temporal analysis of the honey bee microbiome reveals four novel viruses and seasonal prevalence of known viruses, Nosema, and Crithidia. PLoS One 6: e20656.

855. Ruszczyk A, Joerink M, Guldenaar C, Hermsen T, Savelkoul HF, et al. (2008) cDNA expression library screening and identification of two novel antigens: ubiquitin and receptor for activated C kinase (RACK) homologue, of the fish parasite Trypanosoma carassii. Fish Shellfish Immunol 25: 84-90.

856. Ruvalcaba-Trejo LI, Sturm NR The Trypanosoma cruzi Sylvio X10 strain maxicircle sequence: the third musketeer. BMC Genomics 12: 58.

857. Rzad I, Pilecka-Rapacz M, Sobecka E (2007) Characteristics of the peripheral blood of the Anguillicola crassus (Kuwahara, Niimi et Tagaki, 1974) and Trypanosoma granulosum Laveran et Mesnil, 1902 infested eel (Anguilla anguilla L.) ascending the River Rega (north-western Poland). Wiad Parazytol 53: 319-324.

858. Sabino EC, Lee TH, Montalvo L, Nguyen ML, Leiby DA, et al. Antibody levels correlate with detection of Trypanosoma cruzi DNA by sensitive polymerase chain reaction assays in seropositive blood donors and possible resolution of infection over time. Transfusion 2013 Jun;53(6):1257-65 doi: 101111/j1537-2995201203902x Epub 2012 Sep 25.

859. Sabino EC, Ribeiro AL, Salemi VM, Di Lorenzo Oliveira C, Antunes AP, et al. Ten-year incidence of Chagas cardiomyopathy among asymptomatic Trypanosoma cruzi-seropositive former blood donors. Circulation 2013 Mar 12;127(10):1105-15 doi: 101161/CIRCULATIONAHA112123612 Epub 2013 Feb 7.

860. Sadd BM, Barribeau SM Heterogeneity in infection outcome: lessons from a bumblebee-trypanosome system. Parasite Immunol 2013 Jun 12 doi: 101111/pim12043.

861. Said G (2007) Infectious neuropathies. Neurol Clin 25: 115-137.

862. Salathe R, Tognazzo M, Schmid-Hempel R, Schmid-Hempel P Probing mixed-genotype infections I: extraction and cloning of infections from hosts of the trypanosomatid Crithidia bombi. PLoS One 2012;7(11):e49046 doi: 101371/journalpone0049046 Epub 2012 Nov 14.

863. Salathe RM, Schmid-Hempel P The genotypic structure of a multi-host bumblebee parasite suggests a role for ecological niche overlap. PLoS One 6: e22054.

864. Salomon CJ First century of Chagas' disease: an overview on novel approaches to nifurtimox and benzonidazole delivery systems. J Pharm Sci 2012 Mar;101(3):888-94 doi: 101002/jps23010 Epub 2011 Dec 12.

865. Sanchez LV, Ramirez JD Congenital and oral transmission of American trypanosomiasis: an overview of physiopathogenic aspects. Parasitology 2013 Feb;140(2):147-59 doi: 101017/S0031182012001394 Epub 2012 Sep 25.

866. Sanchez-Guillen MC, Barnabe C, Guegan JF, Tibayrenc M, Velasquez-Rojas M, et al. (2002) High prevalence anti-Trypanosoma cruzi antibodies, among blood donors in the State of Puebla, a non-endemic area of Mexico. Mem Inst Oswaldo Cruz 97: 947-952.

867. Sanchez-Martin MJ, Feliciangeli MD, Campbell-Lendrum D, Davies CR (2006) Could the Chagas disease elimination programme in Venezuela be compromised by reinvasion of houses by sylvatic Rhodnius prolixus bug populations? Trop Med Int Health 11: 1585-1593.

868. Sanchez-Sancho F, Campillo NE, Paez JA Chagas disease: progress and new perspectives. Curr Med Chem 17: 423-452.

869. Sandor G, Mattern P (1969) [Study of the serum of Africans presenting various forms of reactional macroglobulinemia]. Bull Soc Pathol Exot Filiales 62: 780-789.

870. Santiago HC, Gonzalez Lombana CZ, Macedo JP, Utsch L, Tafuri WL, et al. NADPH phagocyte oxidase knockout mice control Trypanosoma cruzi proliferation, but develop circulatory collapse and succumb to infection. PLoS Negl Trop Dis 2012;6(2):e1492 doi: 101371/journalpntd0001492 Epub 2012 Feb 14.

871. Santos Lima EC, Minoprio P (1996) Chagas' disease is attenuated in mice lacking gamma delta T cells. Infect Immun 64: 215-221.

872. Santos-Buch CA, Acosta AM, Zweerink HJ, Sadigursky M, Andersen OF, et al. (1985) Primary muscle disease: definition of a 25-kDa polypeptide myopathic specific chagas antigen. Clin Immunol Immunopathol 37: 334-350.

873. Savage AF, Cerqueira GC, Regmi S, Wu Y, El Sayed NM, et al. Transcript expression analysis of putative Trypanosoma brucei GPI-anchored surface proteins during development in the tsetse and mammalian hosts. PLoS Negl Trop Dis 2012;6(6):e1708 doi: 101371/journalpntd0001708 Epub 2012 Jun 19.

874. Schares G, Maksimov A, Basso W, More G, Dubey JP, et al. Quantitative real time polymerase chain reaction assays for the sensitive detection of Besnoitia besnoiti infection in cattle. Vet Parasitol 178: 208-216.

875. Schares G, Mehlitz D (1996) Sleeping sickness in Zaire: a nested polymerase chain reaction improves the identification of Trypanosoma (Trypanozoon) brucei gambiense by specific kinetoplast DNA probes. Trop Med Int Health 1: 59-70.

876. Schaub GA, Jensen C (1990) Developmental time and mortality of the reduviid bug Triatoma infestans with differential exposure to coprophagic infections with Blastocrithidia triatomae (Trypanosomatidae). J Invertebr Pathol 55: 17-27.

877. Schauer R, Kamerling JP The chemistry and biology of trypanosomal trans-sialidases: virulence factors in Chagas disease and sleeping sickness. Chembiochem 12: 2246-2264.

878. Schenone H, Rojas A (1989) [Pragmatic data and observations related to the epidemiology of Chagas disease]. Bol Chil Parasitol 44: 66-86.

879. Scheuerlein A, Ricklefs RE (2004) Prevalence of blood parasites in European passeriform birds. Proc Biol Sci 271: 1363-1370.

880. Schiffler RJ, Mansur GP, Navin TR, Limpakarnjanarat K (1984) Indigenous Chagas' disease (American trypanosomiasis) in California. JAMA 251: 2983-2984.

881. Schlafer DH (1979) Trypanosoma theileri: a literature review and report of incidence in New York cattle. Cornell Vet 69: 411-425.

882. Schmid C, Kuemmerle A, Blum J, Ghabri S, Kande V, et al. In-hospital safety in field conditions of nifurtimox eflornithine combination therapy (NECT) for T. b. gambiense sleeping sickness. PLoS Negl Trop Dis 2012;6(11):e1920 doi: 101371/journalpntd0001920 Epub 2012 Nov 29.

883. Schmid C, Tjan TD, Etz C, Schmidt C, Wenzelburger F, et al. (2005) First clinical experience with the Incor left ventricular assist device. J Heart Lung Transplant 24: 1188-1194.

884. Schneider J (1960) [The diagnosis of African trypanosomiasis (sleeping sickness) in France]. Presse Med 68: 529-530.

885. Schneider J (1964) [DIAGNOSTIC AND THERAPEUTIC PROBLEMS OF TROPICAL MEDICINE IN CURRENT MEDICAL PRACTICE IN FRANCE]. Bull Soc Pathol Exot Filiales 57: 669-715.

886. Schulz MG (1971) [Parasitic diseases along the Mexican-United States border]. Salud Publica Mex 13: 377-380.

887. Sciarretta A, Girma M, Tikubet G, Belayehun L, Ballo S, et al. (2005) Development of an adaptive tsetse population management scheme for the Luke community, Ethiopia. J Med Entomol 42: 1006-1019.

888. Seah SK, Flegel KM (1972) African trypanosomiasis in Canada. Can Med Assoc J 106: 902-903.

889. Seden K, Khoo S, Back D, Prevatt N, Lamorde M, et al. Drug-drug interactions between antiretrovirals and drugs used in the management of neglected tropical diseases: important considerations in the WHO 2020 Roadmap and London Declaration on Neglected Tropical Diseases: AIDS. 2013 Mar 13;27(5):675-86. doi: 10.1097/QAD.0b013e32835ca9b4.

890. Sehgal RN, Jones HI, Smith TB (2001) Host specificity and incidence of Trypanosoma in some African rainforest birds: a molecular approach. Mol Ecol 10: 2319-2327.

891. Sehgal RN, Jones HI, Smith TB (2005) Blood parasites of some West African rainforest birds. J Vet Med Sci 67: 295-301.

892. Sehgal RN, Valkiunas G, Iezhova TA, Smith TB (2006) Blood parasites of chickens in Uganda and Cameroon with molecular descriptions of Leucocytozoon schoutedeni and Trypanosoma gallinarum. J Parasitol 92: 1336-1343.

893. Shadomy SV, Waring SC, Martins-Filho OA, Oliveira RC, Chappell CL (2004) Combined use of enzyme-linked immunosorbent assay and flow cytometry to detect antibodies to Trypanosoma cruzi in domestic canines in Texas. Clin Diagn Lab Immunol 11: 313-319.

894. Shah JJ, Maloney SA, Liu Y, Flagg EW, Johnston SP, et al. (2008) Evaluation of the impact of overseas pre-departure treatment for infection with intestinal parasites among Montagnard refugees migrating from Cambodia to North Carolina. Am J Trop Med Hyg 78: 754-759.

895. Shapiro DS (2002) Infectious disease surveillance update. Lancet Infect Dis 2: 7.

896. Shi M, Wei G, Pan W, Tabel H (2006) Experimental African trypanosomiasis: a subset of pathogenic, IFN-gamma-producing, MHC class II-restricted CD4+ T cells mediates early mortality in highly susceptible mice. J Immunol 176: 1724-1732.

897. Shi MQ, Wang CR, Wei GJ, Pan WL, Appleyard G, et al. (2006) Experimental African trypanosomiasis: lack of effective CD1d-restricted antigen presentation. Parasite Immunol 28: 643-647.

898. Shi MQ, Wei GJ, Tabel H (2007) Trypanosoma congolense infections: MHC class II-restricted immune responses mediate either protection or disease, depending on IL-10 function. Parasite Immunol 29: 107-111.

899. Shulman IA (1991) Parasitic infections, an uncommon risk of blood transfusion in the United States. Transfusion 31: 479-480.

900. Shulman IA (1994) Parasitic infections and their impact on blood donor selection and testing. Arch Pathol Lab Med 118: 366-370.

901. Shulman IA (1999) Intervention strategies to reduce the risk of transfusion-transmitted Trypanosoma cruzi infection in the United States. Transfus Med Rev 13: 227-234.

902. Shulman IA, Appleman MD (1991) Transmission of parasitic and bacterial infections through blood transfusion within the U.S. Crit Rev Clin Lab Sci 28: 447-459.

903. Sicuri E, Munoz J, Pinazo MJ, Posada E, Sanchez J, et al. Economic evaluation of Chagas disease screening of pregnant Latin American women and of their infants in a non endemic area. Acta Trop 118: 110-117.

904. Silva R, Moura-Neto RS (2004) Genetic diversity and admixture data on 11 STRs (F13B, TPOX, CSF1PO, F13A01, D7S820, LPL, TH01, vWA, D13S317, FESFPS, and D16S539) in a sample of Rio de Janeiro European-descendants population, Brazil. Forensic Sci Int 142: 51-53.

905. Silveira FT, Blackwell JM, Ishikawa EA, Braga R, Shaw JJ, et al. (1998) T cell responses to crude and defined leishmanial antigens in patients from the lower Amazon region of Brazil infected with different species of Leishmania of the subgenera Leishmania and Viannia. Parasite Immunol 20: 19-26.

906. Silveira FT, Carneiro LA, Ramos PK, Chagas EJ, Lima LV, et al. A cross-sectional study on canine Leishmania (L.) infantum chagasi infection in Amazonian Brazil ratifies a higher prevalence of specific IgG-antibody response than delayed-type hypersensitivity in symptomatic and asymptomatic dogs. Parasitol Res 2012 Oct;111(4):1513-22 Epub 2012 Jun 16.

907. Silveira FT, Lainson R, Crescente JA, de Souza AA, Campos MB, et al. A prospective study on the dynamics of the clinical and immunological evolution of human Leishmania (L.) infantum chagasi infection in the Brazilian Amazon region. Trans R Soc Trop Med Hyg 104: 529-535.

908. Simarro PP, Cecchi G, Franco JR, Paone M, Diarra A, et al. Estimating and mapping the population at risk of sleeping sickness. PLoS Negl Trop Dis 2012;6(10):e1859 doi: 101371/journalpntd0001859 Epub 2012 Oct 25.

909. Simarro PP, Cecchi G, Franco JR, Paone M, Fevre EM, et al. Risk for human African trypanosomiasis, Central Africa, 2000-2009. Emerg Infect Dis 2011 Dec;17(12):2322-4 doi: 103201/eid1712110921.

910. Simarro PP, Cecchi G, Paone M, Franco JR, Diarra A, et al. The Atlas of human African trypanosomiasis: a contribution to global mapping of neglected tropical diseases. Int J Health Geogr 9: 57.

911. Simarro PP, Diarra A, Ruiz Postigo JA, Franco JR, Jannin JG The human African trypanosomiasis control and surveillance programme of the World Health Organization 2000-2009: the way forward. PLoS Negl Trop Dis 5: e1007.

912. Simarro PP, Franco JR, Cecchi G, Paone M, Diarra A, et al. Human African trypanosomiasis in non-endemic countries (2000-2010). J Travel Med 2012 Jan-Feb;19(1):44-53 doi: 101111/j1708-8305201100576x Epub 2011 Dec 8.

913. Simarro PP, Jannin J, Cattand P (2008) Eliminating human African trypanosomiasis: where do we stand and what comes next? PLoS Med 5: e55.

914. Simukoko H, Marcotty T, Phiri I, Geysen D, Vercruysse J, et al. (2007) The comparative role of cattle, goats and pigs in the epidemiology of livestock trypanosomiasis on the plateau of eastern Zambia. Vet Parasitol 147: 231-238.

915. Simukoko H, Marcotty T, Phiri I, Vercruysse J, Van den Bossche P (2007) Heterogeneity in the trypanosomosis incidence in Zebu cattle of different ages and sex on the plateau of eastern Zambia. Acta Trop 103: 98-101.

916. Simukoko H, Marcotty T, Vercruysse J, Van den Bossche P Bovine trypanosomiasis risk in an endemic area on the eastern plateau of Zambia. Res Vet Sci 90: 51-54.

917. Singh B, Kalra IS, Gupta MP, Nauriyal DC (1993) Trypanosoma evansi infection in dogs: seasonal prevalence and chemotherapy. Vet Parasitol 50: 137-141.

918. Smith A, Telfer S, Burthe S, Bennett M, Begon M (2006) A role for vector-independent transmission in rodent trypanosome infection? Int J Parasitol 36: 1359-1366.

919. Smith BA, Conlan C, Hwang WS, Weirauch C Polymerase chain reaction detection of Trypanosoma cruzi in suboptimally preserved vectors and comparative infection rates 2007-2010 in Escondido, Southern California. Vector Borne Zoonotic Dis 11: 1603-1604.

920. Solano P, Kone A, Garcia A, Sane B, Michel V, et al. (2003) [Role of patient travel in transmission of human African trypanosomiasis in a highly endemic area of the Ivory Coast]. Med Trop (Mars) 63: 577-582.

921. Solano P, Michel JF, Lefrancois T, de La Rocque S, Sidibe I, et al. (1999) Polymerase chain reaction as a diagnosis tool for detecting trypanosomes in naturally infected cattle in Burkina Faso. Vet Parasitol 86: 95-103.

922. Sommese L, Fiorito C, Napoli C The relationship between Chagas disease and immunosuppressive therapy. Blood Transfus 2013 May 28:1-2 doi: 102450/20130079-13.

923. Sosa-Estani S, Gamboa-Leon MR, Del Cid-Lemus J, Althabe F, Alger J, et al. (2008) Use of a rapid test on umbilical cord blood to screen for Trypanosoma cruzi infection in pregnant women in Argentina, Bolivia, Honduras, and Mexico. Am J Trop Med Hyg 79: 755-759.

924. Sousa JX, Jr., Diaz LA, Eaton DP, Hans-Filho G, Freitas EL, et al. Profile of Trypanosoma cruzi reactivity in a population at high risk for endemic pemphigus foliaceus (Fogo selvagem). Am J Trop Med Hyg 2012 Oct;87(4):675-80 doi: 104269/ajtmh201212-0206 Epub 2012 Jul 23.

925. Spencer HC, Jr., Gibson JJ, Jr., Brodsky RE, Schultz MG (1975) Imported African trypanosomiasis in the United States. Ann Intern Med 82: 633-638.

926. Spurling G, Lucas R, Doust J (2005) Identifying health centers in honduras infested with Rhodnius prolixus using the seroprevalence of Chagas disease in children younger than 13 years. Am J Trop Med Hyg 73: 307-308.

927. Srivastava P, Prajapati VK, Vanaerschot M, Van der Auwera G, Dujardin JC, et al. Detection of Leptomonas sp. parasites in clinical isolates of Kala-azar patients from India. Infect Genet Evol 10: 1145-1150.

928. Stabler RM, Holt PA, Kitzmiller NJ (1966) Trypanosoma avium in the blood and bone marrow from 677 Colorado birds. J Parasitol 52: 1141-1144.

929. Stabler RM, Kitzmiller NJ (1970) Hematozoa from Colorado birds. 3. Passeriformes. J Parasitol 56: 12-16.

930. Stalos S, Land K (2007) Chagas disease. Tex Med 103: 45-46.

931. Stein J, Ayalew W, Rege E, Mulatu W, Lemecha H, et al. Trypanosomosis and phenotypic features of four indigenous cattle breeds in an Ethiopian field study. Vet Parasitol 178: 40-47.

932. Stein J, Ayalew W, Rege JE, Mulatu W, Malmfors B, et al. (2009) Livestock keeper perceptions of four indigenous cattle breeds in tsetse infested areas of Ethiopia. Trop Anim Health Prod 41: 1335-1346.

933. Stenberg PL, Bowerman WJ (2008) Hemoparasites in Oregon spotted frogs (Rana pretiosa) from central Oregon, USA. J Wildl Dis 44: 464-468.

934. Stephenson J (2002) Researchers wrestle with spread and control of emerging infections. JAMA 287: 2061-2063.

935. Sternberg JM, Maclean L A spectrum of disease in human African trypanosomiasis: the host and parasite genetics of virulence. Parasitology 137: 2007-2015.

936. Stevens JR, Welburn SC (1993) Genetic processes within an epidemic of sleeping sickness in Uganda. Parasitol Res 79: 421-427.

937. Stevens L, Rizzo DM, Lucero DE, Pizarro JC Household model of Chagas disease vectors (Hemiptera: Reduviidae) considering domestic, peridomestic, and sylvatic vector populations. J Med Entomol 2013 Jul;50(4):907-15.

938. Stevenson P, Okech G, Mwendia C, Sones KR (2000) Comparison of the isometamidium-based trypanocidal drugs Samorin and Veridium in cattle under field conditions at Nguruman, Kenya. Acta Trop 77: 195-201.

939. Stich A, Abel PM, Krishna S (2002) Human African trypanosomiasis. BMJ 325: 203-206.

940. Stich A, Barrett MP, Krishna S (2003) Waking up to sleeping sickness. Trends Parasitol 19: 195-197.

941. Strasen J, Williams T, Ertl G, Zoller T, Stich A, et al. Epidemiology of Chagas disease in Europe: many calculations, little knowledge. Clin Res Cardiol 2013 Aug 29.

942. Stuht JN (1975) Morphology of typanosomes from white-tailed deer and wapiti in Michigan. J Wildl Dis 11: 256-262.

943. Sunnemark D, Andersson R, Harris RA, Jeddi-Tehrani M, Orn A (1998) Enhanced prevalence of T cells expressing TCRBV8S2 and TCRBV8S3 in hearts of chronically Trypanosoma cruzi-infected mice. Immunol Lett 60: 171-177.

944. Sures B, Knopf K, Wurtz J, Hirt J (1999) Richness and diversity of parasite communities in European eels Anguilla anguilla of the River Rhine, Germany, with special reference to helminth parasites. Parasitology 119 ( Pt 3): 323-330.

945. Swezey RL (1963) "KISSING BUG" BITE IN LOS ANGELES. Arch Intern Med 112: 977-980.

946. Sztajzel J, Cox J, Pache JC, Badaoui E, Lerch R, et al. (1996) Chagas' disease may also be encountered in Europe. Eur Heart J 17: 1289.

947. Tait A, Morrison LJ, Duffy CW, Cooper A, Turner CM, et al. Trypanosome genetics: populations, phenotypes and diversity. Vet Parasitol 181: 61-68.

948. Tamarit A, Gutierrez C, Arroyo R, Jimenez V, Zagala G, et al. Trypanosoma evansi infection in mainland Spain. Vet Parasitol 167: 74-76.

949. Tamarit A, Tejedor-Junco MT, Gonzalez M, Alberola J, Gutierrez C Morphological and biometrical features of Trypanosoma evansi isolates from an outbreak in mainland Spain. Vet Parasitol 177: 152-156.

950. Tan JS (1978) Common and uncommon parasitic infections in the United States. Med Clin North Am 62: 1059-1081.

951. Tanowitz HB, Huang H, Jelicks LA, Chandra M, Loredo ML, et al. (2005) Role of endothelin 1 in the pathogenesis of chronic chagasic heart disease. Infect Immun 73: 2496-2503.

952. Tanowitz HB, Kirchhoff LV, Simon D, Morris SA, Weiss LM, et al. (1992) Chagas' disease. Clin Microbiol Rev 5: 400-419.

953. Tanowitz HB, Machado FS, Jelicks LA, Shirani J, de Carvalho AC, et al. (2009) Perspectives on Trypanosoma cruzi-induced heart disease (Chagas disease). Prog Cardiovasc Dis 51: 524-539.

954. Tarleton RL, Curran JW Is Chagas disease really the "new HIV/AIDS of the Americas"? PLoS Negl Trop Dis 2012;6(10):e1861 doi: 101371/journalpntd0001861 Epub 2012 Oct 25.

955. Tarleton RL, Grusby MJ, Postan M, Glimcher LH (1996) Trypanosoma cruzi infection in MHC-deficient mice: further evidence for the role of both class I- and class II-restricted T cells in immune resistance and disease. Int Immunol 8: 13-22.

956. Tarleton RL, Reithinger R, Urbina JA, Kitron U, Gurtler RE (2007) The challenges of Chagas Disease-- grim outlook or glimmer of hope. PLoS Med 4: e332.

957. Tatem AJ, Hay SI (2004) Measuring urbanization pattern and extent for malaria research: a review of remote sensing approaches. J Urban Health 81: 363-376.

958. Taverne J (1999) Unheard-of numbers and invitations on the Web. Parasitol Today 15: 313-314.

959. Telenti A (2009) Safety concerns about CCR5 as an antiviral target. Curr Opin HIV AIDS 4: 131-135.

960. Telford SR, Jr., Forrester DJ (1991) Hemoparasites of raccoons (Procyon lotor) in Florida. J Wildl Dis 27: 486-490.

961. Terblanche JS, Klok CJ, Krafsur ES, Chown SL (2006) Phenotypic plasticity and geographic variation in thermal tolerance and water loss of the tsetse Glossina pallidipes (Diptera: Glossinidae): implications for distribution modelling. Am J Trop Med Hyg 74: 786-794.

962. Theiler RN, Rasmussen SA, Treadwell TA, Jamieson DJ (2008) Emerging and zoonotic infections in women. Infect Dis Clin North Am 22: 755-772, vii-viii.

963. Theis JH, Tibayrenc M, Ault SK, Mason DT (1985) Agent of Chagas' disease from Honduran vector capable of developing in California insects: implications for cardiologists. Am Heart J 110: 605-608.

964. Thomas LF, de Glanville WA, Cook EA, Fevre EM The spatial ecology of free-ranging domestic pigs (Sus scrofa) in western Kenya. BMC Vet Res 2013 Mar 7;9:46 doi: 101186/1746-6148-9-46.

965. Thornton SJ, Wasan KM, Piecuch A, Lynd LL, Wasan EK Barriers to treatment for visceral leishmaniasis in hyperendemic areas: India, Bangladesh, Nepal, Brazil and Sudan. Drug Dev Ind Pharm 36: 1312-1319.

966. Tibayrenc M Modelling the transmission of Trypanosoma cruzi: the need for an integrated genetic epidemiological and population genomics approach. Adv Exp Med Biol 673: 200-211.

967. Tibayrenc M (1998) Integrated genetic epidemiology of infectious diseases: the Chagas model. Mem Inst Oswaldo Cruz 93: 577-580.

968. Tibayrenc M (2003) Genetic subdivisions within Trypanosoma cruzi (Discrete Typing Units) and their relevance for molecular epidemiology and experimental evolution. Kinetoplastid Biol Dis 2: 12.

969. Tibayrenc M, Ayala FJ How clonal are Trypanosoma and Leishmania? Trends Parasitol 2013 Jun;29(6):264-9 doi: 101016/jpt201303007 Epub 2013 Apr 17.

970. Tibayrenc M, Ayala FJ (1999) Evolutionary genetics of Trypanosoma and Leishmania. Microbes Infect 1: 465-472.

971. Tieszen KL, Molyneux DH (1989) Transmission and ecology of trypanosomatid flagellates of water striders (Hemiptera: Gerridae). J Protozool 36: 519-523.

972. Tilley H (2004) Ecologies of complexity: Tropical environments, African trypanosomiasis, and the science of disease control in British colonial Africa, 1900-1940. Osiris 19: 21-38.

973. Todd IP, Porter NH, Morson BC, Smith B, Friedmann CA, et al. (1969) Chagas disease of the colon and rectum. Gut 10: 1009-1014.

974. Tomazi L, Kawashita SY, Pereira PM, Zingales B, Briones MR (2009) Haplotype distribution of five nuclear genes based on network genealogies and Bayesian inference indicates that Trypanosoma cruzi hybrid strains are polyphyletic. Genet Mol Res 8: 458-476.

975. Tong J, Valverde O, Mahoudeau C, Yun O, Chappuis F Challenges of controlling sleeping sickness in areas of violent conflict: experience in the Democratic Republic of Congo. Confl Health 5: 7.

976. Torr SJ, Chamisa A, Mangwiro TN, Vale GA Where, when and why do tsetse contact humans? Answers from studies in a national park of Zimbabwe. PLoS Negl Trop Dis 2012;6(8):e1791 doi: 101371/journalpntd0001791 Epub 2012 Aug 28.

977. Torr SJ, Vale GA Is the even distribution of insecticide-treated cattle essential for tsetse control? Modelling the impact of baits in heterogeneous environments. PLoS Negl Trop Dis 5: e1360.

978. Touratier L (1993) First International Seminar on Non Tsetse-Transmitted Animal Trypanosomoses: conclusions and recommendations. Rev Sci Tech 12: 273-281.

979. Touratier L (1999) The Office International des epizooties ad hoc group on non tsetse animal trypanosomoses: its origin, scope and perspectives. Mem Inst Oswaldo Cruz 94: 191-194.

980. Traub-Cseko YM, Duboise M, Boukai LK, McMahon-Pratt D (1993) Identification of two distinct cysteine proteinase genes of Leishmania pifanoi axenic amastigotes using the polymerase chain reaction. Mol Biochem Parasitol 57: 101-115.

981. Trouiller P, Rey JL, Bouscharain P (2000) [Pharmaceutical development concerning diseases predominating in tropical regions: the concept of indigent drugs]. Ann Pharm Fr 58: 43-46.

982. Truc P, Grebaut P, Lando A, Makiadi Donzoau F, Penchenier L, et al. Epidemiological aspects of the transmission of the parasites causing human African trypanosomiasis in Angola. Ann Trop Med Parasitol 105: 261-265.

983. Truc P, Lando A, Penchenier L, Vatunga G, Josenando T Human African trypanosomiasis in Angola: clinical observations, treatment, and use of PCR for stage determination of early stage of the disease. Trans R Soc Trop Med Hyg 106: 10-14.

984. Truc P, Ravel S, Jamonneau V, N'Guessan P, Cuny G (2002) Genetic variability within Trypanosoma brucei gambiense: evidence for the circulation of different genotypes in human African trypanosomiasis patients in Cote d'Ivoire. Trans R Soc Trop Med Hyg 96: 52-55.

985. Truc P, Tibayrenc M (1993) Population genetics of Trypanosoma brucei in central Africa: taxonomic and epidemiological significance. Parasitology 106 ( Pt 2): 137-149.

986. Truc P, Tiouchichine ML, Cuny G, Vatunga G, Josenando T, et al. Multiple infections of Trypanosoma brucei gambiense in blood and cerebrospinal fluid of human African trypanosomosis patients from Angola: consequences on clinical course and treatment outcome. Infect Genet Evol 2012 Mar;12(2):399-402 doi: 101016/jmeegid201201010 Epub 2012 Jan 21.

987. Tustin AW, Small DS, Delgado S, Neyra RC, Verastegui MR, et al. Use of Individual-level Covariates to Improve Latent Class Analysis of Trypanosoma Cruzi Diagnostic Tests. Epidemiol Method 2012 Aug;1(1):33-54.

988. Ueno Y, Nakamura Y, Takahashi M, Inoue T, Endo S, et al. (1995) A highly suspected case of chronic Chagas' heart disease diagnosed in Japan. Jpn Circ J 59: 219-223.

989. Urbina JA, Docampo R (2003) Specific chemotherapy of Chagas disease: controversies and advances. Trends Parasitol 19: 495-501.

990. Urech K, Neumayr A, Blum J Sleeping sickness in travelers - do they really sleep? PLoS Negl Trop Dis 5: e1358.

991. Uzonna JE, Kaushik RS, Gordon JR, Tabel H (1998) Experimental murine Trypanosoma congolense infections. I. Administration of anti-IFN-gamma antibodies alters trypanosome-susceptible mice to a resistant-like phenotype. J Immunol 161: 5507-5515.

992. Vale GA, Chamisa A, Mangwiro C, Torr SJ A neglected aspect of the epidemiology of sleeping sickness: the propensity of the tsetse fly vector to enter houses. PLoS Negl Trop Dis 2013;7(2):e2086 doi: 101371/journalpntd0002086 Epub 2013 Feb 28.

993. Vale GA, Hall DR, Chamisa A, Torr SJ Towards an early warning system for Rhodesian sleeping sickness in savannah areas: man-like traps for tsetse flies. PLoS Negl Trop Dis 2012;6(12):e1978 doi: 101371/journalpntd0001978 Epub 2012 Dec 27.

994. Valkiunas G, Sehgal RN, Iezhova TA, Smith TB (2005) Further observations on the blood parasites of birds in Uganda. J Wildl Dis 41: 580-587.

995. van Dam JT, Schrama JW, van der Hel W, Verstegen MW, Zwart D (1996) Heat production, body temperature, and body posture in west African dwarf goats infected with Trypanosoma vivax. Vet Q 18: 55-59.

996. Van den Bossche P, de La Rocque S, Hendrickx G, Bouyer J A changing environment and the epidemiology of tsetse-transmitted livestock trypanosomiasis. Trends Parasitol 26: 236-243.

997. Van den Bossche P, Delespaux V Options for the control of tsetse-transmitted livestock trypanosomosis. An epidemiological perspective. Vet Parasitol 181: 37-42.

998. van den Burg PJ, Vrielink H, Reesink HW (1998) Donor selection: the exclusion of high risk donors? Vox Sang 74 Suppl 2: 499-502.

999. Van Der Kuip EJ (1969) Trypanosomiasis cruzi in Aruba and Curacao. Trop Geogr Med 21: 462-469.

1000. van der Sar A, Vinke B (1965) Investigation into the occurrence of Trypanosoma cruzi in Curacao. Trop Geogr Med 17: 225-228.

1001. Vanhecke C, Guevart E, Ezzedine K, Receveur MC, Jamonneau V, et al. [Human African trypanosomiasis in mangrove epidemiologic area. Presentation, diagnosis and treatment in Guinea, 2005-2007]. Pathol Biol (Paris) 58: 110-116.

1002. Vazquez-Prokopec GM, Cecere MC, Kitron U, Gurtler RE (2008) Environmental and demographic factors determining the spatial distribution of Triatoma guasayana in peridomestic and semi-sylvatic habitats of rural northwestern Argentina. Med Vet Entomol 22: 273-282.

1003. Vazquez-Prokopec GM, Spillmann C, Zaidenberg M, Gurtler RE, Kitron U Spatial heterogeneity and risk maps of community infestation by Triatoma infestans in rural northwestern Argentina. PLoS Negl Trop Dis 2012;6(8):e1788 doi: 101371/journalpntd0001788 Epub 2012 Aug 14.

1004. Vazquez-Prokopec GM, Spillmann C, Zaidenberg M, Kitron U, Gurtler RE (2009) Cost-effectiveness of chagas disease vector control strategies in Northwestern Argentina. PLoS Negl Trop Dis 3: e363.

1005. Veloso VG, Bastos FI, Portela MC, Grinsztejn B, Joao EC, et al. HIV rapid testing as a key strategy for prevention of mother-to-child transmission in Brazil. Rev Saude Publica 44: 803-811.

1006. Villa L, Morote S, Bernal O, Bulla D, Albajar-Vinas P (2007) Access to diagnosis and treatment of Chagas disease/infection in endemic and non-endemic countries in the XXI century. Mem Inst Oswaldo Cruz 102 Suppl 1: 87-94.

1007. Villalba R, Fornes G, Alvarez MA, Roman J, Rubio V, et al. (1992) Acute Chagas' disease in a recipient of a bone marrow transplant in Spain: case report. Clin Infect Dis 14: 594-595.

1008. Vokaty S, Desquesnes M, Applewhaite L, Favre J, Lieuw AJR, et al. (1996) TRYPNET. New hemoparasite information network. Ann N Y Acad Sci 791: 166-171.

1009. von Wissmann B, Machila N, Picozzi K, Fevre EM, de CBBM, et al. Factors associated with acquisition of human infective and animal infective trypanosome infections in domestic livestock in Western Kenya. PLoS Negl Trop Dis 5: e941.

1010. Wacher TJ, Milligan PJ, Rawlings P, Snow WF (1994) Tsetse-trypanosomiasis challenge to village N'Dama cattle in The Gambia: field assessments of spatial and temporal patterns of tsetse-cattle contact and the risk of trypanosomiasis infection. Parasitology 109 ( Pt 2): 149-162.

1011. Wagner SJ, Skripchenko A, Salata J, Cardo LJ (2007) Photoinactivation of Trypanosoma cruzi in red cell suspensions with thiopyrylium. Transfus Apher Sci 37: 23-25.

1012. Wainwright M, Baptista MS The application of photosensitisers to tropical pathogens in the blood supply. Photodiagnosis Photodyn Ther 8: 240-248.

1013. Waleckx E, Salas R, Huaman N, Buitrago R, Bosseno MF, et al. New insights on the Chagas disease main vector Triatoma infestans (Reduviidae, Triatominae) brought by the genetic analysis of Bolivian sylvatic populations. Infect Genet Evol 11: 1045-1057.

1014. Walker MD, Zunt JR (2005) Neuroparasitic infections: cestodes, trematodes, and protozoans. Semin Neurol 25: 262-277.

1015. Walsh C, Bradley M, Nadeau K (1992) Molecular studies on trypanothione reductase: an antiparasitic target enzyme. Curr Top Cell Regul 33: 409-417.

1016. Walshe DP, Lehane MJ, Haines LR Post eclosion age predicts the prevalence of midgut trypanosome infections in Glossina. PLoS One 6: e26984.

1017. Walter A (2003) [Human activities and American trypanosomiasis. Review of the literature]. Parasite 10: 191-204.

1018. Walter G, Liebisch A (1980) [Studies of the ecology of some blood protozoa of wild small mammals in North Germany (author's transl)]. Acta Trop 37: 31-40.

1019. Wamwiri FN, Alam U, Thande PC, Aksoy E, Ngure RM, et al. Wolbachia, Sodalis and trypanosome co-infections in natural populations of Glossina austeni and Glossina pallidipes. Parasit Vectors 2013 Aug 8;6(1):232 doi: 101186/1756-3305-6-232.

1020. Wang J, Aksoy S PGRP-LB is a maternally transmitted immune milk protein that influences symbiosis and parasitism in tsetse's offspring. Proc Natl Acad Sci U S A 2012 Jun 26;109(26):10552-7 doi: 101073/pnas1116431109 Epub 2012 Jun 11.

1021. Wang J, Hu C, Wu Y, Stuart A, Amemiya C, et al. (2008) Characterization of the antimicrobial peptide attacin loci from Glossina morsitans. Insect Mol Biol 17: 293-302.

1022. Wang J, Weiss BL, Aksoy S Tsetse fly microbiota: form and function. Front Cell Infect Microbiol 2013 Oct 29;3:69 doi: 103389/fcimb201300069.

1023. Wang J, Wu Y, Yang G, Aksoy S (2009) Interactions between mutualist Wigglesworthia and tsetse peptidoglycan recognition protein (PGRP-LB) influence trypanosome transmission. Proc Natl Acad Sci U S A 106: 12133-12138.

1024. Ward DE (1995) Risk assessment related to veterinary biologicals: specific issues in developing nations. Rev Sci Tech 14: 991-1007.

1025. Wardrop NA, Atkinson PM, Gething PW, Fevre EM, Picozzi K, et al. Bayesian geostatistical analysis and prediction of Rhodesian human African trypanosomiasis. PLoS Negl Trop Dis 4: e914.

1026. Wardrop NA, Fevre EM, Atkinson PM, Kakembo A, Welburn SC An exploratory GIS-based method to identify and characterise landscapes with an elevated epidemiological risk of Rhodesian human African trypanosomiasis. BMC Infect Dis 2012 Nov 21;12:316 doi: 101186/1471-2334-12-316.

1027. Wasser WG, Tzur S, Wolday D, Adu D, Baumstein D, et al. Population genetics of chronic kidney disease: the evolving story of APOL1. J Nephrol 2012 Sep-Oct;25(5):603-18 doi: 105301/jn5000179.

1028. Wastling SL, Welburn SC Diagnosis of human sleeping sickness: sense and sensitivity. Trends Parasitol 27: 394-402.

1029. Watier-Grillot S (2008) [Outbreak of animal trypanosomiasis (T. evansi) in the Aveyron department of France: risk for implantation of an animal disease with zoonotic potential]. Med Trop (Mars) 68: 468-470.

1030. Watier-Grillot S, Herder S, Marie JL, Cuny G, Davoust B Chemoprophylaxis and treatment of African canine trypanosomosis in French military working dogs: a retrospective study. Vet Parasitol 2013 May 1;194(1):1-8 doi: 101016/jvetpar201301025 Epub 2013 Feb 4.

1031. Webert KE, Cserti CM, Hannon J, Lin Y, Pavenski K, et al. (2008) Proceedings of a Consensus Conference: pathogen inactivation-making decisions about new technologies. Transfus Med Rev 22: 1-34.

1032. Wei G, Tabel H (2008) Regulatory T cells prevent control of experimental African trypanosomiasis. J Immunol 180: 2514-2521.

1033. Weiss B, Aksoy S Microbiome influences on insect host vector competence. Trends Parasitol 27: 514-522.

1034. Weiss BL, Mouchotte R, Rio RV, Wu YN, Wu Z, et al. (2006) Interspecific transfer of bacterial endosymbionts between tsetse fly species: infection establishment and effect on host fitness. Appl Environ Microbiol 72: 7013-7021.

1035. Weiss BL, Wang J, Maltz MA, Wu Y, Aksoy S Trypanosome infection establishment in the tsetse fly gut is influenced by microbiome-regulated host immune barriers. PLoS Pathog 2013;9(4):e1003318 doi: 101371/journalppat1003318 Epub 2013 Apr 18.

1036. Welburn S, Picozzi K, Coleman PG, Packer C (2008) Patterns in age-seroprevalence consistent with acquired immunity against Trypanosoma brucei in Serengeti lions. PLoS Negl Trop Dis 2: e347.

1037. Welburn SC, Maudlin I (1992) The nature of the teneral state in Glossina and its role in the acquisition of trypanosome infection in tsetse. Ann Trop Med Parasitol 86: 529-536.

1038. Wells EA, Lumsden WH, McNeillage GJ (1968) Isolation of trypanosomes of the section stercoraria from cattle in Nigeria and the United Kingdom. Br Vet J 124: 382-392.

1039. Wilfert L, Longdon B, Ferreira AG, Bayer F, Jiggins FM Trypanosomatids are common and diverse parasites of Drosophila. Parasitology: 1-8.

1040. Willett KC (1965) TRYPANOSOMIASIS IN BRITAIN. Br Med J 2: 167.

1041. Williams GD, Adams LG, Yaeger RG, McGrath RK, Read WK, et al. (1977) Naturally occurring trypanosomiasis (Chagas' disease) in dogs. J Am Vet Med Assoc 171: 171-177.

1042. Williams JT, Mubiru JN, Schlabritz-Loutsevitch NE, Rubicz RC, VandeBerg JL, et al. (2009) Polymerase chain reaction detection of Trypanosoma cruzi in Macaca fascicularis using archived tissues. Am J Trop Med Hyg 81: 228-234.

1043. Williams NA, Calverley BK, Mahrt JL (1977) Blood parasites of mallard and pintail ducks from central Alberta and the Mackenzie Delta, Northwest Territories. J Wildl Dis 13: 226-229.

1044. Williams-Blangero S, Criscione CD, VandeBerg JL, Correa-Oliveira R, Williams KD, et al. Host genetics and population structure effects on parasitic disease. Philos Trans R Soc Lond B Biol Sci 2012 Mar 19;367(1590):887-94 doi: 101098/rstb20110296.

1045. Williams-Blangero S, VandeBerg JL, Blangero J, Correa-Oliveira R Genetic epidemiology of Chagas disease. Adv Parasitol 75: 147-167.

1046. Williams-Blangero S, VandeBerg JL, Blangero J, Correa-Oliveira R (2003) Genetic epidemiology of Trypanosoma cruzi infection and Chagas' disease. Front Biosci 8: e337-345.

1047. Wita I, Karbowiak G, Jezewski W (2001) [The prevalence of trypanosomes in bream Abramis brama in Goslawskie and Goplo lakes]. Wiad Parazytol 47: 383-387.

1048. Wizel B, Garg N, Tarleton RL (1998) Vaccination with trypomastigote surface antigen 1-encoding plasmid DNA confers protection against lethal Trypanosoma cruzi infection. Infect Immun 66: 5073-5081.

1049. Wizel B, Nunes M, Tarleton RL (1997) Identification of Trypanosoma cruzi trans-sialidase family members as targets of protective CD8+ TC1 responses. J Immunol 159: 6120-6130.

1050. Woo P, Soltys MA, Gillick AC (1970) Trypanosomes in cattle in southern Ontario. Can J Comp Med 34: 142-147.

1051. Wood SF (1975) New localities for mammal blood parasites from southwestern United States. J Parasitol 61: 969-970.

1052. Wood SF (1975) Trypanosoma cruzi: new foci of enzootic Chagas' disease in California. Exp Parasitol 38: 153-160.

1053. Woodruff AW (1971) United States Naval Medical Research Units in Africa. Br Med J 1: 551-552.

1054. Woody NC, Hernandez A, Suchow B (1965) AMERICAN TRYPANOSOMIASIS. 3. THE INCIDENCE OF SEROLOGICALLY DIAGNOSED CHAGAS' DISEASE AMONG PERSONS BITTEN BY THE INSECT VECTOR. J Pediatr 66: 107-109.

1055. Woolhouse ME, Hargrove JW (1998) On the interpretation of age-prevalence curves for trypanosome infections of tsetse flies. Parasitology 116 ( Pt 2): 149-156.

1056. Yabsley MJ, Noblet GP (2002) Seroprevalence of Trypanosoma cruzi in raccoons from South Carolina and Georgia. J Wildl Dis 38: 75-83.

1057. Yabsley MJ, Noblet GP, Pung OJ (2001) Comparison of serological methods and blood culture for detection of Trypanosoma cruzi infection in raccoons (Procyon lotor). J Parasitol 87: 1155-1159.

1058. Yaeger RG (1988) The prevalence of Trypanosoma cruzi infection in armadillos collected at a site near New Orleans, Louisiana. Am J Trop Med Hyg 38: 323-326.

1059. Yan J, Cheng Q, Li CB, Aksoy S (2001) Molecular characterization of two serine proteases expressed in gut tissue of the African trypanosome vector, Glossina morsitans morsitans. Insect Mol Biol 10: 47-56.

1060. Yeo M, Mauricio IL, Messenger LA, Lewis MD, Llewellyn MS, et al. Multilocus sequence typing (MLST) for lineage assignment and high resolution diversity studies in Trypanosoma cruzi. PLoS Negl Trop Dis 5: e1049.

1061. Young C, Losikoff P, Chawla A, Glasser L, Forman E (2007) Transfusion-acquired Trypanosoma cruzi infection. Transfusion 47: 540-544.

1062. Yun O, Lima MA, Ellman T, Chambi W, Castillo S, et al. (2009) Feasibility, drug safety, and effectiveness of etiological treatment programs for Chagas disease in Honduras, Guatemala, and Bolivia: 10-year experience of Medecins Sans Frontieres. PLoS Negl Trop Dis 3: e488.

1063. Zalis MG, Pang L, Silveira MS, Milhous WK, Wirth DF (1998) Characterization of Plasmodium falciparum isolated from the Amazon region of Brazil: evidence for quinine resistance. Am J Trop Med Hyg 58: 630-637.

1064. Zavizion B, Pereira M, de Melo Jorge M, Serebryanik D, Mather TN, et al. (2004) Inactivation of protozoan parasites in red blood cells using INACTINE PEN110 chemistry. Transfusion 44: 731-738.

1065. Zimmerman RH, Galardo AK, Lounibos LP, Arruda M, Wirtz R (2006) Bloodmeal hosts of Anopheles species (Diptera: Culicidae) in a malaria-endemic area of the Brazilian Amazon. J Med Entomol 43: 947-956.

1066. Zingales B, Miles MA, Campbell DA, Tibayrenc M, Macedo AM, et al. The revised Trypanosoma cruzi subspecific nomenclature: rationale, epidemiological relevance and research applications. Infect Genet Evol 2012 Mar;12(2):240-53 doi: 101016/jmeegid201112009 Epub 2011 Dec 27.

1067. Zinsstag J, Schelling E (2003) Vector-borne diseases in humans and animals: activities of the Swiss Tropical Institute and risks for Switzerland. Schweiz Arch Tierheilkd 145: 559-566, 568-559.

1068. Zoller T, Fevre EM, Welburn SC, Odiit M, Coleman PG (2008) Analysis of risk factors for T. brucei rhodesiense sleeping sickness within villages in south-east Uganda. BMC Infect Dis 8: 88.

1069. Zu Dohna H, Cecere MC, Gurtler RE, Kitron U, Cohen JE (2009) Spatial re-establishment dynamics of local populations of vectors of Chagas disease. PLoS Negl Trop Dis 3: e490.

1070. Zucca M, Savoia D Current developments in the therapy of protozoan infections. Open Med Chem J 5: 4-10.

***ARTICLES EXCLUDED AFTER REVIEWING THE ABSTRACT***

**ARTICLES NOT RELATED TO PREVALENCE OF CHAGAS DISEASE**

1. (2002) Chagas disease after organ transplantation--United States, 2001. MMWR Morb Mortal Wkly Rep 51: 210-212.

2. (2006) Chagas disease after organ transplantation--Los Angeles, California, 2006. MMWR Morb Mortal Wkly Rep 55: 798-800.

3. Aznar C, Liegeard P, Mariette C, Lafon S, Levin MJ, et al. (1997) A simple Trypanosoma cruzi enzyme-linked immunoassay for control of human infection in nonendemic areas. FEMS Immunol Med Microbiol 18: 31-37.

4. Aznar C, Lopez-Bergami P, Brandariz S, Mariette C, Liegeard P, et al. (1995) Prevalence of anti-R-13 antibodies in human Trypanosoma cruzi infection. FEMS Immunol Med Microbiol 12: 231-238.

5. Barbabosa-Pliego A, Diaz-Albiter HM, Ochoa-Garcia L, Aparicio-Burgos E, Lopez-Heydeck SM, et al. (2009) Trypanosoma cruzi circulating in the southern region of the State of Mexico (Zumpahuacan) are pathogenic: a dog model. Am J Trop Med Hyg 81: 390-395.

6. Barbabosa-Pliego A, Gil PC, Hernandez DO, Aparicio-Burgos JE, de Oca-Jimenez RM, et al. Prevalence of Trypanosoma cruzi in dogs (Canis familiaris) and triatomines during 2008 in a sanitary region of the State of Mexico, Mexico. Vector Borne Zoonotic Dis 11: 151-156.

7. Bayer AM, Hunter GC, Gilman RH, Cornejo Del Carpio JG, Naquira C, et al. (2009) Chagas disease, migration and community settlement patterns in Arequipa, Peru. PLoS Negl Trop Dis 3: e567.

8. Bern C, Kjos S, Yabsley MJ, Montgomery SP Trypanosoma cruzi and Chagas' Disease in the United States. Clin Microbiol Rev 24: 655-681.

9. Bern C, Montgomery SP, Herwaldt BL, Rassi A, Jr., Marin-Neto JA, et al. (2007) Evaluation and treatment of chagas disease in the United States: a systematic review. JAMA 298: 2171-2181.

10. Bern C, Verastegui M, Gilman RH, Lafuente C, Galdos-Cardenas G, et al. (2009) Congenital Trypanosoma cruzi transmission in Santa Cruz, Bolivia. Clin Infect Dis 49: 1667-1674.

11. Bowman NM, Kawai V, Levy MZ, Cornejo del Carpio JG, Cabrera L, et al. (2008) Chagas disease transmission in periurban communities of Arequipa, Peru. Clin Infect Dis 46: 1822-1828.

12. Bradley KK (1997) American trypanosomiasis: Chagas disease an emerging zoonotic threat in Oklahoma? J Okla State Med Assoc 90: 253-255.

13. Bradley KK, Bergman DK, Woods JP, Crutcher JM, Kirchhoff LV (2000) Prevalence of American trypanosomiasis (Chagas disease) among dogs in Oklahoma. J Am Vet Med Assoc 217: 1853-1857.

14. Brutus L, Castillo H, Bernal C, Salas NA, Schneider D, et al. Detectable Trypanosoma cruzi parasitemia during pregnancy and delivery as a risk factor for congenital Chagas disease. Am J Trop Med Hyg 83: 1044-1047.

15. Buekens P, Cafferata ML, Alger J, Althabe F, Belizan JM, et al. Congenital transmission of Trypanosoma cruzi in Argentina, Honduras, and Mexico: study protocol. Reprod Health 2013 Oct 11;10(1):55 doi: 101186/1742-4755-10-55.

16. Carrilero B, Murcia L, Martinez-Lage L, Segovia M Side effects of benznidazole treatment in a cohort of patients with Chagas disease in non-endemic country. Rev Esp Quimioter 24: 123-126.

17. Carvalheiro Jda R (2006) [Triatominae biogeography and the European cherry trees]. Rev Saude Publica 40: 1001-1003.

18. Castanos-Velez E, Maerlan S, Osorio LM, Aberg F, Biberfeld P, et al. (1998) Trypanosoma cruzi infection in tumor necrosis factor receptor p55-deficient mice. Infect Immun 66: 2960-2968.

19. Castillo C, Ramirez G, Valck C, Aguilar L, Maldonado I, et al. The interaction of classical complement component C1 with parasite and host calreticulin mediates Trypanosoma cruzi infection of human placenta. PLoS Negl Trop Dis 2013 Aug 22;7(8):e2376 doi: 101371/journalpntd0002376.

20. Castillo-Riquelme M, Guhl F, Turriago B, Pinto N, Rosas F, et al. (2008) The costs of preventing and treating chagas disease in Colombia. PLoS Negl Trop Dis 2: e336.

21. Castro E (2009) Chagas' disease: lessons from routine donation testing. Transfus Med 19: 16-23.

22. Cattand P, de Raadt P (1991) Laboratory diagnosis of trypanosomiasis. Clin Lab Med 11: 899-908.

23. Cesa K, Caillouet KA, Dorn PL, Wesson DM High Trypanosoma cruzi (Kinetoplastida: Trypanosomatidae) prevalence in Triatoma sanguisuga (Hemiptera: Redviidae) in southeastern Louisiana. J Med Entomol 48: 1091-1094.

24. Chippaux JP, Salas-Clavijo AN, Postigo JR, Schneider D, Santalla JA, et al. Evaluation of compliance to congenital Chagas disease treatment: results of a randomised trial in Bolivia. Trans R Soc Trop Med Hyg 2013 Jan;107(1):1-7 doi: 101093/trstmh/trs004.

25. Coffield DJ, Jr., Spagnuolo AM, Shillor M, Mema E, Pell B, et al. A model for Chagas disease with oral and congenital transmission. PLoS One 2013 Jun 28;8(6):e67267 doi: 101371/journalpone0067267 Print 2013.

26. Contreras MC, Schenone H, Borgono JM, Salinas P, Sandoval L, et al. (1992) [Chagasic infection in blood donors from hospitals in endemic regions of Chile (1982-1987). Epidemiological impact of the problem]. Bol Chil Parasitol 47: 10-15.

27. Cortez J, Providencia R, Ramos E, Valente C, Seixas J, et al. Emerging and under-recognized Chagas cardiomyopathy in non-endemic countries. World J Cardiol 2012 Jul 26;4(7):234-9 doi: 104330/wjcv4i7234.

28. Cruz FS, Marr JJ, Berens RL (1980) Prevention of transfusion-induced Chagas' disease by amphotericin B. Am J Trop Med Hyg 29: 761-765.

29. de Thoisy B, Michel JC, Vogel I, Vie JC (2000) A survey of hemoparasite infections in free-ranging mammals and reptiles in French Guiana. J Parasitol 86: 1035-1040.

30. Dedet JP, Pratlong F (2000) Leishmania, Trypanosoma and monoxenous trypanosomatids as emerging opportunistic agents. J Eukaryot Microbiol 47: 37-39.

31. Develoux M, Lescure FX, Jaureguiberry S, Jeannel D, Elghouzzi MH, et al. [Emergence of Chagas' disease in Europe: description of the first cases observed in Latin American immigrants in mainland France]. Med Trop (Mars) 70: 38-42.

32. Develoux M, Lescure FX, Le Loup G, Pialoux G (2009) [Chagas disease]. Rev Med Interne 30: 686-695.

33. Dias E (1951) [Chagas disease in the Americas. I. The United States]. Rev Bras Malariol Doencas Trop 3: 448-472.

34. Diaz JH (2007) Chagas disease in the United States: a cause for concern in Louisiana? J La State Med Soc 159: 21-23, 25-29.

35. Diaz JH (2008) Recognizing and reducing the risks of Chagas disease (American trypanosomiasis) in travelers. J Travel Med 15: 184-195.

36. Diaz-Suarez O (2009) [Chagas disease: re-emergent or neglected]. Invest Clin 50: 415-418.

37. Dobarro D, Gomez-Rubin C, Sanchez-Recalde A, Olias F, Bret-Zurita M, et al. (2008) Chagas' heart disease in Europe: an emergent disease? J Cardiovasc Med (Hagerstown) 9: 1263-1267.

38. Eggen BM (1995) [Blood transmission and infections]. Tidsskr Nor Laegeforen 115: 3035-3038.

39. Fitzpatrick S, Watts PC, Feliciangeli MD, Miles MA, Kemp SJ (2009) A panel of ten microsatellite loci for the Chagas disease vector Rhodnius prolixus (Hemiptera: Reduviidae). Infect Genet Evol 9: 206-209.

40. Flores-Chavez M, Faez Y, Olalla JM, Cruz I, Garate T, et al. (2008) Fatal congenital Chagas' disease in a non-endemic area: a case report. Cases J 1: 302.

41. Flores-Chavez M, Fernandez B, Puente S, Torres P, Rodriguez M, et al. (2008) Transfusional chagas disease: parasitological and serological monitoring of an infected recipient and blood donor. Clin Infect Dis 46: e44-47.

42. Gachelin G, Opinel A (2009) The reception by French physicians of Chagas' discovery of Trypanosoma cruzi and American trypanosomiasis (1909-1925). Hist Cienc Saude Manguinhos 16 Suppl 1: 35-56.

43. Garraud O, Pelletier B, Aznar C (2008) [Why defer blood donor candidates because of an exposure risk to Chagas disease?]. Transfus Clin Biol 15: 123-128.

44. Gascon J (2005) [Diagnosis and treatment of imported Chagas disease]. Med Clin (Barc) 125: 230-235.

45. Gascon J, Bern C, Pinazo MJ Chagas disease in Spain, the United States and other non-endemic countries. Acta Trop 115: 22-27.

46. Gascon J, Pinazo MJ (2008) [Controlling vertical transmission of Trypanosoma cruzi: the biggest challenge for imported disease in Spain]. Enferm Infecc Microbiol Clin 26: 607-608.

47. Gonzalez-Granado LI, Rojo-Conejo P, Ruiz-Contreras J, Gonzalez-Tome MI (2009) Chagas disease travels to Europe. Lancet 373: 2025.

48. Grant IH, Gold JW, Wittner M, Tanowitz HB, Nathan C, et al. (1989) Transfusion-associated acute Chagas disease acquired in the United States. Ann Intern Med 111: 849-851.

49. Guerri-Guttenberg RA, Ciannameo A, Di Girolamo C, Milei JJ (2009) [Chagas disease: an emerging public health problem in Italy?]. Infez Med 17: 5-13.

50. Guerri-Guttenberg RA, Di Girolamo C, Ciannameo A, Milei J (2009) [Migration flow and imported diseases: chronic chagasic cardiomyopathy]. G Ital Cardiol (Rome) 10: 234-240.

51. Guerri-Guttenberg RA, Grana DR, Ambrosio G, Milei J (2008) Chagas cardiomyopathy: Europe is not spared! Eur Heart J 29: 2587-2591.

52. Hagar JM, Rahimtoola SH (1991) Chagas' heart disease in the United States. N Engl J Med 325: 763-768.

53. Hanford EJ, Zhan FB, Lu Y, Giordano A (2007) Chagas disease in Texas: recognizing the significance and implications of evidence in the literature. Soc Sci Med 65: 60-79.

54. Hashimoto K, Yoshioka K Review: surveillance of Chagas disease. Adv Parasitol 2012;79:375-428 doi: 101016/B978-0-12-398457-900006-8.

55. Hemmige V, Tanowitz H, Sethi A Trypanosoma cruzi infection: a review with emphasis on cutaneous manifestations. Int J Dermatol 2012 May;51(5):501-8 doi: 101111/j1365-4632201105380x.

56. Holbert RD, Magiros E, Hirsch CP, Nunenmacher SJ (1995) Chagas' disease: a case in south Mississippi. J Miss State Med Assoc 36: 1-5.

57. Holguin A, Norman F, Martin L, Mateos ML, Chacon J, et al. Dried blood as an alternative to plasma or serum for Trypanosoma cruzi IgG detection in screening programs. Clin Vaccine Immunol 2013 Aug;20(8):1197-202 doi: 101128/CVI00221-13 Epub 2013 Jun 5.

58. Holmstad PR, Anwar A, Iezhova T, Skorping A (2003) Standard sampling techniques underestimate prevalence of avian hematozoa in willow ptarmigan (Lagopus lagopus). J Wildl Dis 39: 354-358.

59. Ikenga JO, Richerson JV (1984) Trypanosoma cruzi (Chagas) (protozoa: Kinetoplastida: Trypanosomatidae) in invertebrate and vertebrate hosts from Brewster County in Trans-Pecos Texas. J Econ Entomol 77: 126-129.

60. Irueta Isusi A, Jarque Moyano M, Redondo Ruiz P, Perez Martin V [Imported Chagas disease: alarm in Europe... as it should be]. Aten Primaria 44: 115-116.

61. Jackson Y (2009) [International migration: global issue, local impact: the example of two parasites]. Rev Med Suisse 5: 1022-1025.

62. Jackson Y, Alirol E, Getaz L, Wolff H, Combescure C, et al. Tolerance and safety of nifurtimox in patients with chronic chagas disease. Clin Infect Dis 51: e69-75.

63. Jackson Y, Angheben A, Carrilero Fernandez B, Jansa i Lopez del Vallado JM, Jannin JG, et al. (2009) [Management of Chagas disease in Europe. Experiences and challenges in Spain, Switzerland and Italy]. Bull Soc Pathol Exot 102: 326-329.

64. Jackson Y, Castillo S, Hammond P, Besson M, Brawand-Bron A, et al. Metabolic, mental health, behavioural and socioeconomic characteristics of migrants with Chagas disease in a non-endemic country. Trop Med Int Health 2012 May;17(5):595-603 doi: 101111/j1365-3156201202965x Epub 2012 Apr 5.

65. Jackson Y, Chappuis F, Loutan L (2008) [Chagas disease in Switzerland: managing an emerging infection and interrupting its transmission]. Rev Med Suisse 4: 1212-1214, 1216-1217.

66. Jimenez-Coello M, Acosta-Viana KY, Guzman-Marin E, Ortega-Pacheco A American trypanosomiasis infection in fattening pigs from the south-east of Mexico. Zoonoses Public Health 2012 Sep;59 Suppl 2:166-9 doi: 101111/zph12016.

67. Jimenez-Coello M, Poot-Cob M, Ortega-Pacheco A, Guzman-Marin E, Ramos-Ligonio A, et al. (2008) American trypanosomiasis in dogs from an urban and rural area of Yucatan, Mexico. Vector Borne Zoonotic Dis 8: 755-761.

68. Kapelusznik L, Varela D, Montgomery SP, Shah AN, Steurer FJ, et al. Chagas disease in Latin American immigrants with dilated cardiomyopathy in New York City. Clin Infect Dis 2013 Jul;57(1):e7 doi: 101093/cid/cit199 Epub 2013 Mar 28.

69. Kirchhoff LV Epidemiology of American trypanosomiasis (Chagas disease). Adv Parasitol 75: 1-18.

70. Kirchhoff LV, Neva FA (1985) Chagas' disease in Latin American immigrants. JAMA 254: 3058-3060.

71. Kirchhoff LV, Paredes P, Lomeli-Guerrero A, Paredes-Espinoza M, Ron-Guerrero CS, et al. (2006) Transfusion-associated Chagas disease (American trypanosomiasis) in Mexico: implications for transfusion medicine in the United States. Transfusion 46: 298-304.

72. Kun H, Moore A, Mascola L, Steurer F, Lawrence G, et al. (2009) Transmission of Trypanosoma cruzi by heart transplantation. Clin Infect Dis 48: 1534-1540.

73. Leiby DA, Herron RM, Jr., Garratty G, Herwaldt BL (2008) Trypanosoma cruzi parasitemia in US blood donors with serologic evidence of infection. J Infect Dis 198: 609-613.

74. Lescure FX, Canestri A, Melliez H, Jaureguiberry S, Develoux M, et al. (2008) Chagas disease, France. Emerg Infect Dis 14: 644-646.

75. Levy MZ, Kawai V, Bowman NM, Waller LA, Cabrera L, et al. (2007) Targeted screening strategies to detect Trypanosoma cruzi infection in children. PLoS Negl Trop Dis 1: e103.

76. Levy MZ, Small DS, Vilhena DA, Bowman NM, Kawai V, et al. Retracing micro-epidemics of Chagas disease using epicenter regression. PLoS Comput Biol 7: e1002146.

77. Lingam S, Marshall WC, Wilson J, Gould JM, Reinhardt MC, et al. (1985) Congenital trypanosomiasis in a child born in London. Dev Med Child Neurol 27: 670-674.

78. Machado FS, Jelicks LA, Kirchhoff LV, Shirani J, Nagajyothi F, et al. Chagas heart disease: report on recent developments. Cardiol Rev 2012 Mar-Apr;20(2):53-65 doi: 101097/CRD0b013e31823efde2.

79. Machado FS, Mukherjee S, Weiss LM, Tanowitz HB, Ashton AW Bioactive lipids in Trypanosoma cruzi infection. Adv Parasitol 76: 1-31.

80. Machado FS, Tanowitz HB, Teixeira MM New drugs for neglected infectious diseases: Chagas' disease. Br J Pharmacol 160: 258-259.

81. Marchiori PE, Alexandre PL, Britto N, Patzina RA, Fiorelli AA, et al. (2007) Late reactivation of Chagas' disease presenting in a recipient as an expansive mass lesion in the brain after heart transplantation of chagasic myocardiopathy. J Heart Lung Transplant 26: 1091-1096.

82. Marcu CB, Beek AM, van Rossum AC (2007) Chagas' heart disease diagnosed on MRI: the importance of patient "geographic" history. Int J Cardiol 117: e58-60.

83. Merino FJ, Martinez-Ruiz R, Olabarrieta I, Merino P, Garcia-Bujalance S, et al. [Control of Chagas disease in pregnant Latin-American women and her children]. Rev Esp Quimioter 2013 Sep;26(3):253-60.

84. Moraes-Souza H, Ferreira-Silva MM [Control of transfusional transmission]. Rev Soc Bras Med Trop 44 Suppl 2: 64-67.

85. Munoz J, Portus M, Corachan M, Fumado V, Gascon J (2007) Congenital Trypanosoma cruzi infection in a non-endemic area. Trans R Soc Trop Med Hyg 101: 1161-1162.

86. Murcia L, Carrilero B, Munoz-Davila MJ, Thomas MC, Lopez MC, et al. Risk factors and primary prevention of congenital Chagas disease in a nonendemic country. Clin Infect Dis 2013 Feb;56(4):496-502 doi: 101093/cid/cis910 Epub 2012 Oct 24.

87. Murcia L, Carrilero B, Saura D, Iborra MA, Segovia M [Diagnosis and treatment of Chagas disease]. Enferm Infecc Microbiol Clin 2013 Feb;31 Suppl 1:26-34 doi: 101016/S0213-005X(13)70111-3.

88. Navarro M, Norman FF, Perez-Molina JA, Lopez-Velez R Benznidazole shortage makes chagas disease a neglected tropical disease in developed countries: data from Spain. Am J Trop Med Hyg 2012 Sep;87(3):489-90 doi: 104269/ajtmh201212-0080 Epub 2012 Jul 23.

89. Norman FF, Perez-Ayala A, Perez-Molina JA, Flores-Chavez M, Canavate C, et al. Lack of association between blood-based detection of Trypanosoma cruzi DNA and cardiac involvement in a non-endemic area. Ann Trop Med Parasitol 2011 Sep;105(6):425-30 doi: 101179/1364859411Y0000000033.

90. O'Brien SF, Scalia V, Goldman M, Fan W, Yi QL, et al. Evaluation of selective screening of donors for antibody to Trypanosoma cruzi: seroprevalence of donors who answer "no" to risk questions. Transfusion 2013 Apr 25 doi: 101111/trf12219.

91. Parker ER, Sethi A Chagas disease: coming to a place near you. Dermatol Clin 29: 53-62.

92. Pays JF (1998) [American human trypanosomiasis 90 years after its discovery by Carlos Chagas. I. Epidemiology and control]. Med Trop (Mars) 58: 391-402.

93. Pays JF (1999) [Human American trypanosomiasis 90 years after its discovery by Carlos Chagas. II--Clinical aspects, physiopathology, diagnosis and treatment]. Med Trop (Mars) 59: 79-94.

94. Pearlman JD (1983) Chagas' disease in northern California. No longer an endemic diagnosis. Am J Med 75: 1057-1060.

95. Perez de Ayala A, Perez-Molina JA, Norman F, Lopez-Velez R (2009) Chagasic cardiomyopathy in immigrants from Latin America to Spain. Emerg Infect Dis 15: 607-608.

96. Perez-Ayala A, Perez-Molina JA, Norman F, Monge-Maillo B, Faro MV, et al. Gastro-intestinal Chagas disease in migrants to Spain: prevalence and methods for early diagnosis. Ann Trop Med Parasitol 105: 25-29.

97. Perez-Molina JA, Norman F, Lopez-Velez R Chagas disease in non-endemic countries: epidemiology, clinical presentation and treatment. Curr Infect Dis Rep 2012 Jun;14(3):263-74 doi: 101007/s11908-012-0259-3.

98. Posada E, Pell C, Angulo N, Pinazo MJ, Gimeno F, et al. Bolivian migrants with Chagas disease in Barcelona, Spain: a qualitative study of dietary changes and digestive problems. Int Health 2011 Dec;3(4):289-94 doi: 101016/jinhe201109005.

99. Raccurt CP (1999) Acute Chagasic cardiopathy in French Guiana: a re-emergent hazard linked to ecological change? Ann Trop Med Parasitol 93: 183-184.

100. Ramirez JD, Guhl F, Messenger LA, Lewis MD, Montilla M, et al. Contemporary cryptic sexuality in Trypanosoma cruzi. Mol Ecol 2012 Sep;21(17):4216-26 doi: 101111/j1365-294X201205699x Epub 2012 Jul 9.

101. Ramos JM, Gonzalez-Alcaide G, Gascon J, Gutierrez F Mapping of Chagas disease research: analysis of publications in the period between 1940 and 2009. Rev Soc Bras Med Trop 2011 Nov-Dec;44(6):708-16 Epub 2011 Nov 21.

102. Riera C, Guarro A, Kassab HE, Jorba JM, Castro M, et al. (2006) Congenital transmission of Trypanosoma cruzi in Europe (Spain): a case report. Am J Trop Med Hyg 75: 1078-1081.

103. Rodriguez-Guardado A, Tuset M, Asensi V, Miro JM [Human immunodeficiency virus and Chagas disease coinfection treated successfully with benznidazole and a raltegravir-based antiretroviral regimen: a case report]. Med Clin (Barc) 137: 278-279.

104. Sabino EC, Salles NA, Sarr M, Barreto AM, Oikawa M, et al. Enhanced classification of Chagas serologic results and epidemiologic characteristics of seropositive donors at three large blood centers in Brazil. Transfusion 50: 2628-2637.

105. Salamanca-Dejour D, Blanchet D, Aznar C, La Ruche G, Jeannel D, et al. [Chagas disease (American trypanosomiasis) in France]. Med Mal Infect 2012 Aug;42(8):344-8 doi: 101016/jmedmal201101006 Epub 2012 May 14.

106. Sarkar S, Strutz SE, Frank DM, Rivaldi CL, Sissel B, et al. Chagas disease risk in Texas. PLoS Negl Trop Dis 4.

107. Schmunis GA (1991) Trypanosoma cruzi, the etiologic agent of Chagas' disease: status in the blood supply in endemic and nonendemic countries. Transfusion 31: 547-557.

108. Schmunis GA (1999) [Risk of Chagas disease through transfusions in the Americans]. Medicina (B Aires) 59 Suppl 2: 125-134.

109. Schmunis GA (2007) Epidemiology of Chagas disease in non-endemic countries: the role of international migration. Mem Inst Oswaldo Cruz 102 Suppl 1: 75-85.

110. Schmunis GA, Yadon ZE Chagas disease: a Latin American health problem becoming a world health problem. Acta Trop 115: 14-21.

111. Theis JH (1990) Latin American immigrants--blood donation and Trypanosoma cruzi transmission. Am Heart J 120: 1483-1484.

112. Valerio L, Roure S, Sabria M, Balanzo X, Valles X, et al. Clinical, electrocardiographic and echocardiographic abnormalities in Latin American migrants with newly diagnosed Chagas disease 2005-2009, Barcelona, Spain. Euro Surveill 16.

113. Verani JR, Montgomery SP, Schulkin J, Anderson B, Jones JL Survey of obstetrician-gynecologists in the United States about Chagas disease. Am J Trop Med Hyg 83: 891-895.

114. Verani JR, Seitz A, Gilman RH, LaFuente C, Galdos-Cardenas G, et al. (2009) Geographic variation in the sensitivity of recombinant antigen-based rapid tests for chronic Trypanosoma cruzi infection. Am J Trop Med Hyg 80: 410-415.

115. Vieira GO, Maguire J, Bittencourt AL, Fontes JA (1983) [Congenital Chagas' disease. Report of a case with cerebral palsy]. Rev Inst Med Trop Sao Paulo 25: 305-309.

116. Villa A, Gutierrez C, Gracia E, Moreno B, Chacon G, et al. (2008) Presence of Trypanosoma theileri in Spanish Cattle. Ann N Y Acad Sci 1149: 352-354.

117. Wallace JA, Miller L, Beavis A, Baptista CA Chagas disease: a proposal for testing policy for solid-organ transplant in the United States. Prog Transplant 2013 Sep;23(3):272-7 doi: 107182/pit2013712.

118. Weinke T, Ueberreiter K, Alexander M (1988) Cardiac morbidity due to Chagas' disease in a rural community in Bolivia. Epidemiol Infect 101: 655-660.

119. Weissman AM (1994) Preventive health care and screening of Latin American immigrants in the United States. J Am Board Fam Pract 7: 310-323.

120. Wendel S, Biagini S (1995) Absence of serological surrogate markers for Trypanosoma-cruzi-infected blood donors. Vox Sang 69: 44-49.

121. Wendel S, Gonzaga AL (1993) Chagas' disease and blood transfusion: a New World problem? Vox Sang 64: 1-12.

122. Woody NC, Woody HB (1955) American trypanosomiasis (Chagas' disease); first indigenous case in the United States. J Am Med Assoc 159: 676-677.

123. Woody NC, Woody HB (1961) American trypanosomiasis. I. Clinical and epidemiologic background of Chagas' disease in the United States. J Pediatr 58: 568-580.

124. Woody NC, Woody HB (1974) Letter: Possible Chagas's disease in United States. N Engl J Med 290: 749-750.

125. Yadon ZE, Schmunis GA (2009) Congenital Chagas disease: estimating the potential risk in the United States. Am J Trop Med Hyg 81: 927-933.

126. Zou S, Stramer SL, Dodd RY Donor testing and risk: current prevalence, incidence, and residual risk of transfusion-transmissible agents in US allogeneic donations. Transfus Med Rev 26: 119-128.

**ARTICLES WITHOUT ABSTRACT AVAILABLE**

1. Bern C, Montgomery SP (2008) Recognizing and reducing the risks of Chagas disease in travelers. J Travel Med 15: 385; author reply 386.

2. Comeau P (2007) Canadian Blood Services to screen for Chagas disease. CMAJ 177: 242.

3. Crovato F, Rebora A (1997) Chagas' disease: a potential plague for Europe? Dermatology 195: 184-185.

4. Farrar WE, Jr., Kagan IG, Everton FD, Sellers TF, Jr. (1963) SEROLOGIC EVIDENCE OF HUMAN INFECTION WITH TRYPANOSOMA CRUZI IN GEORGIA. Am J Hyg 78: 166-172.

5. Hoff R, Todd CW, Maguire JH, Piesman J, Mott KE, et al. (1985) Serologic surveillance of Chagas' disease. Ann Soc Belg Med Trop 65 Suppl 1: 187-196.

6. Kirchhoff LV (1989) Is Trypanosoma cruzi a new threat to our blood supply? Ann Intern Med 111: 773-775.

7. Navarro M, Navaza B, Guionnet A, Lopez-Velez R Chagas disease in Spain: need for further public health measures. PLoS Negl Trop Dis 2012;6(12):e1962 doi: 101371/journalpntd0001962 Epub 2012 Dec 27.

8. Perez-Arellano JL [Chagas disease in Spain, 2012]: Rev Clin Esp. 2012 Jul;212(7):344-6. doi: 10.1016/j.rce.2012.04.010. Epub 2012 Jun 2.

9. Pihl T, Strand EA, Strand OA, Ohm OJ (2007) [Chagas disease in Norway]. Tidsskr Nor Laegeforen 127: 1820-1823.

10. Ramos JM, Pinargote H, Andreu M, Sastre J, Torrus D, et al. Prevalence of Trypanosoma cruzi infection in Latin American pregnant women and level of compliance of the Valencian Health Programme in the city of Alicante. Epidemiol Infect 2013 Aug 6:1-4.

11. Reyes PA (1992) Chagas' disease in the United States. Am Heart J 123: 1724.

12. Rodriguez-Morales AJ, Benitez JA, Tellez I, Franco-Paredes C (2008) Chagas disease screening among Latin American immigrants in non-endemic settings. Travel Med Infect Dis 6: 162-163.

13. Rodriguez-Morales AJ, Silvestre J, Cazorla-Perfetti DJ (2009) Chagas disease in Barcelona, Spain. Acta Trop 112: 86-87.

14. Sandahl K, Botero-Kleiven S, Hellgren U [Chagas' disease in Sweden--great need of guidelines for testing. Probably hundreds of seropositive cases, only a few known]. Lakartidningen 2011 Nov 16-22;108(46):2368-71.

15. Trillo Urrutia L, Garces Jarque JM, Gris Martinez JM (2008) [Chagas disease: an emerging disease in Spain]. Rev Esp Anestesiol Reanim 55: 525-526.

16. Yaeger RG (1961) The present status of Chagas' disease in the United States. Bull Tulane Univ Med Fac 21: 9-13.

**STUDIES INCLUDED (OTHER SOURCES)**

1. Irueta Isusi A, Jarque Moyano M, Redondo Ruiz P, Perez Martin V (2012) [Imported Chagas disease: alarm in Europe... as it should be]. Aten Primaria 44: 115-116.

***STUDIES EXCLUDED AFTER FULL-TEXT SCREENING.***

**NON PREVALENCE STUDIES**

1. (2007) Blood donor screening for chagas disease--United States, 2006-2007. MMWR Morb Mortal Wkly Rep 56: 141-143.

2. Agapova M, Busch MP, Custer B Cost-effectiveness of screening the US blood supply for Trypanosoma cruzi. Transfusion 50: 2220-2232.

3. Assal A, Pelletier B, David B, Tiberghien P (2009) [Chagas disease screening in the blood donor population]. Bull Soc Pathol Exot 102: 291-294.

4. Barona-Vilar C (2013) Prevalence of Trypanosoma cruzi infection in Latin American pregnant women and level of compliance of the Valencian Health Programme in the city of Alicante: a reply. Epidemiol Infect 2013 Aug 6:1-2.

5. Barrett VJ, Leiby DA, Odom JL, Otani MM, Rowe JD, et al. (1997) Negligible prevalence of antibodies against Trypanosoma cruzi among blood donors in the southeastern United States. Am J Clin Pathol 108: 499-503.

6. Bart A, Hodiamont CJ, Grobusch MP, van den Brink RB, Smout AJ, et al. (2011) [Chagas disease in the Netherlands: an estimate of the number of patients]. Ned Tijdschr Geneeskd 155: A3170.

7. Basile L, Jansa JM, Carlier Y, Salamanca DD, Angheben A, et al. (2011) Chagas disease in European countries: the challenge of a surveillance system. Euro Surveill 16.

8. Basile L, Oliveira I, Ciruela P, Plasencia A (2011) The current screening programme for congenital transmission of Chagas disease in Catalonia, Spain. Euro Surveill 16.

9. Benjamin RJ, Stramer SL, Leiby DA, Dodd RY, Fearon M, et al. (2012) Trypanosoma cruzi infection in North America and Spain: evidence in support of transfusion transmission. Transfusion 2012 Sep;52(9):1913-21; quiz 1912 doi: 101111/j1537-2995201103554x Epub 2012 Feb 10.

10. Bern C, Montgomery SP (2008) Recognizing and reducing the risks of Chagas disease in travelers. J Travel Med 15: 385; author reply 386.

11. Bern C, Montgomery SP (2009) An estimate of the burden of Chagas disease in the United States. Clin Infect Dis 49: e52-54.

12. Bern C, Montgomery SP, Katz L, Caglioti S, Stramer SL (2008) Chagas disease and the US blood supply. Curr Opin Infect Dis 21: 476-482.

13. Brutus L, Santalla JA, Salas NA, Schneider D, Chippaux JP (2009) [Screening for congenital infection by Trypanosoma cruzi in France]. Bull Soc Pathol Exot 102: 300-309.

14. Buekens P, Almendares O, Carlier Y, Dumonteil E, Eberhard M, et al. (2008) Mother-to-child transmission of Chagas' disease in North America: why don't we do more? Matern Child Health J 12: 283-286.

15. Burkholder JE, Allison TC, Kelly VP (1980) Trypanosoma cruzi (Chagas) (Protozoa: Kinetoplastida) in invertebrate, reservoir, and human hosts of the lower Rio Grande valley of Texas. J Parasitol 66: 305-311.

16. Cantey PT, Stramer SL, Townsend RL, Kamel H, Ofafa K, et al. (2012) The United States Trypanosoma cruzi Infection Study: evidence for vector-borne transmission of the parasite that causes Chagas disease among United States blood donors. Transfusion 2012 Sep;52(9):1922-30 doi: 101111/j1537-2995201203581x Epub 2012 Mar 8.

17. Carlier Y (2007) [Congenital Chagas disease: from the laboratory to public health]. Bull Mem Acad R Med Belg 162: 409-416; discussion 416-407.

18. Carlier Y (2011) Globalization of Chagas disease (American trypanosomiasis): the situation in Europe and Belgium. Bull Mem Acad R Med Belg 166: 347-355; discussion 356-347.

19. Cortez J, Ramos E, Valente C, Seixas J, Vieira A (2012) [Global expression of Chagas disease - emerging opportunities and impact in Portugal]. Acta Med Port 2012 Sep-Oct;25(5):332-9 Epub 2012 Nov 12.

20. Crovato F, Rebora A (1997) Chagas' disease: a potential plague for Europe? Dermatology 195: 184-185.

21. Custer B, Agapova M, Bruhn R, Cusick R, Kamel H, et al. (2012) Epidemiologic and laboratory findings from 3 years of testing United States blood donors for Trypanosoma cruzi. Transfusion 2012 Sep;52(9):1901-11 doi: 101111/j1537-2995201203569x Epub 2012 Feb 17.

22. Dejour Salamanca D, La Ruche G, Tarantola A, Degail MA, Jeannel D, et al. (2009) [Chagas disease in France: estimated number of infected persons and cardiac diseases in 2009, by risk groups]. Bull Soc Pathol Exot 102: 285-290.

23. Di Girolamo C, Bodini C, Marta BL, Ciannameo A, Cacciatore F (2011) Chagas disease at the crossroad of international migration and public health policies: why a national screening might not be enough. Euro Surveill 16.

24. Di Girolamo C, Marta BL, Ciannameo A, Cacciatore F, Balestra GL, et al. (2010) [Chagas disease in a non endemic country: a study in the district of Bologna (Italy). Multidisciplinary analysis of the disease in the Latin American migrant population]. Ann Ig 22: 431-445.

25. Farrar WE, Jr., Kagan IG, Everton FD, Sellers TF, Jr. (1963) SEROLOGIC EVIDENCE OF HUMAN INFECTION WITH TRYPANOSOMA CRUZI IN GEORGIA. Am J Hyg 78: 166-172.

26. Galel SA, Kirchhoff LV (1996) Risk factors for Trypanosoma cruzi infection in California blood donors. Transfusion 36: 227-231.

27. Hall CA, Polizzi C, Yabsley MJ, Norton TM (2007) Trypanosoma cruzi prevalence and epidemiologic trends in lemurs on St. Catherines Island, Georgia. J Parasitol 93: 93-96.

28. Jackson Y, Chappuis F (2011) Chagas disease in Switzerland: history and challenges. Euro Surveill 16.

29. Jackson Y, Pinto A, Pett S (2013) Chagas disease in Australia and New Zealand: risks and needs for public health interventions. Trop Med Int Health 2013 Dec 3 doi: 101111/tmi12235.

30. Kirchhoff LV (1989) Is Trypanosoma cruzi a new threat to our blood supply? Ann Intern Med 111: 773-775.

31. Lescure FX, Le Loup G, Freilij H, Develoux M, Paris L, et al. (2010) Chagas disease: changes in knowledge and management. Lancet Infect Dis 10: 556-570.

32. Navarro M, Navaza B, Guionnet A, Lopez-Velez R Chagas disease in Spain: need for further public health measures. PLoS Negl Trop Dis 2012;6(12):e1962 doi: 101371/journalpntd0001962 Epub 2012 Dec 27.

33. Nowicki MJ, Chinchilla C, Corado L, Matsuoka L, Selby R, et al. (2006) Prevalence of antibodies to Trypanosoma cruzi among solid organ donors in Southern California: a population at risk. Transplantation 81: 477-479.

34. O'Brien SF, Chiavetta JA, Fan W, Xi G, Yi QL, et al. (2008) Assessment of a travel question to identify donors with risk of Trypanosoma cruzi: operational validity and field testing. Transfusion 48: 755-761.

35. O'Brien SF, Scalia V, Goldman M, Fan W, Yi QL, et al. (2013) Selective testing for Trypanosoma cruzi: the first year after implementation at Canadian Blood Services. Transfusion 2013 Aug;53(8):1706-13 doi: 101111/j1537-2995201203950x Epub 2012 Nov 12.

36. Perez-Arellano JL [Chagas disease in Spain, 2012]: Rev Clin Esp. 2012 Jul;212(7):344-6. doi: 10.1016/j.rce.2012.04.010. Epub 2012 Jun 2.

37. Perez-Lopez FR, Chedraui P (2010) Chagas disease in pregnancy: a non-endemic problem in a globalized world. Arch Gynecol Obstet 282: 595-599.

38. Pihl T, Strand EA, Strand OA, Ohm OJ (2007) [Chagas disease in Norway]. Tidsskr Nor Laegeforen 127: 1820-1823.

39. Ramos JM, Pinargote H, Andreu M, Sastre J, Torrus D, et al. Prevalence of Trypanosoma cruzi infection in Latin American pregnant women and level of compliance of the Valencian Health Programme in the city of Alicante. Epidemiol Infect 2013 Aug 6:1-4.

40. Ramos-Rincon JM, Milla-Jover A, Rodriguez-Diaz JC, Gutierrez-Rodero F (2012) [Evaluation of the grade of application of the recommendations of screening for Chagas disease in pregnant women]: Rev Clin Esp. 2012 Jul;212(7):366-8. doi: 10.1016/j.rce.2012.02.004. Epub 2012 Apr 10.

41. Rodriguez-Morales AJ, Silvestre J, Cazorla-Perfetti DJ (2009) Chagas disease in Barcelona, Spain. Acta Trop 112: 86-87.

42. Sandahl K, Botero-Kleiven S, Hellgren U [Chagas' disease in Sweden--great need of guidelines for testing. Probably hundreds of seropositive cases, only a few known]. Lakartidningen 2011 Nov 16-22;108(46):2368-71.

43. Santiago B, Blazquez D, Lopez G, Sainz T, Munoz M, et al. (2011) [Serological profile of immigrant pregnant women against HIV, HBV, HCV, rubella, Toxoplasma gondii, Treponema pallidum, and Trypanosoma cruzi]. Enferm Infecc Microbiol Clin 30: 64-69.

44. Shulman IA, Appleman MD, Saxena S, Hiti AL, Kirchhoff LV (1997) Specific antibodies to Trypanosoma cruzi among blood donors in Los Angeles, California. Transfusion 37: 727-731.

45. Tobler LH, Contestable P, Pitina L, Groth H, Shaffer S, et al. (2007) Evaluation of a new enzyme-linked immunosorbent assay for detection of Chagas antibody in US blood donors. Transfusion 47: 90-96.

46. Trillo Urrutia L, Garces Jarque JM, Gris Martinez JM (2008) [Chagas disease: an emerging disease in Spain]. Rev Esp Anestesiol Reanim 55: 525-526.

47. Valerio-Sallent L, Roure S, Basile L, Ballesteros LA, Sabria M, et al. (2012) [A clinical and epidemiological study of the Trypanosoma cruzi infected population in the north metropolitan area of Barcelona]. Rev Clin Esp 2012 Jul;212(7):329-36 doi: 101016/jrce201203017 Epub 2012 May 18.

48. Voelker R (2012) Congenital Chagas disease reported in United States: JAMA. 2012 Aug 1;308(5):443. doi: 10.1001/jama.2012.9468.

49. Wilson LS, Ramsey JM, Koplowicz YB, Valiente-Banuet L, Motter C, et al. (2008) Cost-effectiveness of implementation methods for ELISA serology testing of Trypanosoma cruzi in California blood banks. Am J Trop Med Hyg 79: 53-68.

50. Winkler MA, Brashear RJ, Hall HJ, Schur JD, Pan AA (1995) Detection of antibodies to Trypanosoma cruzi among blood donors in the southwestern and western United States. II. Evaluation of a supplemental enzyme immunoassay and radioimmunoprecipitation assay for confirmation of seroreactivity. Transfusion 35: 219-225.

51. Yaeger RG (1961) The present status of Chagas' disease in the United States. Bull Tulane Univ Med Fac 21: 9-13.

52. Zaniello BA, Kessler DA, Vine KM, Grima KM, Weisenberg SA (2012) Seroprevalence of Chagas infection in the donor population. PLoS Negl Trop Dis 2012;6(7):e1771 doi: 101371/journalpntd0001771 Epub 2012 Jul 31.

**HOSPITAL-BASED STUDIES**

1. Munoz J, Gomez i Prat J, Gallego M, Gimeno F, Trevino B, et al. (2009) Clinical profile of Trypanosoma cruzi infection in a non-endemic setting: immigration and Chagas disease in Barcelona (Spain). Acta Trop 111: 51-55.

2. Perez-Ayala A, Perez-Molina JA, Norman F, Navarro M, Monge-Maillo B, et al. (2010) Chagas disease in Latin American migrants: a Spanish challenge. Clin Microbiol Infect 17: 1108-1113.

3. Perez-Molina JA, Perez-Ayala A, Parola P, Jackson Y, Odolini S, et al. (2011) EuroTravNet: imported Chagas disease in nine European countries, 2008 to 2009. Euro Surveill 16.

4. Ramos JM, Torrus D, Amador C, Jover F, Perez-Chacon F, et al. (2012) Multicenter epidemiological and clinical study on imported Chagas diseases in Alicante, Spain. Pathog Glob Health 2012 Oct;106(6):340-5 doi: 101179/2047773212Y0000000039.

5. Rodriguez-Guardado A, Asensi Alvarez V, Rodriguez Perez M, Mejuto Alvarez P, Flores-Chavez M, et al. (2010) Screening for Chagas' disease in HIV-positive immigrants from endemic areas. Epidemiol Infect 139: 539-543.

6. Rodriguez-Guardado A, Rodriguez M, Alonso P, Seco C, Flores-Chavez M, et al. (2009) Serological screening of Chagas disease in an immigrant population in Asturias, Spain proceeding from Chagas-endemic areas. Scand J Infect Dis 41: 774-776.

7. Salvador F, Trevino B, Sulleiro E, Pou D, Sanchez-Montalva A, et al. (2013) Trypanosoma cruzi infection in a non-endemic country: epidemiological and clinical profile. Clin Microbiol Infect 2013 Nov 7 doi: 101111/1469-069112443.

**INFORMATION ON COUNTRY NOT PROVIDED**

1. Assal A, Corbi C (2011) [Chagas disease and blood transfusion: an emerging issue in non-endemic countries]. Transfus Clin Biol 18: 286-291.

2. Comeau P (2007) Canadian Blood Services to screen for Chagas disease. CMAJ 177: 242.

3. Di Pentima MC, Hwang LY, Skeeter CM, Edwards MS (1999) Prevalence of antibody to Trypanosoma cruzi in pregnant Hispanic women in Houston. Clin Infect Dis 28: 1281-1285.

4. Flores-Chavez MD, Merino FJ, Garcia-Bujalance S, Martin-Rabadan P, Merino P, et al. (2011) Surveillance of Chagas disease in pregnant women in Madrid, Spain, from 2008 to 2010. Euro Surveill 16.

5. Hoff R, Todd CW, Maguire JH, Piesman J, Mott KE, et al. (1985) Serologic surveillance of Chagas' disease. Ann Soc Belg Med Trop 65 Suppl 1: 187-196.

6. Kerleguer A, Massard S, Janus G, Joussemet M (2007) [Chagas disease: screening tests evaluation in a blood military center, prevalence in the French Army]. Pathol Biol (Paris) 55: 534-538.

7. Kessler DA, Shi PA, Avecilla ST, Shaz BH (2013) Results of lookback for Chagas disease since the inception of donor screening at New York Blood Center. Transfusion 2013 May;53(5):1083-7 doi: 101111/j1537-2995201203856x Epub 2012 Aug 15.

8. Kitchen AD, Hewitt PE, Chiodini PL (2012) The early implementation of Trypanosoma cruzi antibody screening of donors and donations within England: preempting a problem. Transfusion 2012 Sep;52(9):1931-9 doi: 101111/j1537-2995201203599x Epub 2012 Mar 13.

9. Leiby DA, Fucci MH, Stumpf RJ (1999) Trypanosoma cruzi in a low- to moderate-risk blood donor population: seroprevalence and possible congenital transmission. Transfusion 39: 310-315.

10. Leiby DA, Herron RM, Jr., Read EJ, Lenes BA, Stumpf RJ (2002) Trypanosoma cruzi in Los Angeles and Miami blood donors: impact of evolving donor demographics on seroprevalence and implications for transfusion transmission. Transfusion 42: 549-555.

11. Leiby DA, Read EJ, Lenes BA, Yund AJ, Stumpf RJ, et al. (1997) Seroepidemiology of Trypanosoma cruzi, etiologic agent of Chagas' disease, in US blood donors. J Infect Dis 176: 1047-1052.

12. Lescure FX, Paris L, Elghouzzi MH, Le Loup G, Develoux M, et al. (2009) [Experience of targeted screening of Chagas disease in Ile-de-France]. Bull Soc Pathol Exot 102: 295-299.

13. Otero S, Sulleiro E, Molina I, Espiau M, Suy A, et al. (2012) Congenital transmission of Trypanosoma cruzi in non-endemic areas: evaluation of a screening program in a tertiary care hospital in Barcelona, Spain. Am J Trop Med Hyg 2012 Nov;87(5):832-6 doi: 104269/ajtmh201212-0152 Epub 2012 Sep 17.

14. Paris L, Touafek F, Elghouzzi MH, Cherif S, Mazier D (2009) [Chagas disease in chronic phase outside the endemic area. The diagnostic tools]. Bull Soc Pathol Exot 102: 319-325.

15. Rodriguez-Morales AJ, Benitez JA, Tellez I, Franco-Paredes C (2008) Chagas disease screening among Latin American immigrants in non-endemic settings. Travel Med Infect Dis 6: 162-163.

16. Salvador F, Molina I, Sulleiro E, Burgos J, Curran A, et al. (2013) Tropical diseases screening in immigrant patients with human immunodeficiency virus infection in Spain. Am J Trop Med Hyg 2013 Jun;88(6):1196-202 doi: 104269/ajtmh12-0714 Epub 2013 Mar 18.

**STUDIES NOT CONDUCTED IN EUROPE**

1. Arena R, Mathews CE, Kim AY, Lenz TE, Southern PM (2011) Prevalence of antibody to Trypanosoma cruzi in Hispanic-surnamed patients seen at Parkland Health & Hospital System, Dallas, Texas. BMC Res Notes 4: 132.

2. Kerndt PR, Waskin HA, Kirchhoff LV, Steurer F, Waterman SH, et al. (1991) Prevalence of antibody to Trypanosoma cruzi among blood donors in Los Angeles, California. Transfusion 31: 814-818.

3. Kirchhoff LV, Gam AA, Gilliam FC (1987) American trypanosomiasis (Chagas' disease) in Central American immigrants. Am J Med 82: 915-920.

4. Reyes PA (1992) Chagas' disease in the United States. Am Heart J 123: 1724.

5. Steele LS, MacPherson DW, Kim J, Keystone JS, Gushulak BD (2007) The sero-prevalence of antibodies to trypanosoma cruzi in Latin American refugees and immigrants to Canada. J Immigr Minor Health 9: 43-47.

**STUDY WITH THE DATA BASE ALREADY INCLUDED IN ANOTHER STUDY**

1. Ramos JM, Milla A, Sanchez V, Verges M, Toro C, et al. (2009) [Prenatal screening for Trypanosoma cruzi and human T lymphotropic virus types 1 and 2 in pregnant Latin American women]. Enferm Infecc Microbiol Clin 27: 165-167.

**STUDIES THAT DID NOT FULLFILL THE WHO CRITERIA FOR *T.CRUZI* DIAGNOSIS**

1. Irueta Isusi A, Jarque Moyano M, Redondo Ruiz P, Perez Martin V (2012) [Imported Chagas disease: alarm in Europe... as it should be]. Aten Primaria 44: 115-116.

2. Jackson Y, Myers C, Diana A, Marti HP, Wolff H, et al. (2009) Congenital transmission of Chagas disease in Latin American immigrants in Switzerland. Emerg Infect Dis 15: 601-603.

**STUDIES INCLUDED IN THE META-ANALYSIS**

1. Angheben A, Anselmi M, Gobbi F, Marocco S, Monteiro G, et al. (2011) Chagas disease in Italy: breaking an epidemiological silence. Euro Surveill 16.

2. Avila Arzanegui O, Liendo Arenaza P, Martinez Indart L, Martinez Astorkiza T, Pocheville Guruceta MI, et al. (2013) [Prevalence of Trypanosoma cruzi infection and vertical transmission in Latin-American pregnant women in a health area of Biscay]. Enferm Infecc Microbiol Clin 2013 Apr;31(4):210-6 doi: 101016/jeimc201201029 Epub 2012 May 21.

3. Barona-Vilar C, Gimenez-Marti MJ, Fraile T, Gonzalez-Steinbauer C, Parada C, et al. (2012) Prevalence of Trypanosoma cruzi infection in pregnant Latin American women and congenital transmission rate in a non-endemic area: the experience of the Valencian Health Programme (Spain). Epidemiol Infect 2012 Oct;140(10):1896-903 doi: 101017/S0950268811002482 Epub 2011 Dec 1.

4. El Ghouzzi MH, Boiret E, Wind F, Brochard C, Fittere S, et al. (2010) Testing blood donors for Chagas disease in the Paris area, France: first results after 18 months of screening. Transfusion 50: 575-583.

5. Frank M, Hegenscheid B, Janitschke K, Weinke T (1997) Prevalence and epidemiological significance of Trypanosoma cruzi infection among Latin American immigrants in Berlin, Germany. Infection 25: 355-358.

6. Gabrielli S, Girelli G, Vaia F, Santonicola M, Fakeri A, et al. (2013) Surveillance of Chagas disease among at-risk blood donors in Italy: preliminary results from Umberto I Polyclinic in Rome. Blood Transfus 2013 Oct;11(4):558-62 doi: 102450/20130055-13 Epub 2013 Oct 2.

7. Jackson Y, Getaz L, Wolff H, Holst M, Mauris A, et al. (2010) Prevalence, clinical staging and risk for blood-borne transmission of Chagas disease among Latin American migrants in Geneva, Switzerland. PLoS Negl Trop Dis 4: e592.

8. Lucas RM, Barba MC (2009) [Prevalence of american trypanosomiasis in pregnant women from a health area of Valencia, Spain: 2005-2007]. Rev Esp Salud Publica 83: 543-555.

9. Martinez de Tejada B, Jackson Y, Paccolat C, Irion O (2009) [Congenital Chagas disease in Geneva: diagnostic and clinical aspects]. Rev Med Suisse 5: 2091-2092, 2094-2096.

10. Munoz J, Coll O, Juncosa T, Verges M, del Pino M, et al. (2009) Prevalence and vertical transmission of Trypanosoma cruzi infection among pregnant Latin American women attending 2 maternity clinics in Barcelona, Spain. Clin Infect Dis 48: 1736-1740.

11. Munoz-Vilches MJ, Salas J, Cabezas T, Metz D, Vazquez J, et al. (2012) [Chagas screening in pregnant Latin-American women. Experience in Poniente Almeriense (Almeria, Spain)]. Enferm Infecc Microbiol Clin 2012 Aug;30(7):380-2 doi: 101016/jeimc201111012 Epub 2012 Jan 25.

12. Navarro M, Perez-Ayala A, Guionnet A, Perez-Molina JA, Navaza B, et al. (2011) Targeted screening and health education for Chagas disease tailored to at-risk migrants in Spain, 2007 to 2010. Euro Surveill 16.

13. Paricio-Talayero JM, Benlloch-Muncharaz MJ, Collar-del-Castillo JI, Rubio-Soriano A, Serrat-Perez C, et al. (2008) [Epidemiological surveillance of vertically-transmitted Chagas disease at three maternity hospitals in the Valencian Community]. Enferm Infecc Microbiol Clin 26: 609-613.

14. Piron M, Verges M, Munoz J, Casamitjana N, Sanz S, et al. (2008) Seroprevalence of Trypanosoma cruzi infection in at-risk blood donors in Catalonia (Spain). Transfusion 48: 1862-1868.

15. Ramos JM, Milla A, Rodriguez JC, Lopez-Chejade P, Flores M, et al. (2011) Chagas disease in Latin American pregnant immigrants: experience in a non-endemic country. Arch Gynecol Obstet 285: 919-923.

16. Ramos JM, Ponce Y, Gallegos I, Flores-Chavez M, Canavate C, et al. (2012) Trypanosoma cruzi infection in Elche (Spain): comparison of the seroprevalence in immigrants from Paraguay and Bolivia. Pathog Glob Health 2012 May;106(2):102-6 doi: 101179/2047773212Y0000000013.

17. Roca C, Pinazo MJ, Lopez-Chejade P, Bayo J, Posada E, et al. (2011) Chagas disease among the Latin American adult population attending in a primary care center in Barcelona, Spain. PLoS Negl Trop Dis 5: e1135.

18. Soriano Arandes A, Munoz Gutierrez J, Verges Navarro M, Castells Domenech C, Portus Vinyeta M, et al. (2009) Prevalence of Chagas disease in the Latin American immigrant population in a primary health centre in Barcelona (Spain). Acta Trop 112: 228-230.
