# Supplementary material for: Prevalence of Chagas Disease in Latin-American Migrants Living in Europe: A Systematic Review and Meta-analysis
Source: PLoS Negl Trop Dis. 2015 Feb 13;9(2):e0003540. doi: 10.1371/journal.pntd.0003540 (PMC4332678; doi:10.1371/journal.pntd.0003540)
Supplement: S1 Diagram — PRISMA Flow diagram. (DOC) [file pntd.0003540.s003.doc]

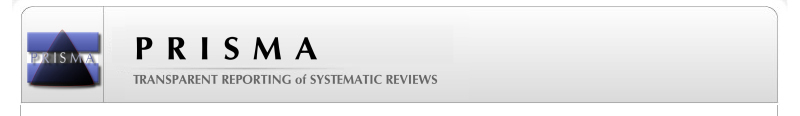
**PRISMA 2009 Flow Diagram**

**Screening**

**Included**

**Eligibility**

**Identification**

Records identified through database searching
(n =1296 )

Additional records identified through other sources
(n = 1 )

Records after duplicates removed
(n =0 )

Records screened
(n =1297 )

Records excluded
(n = 1070 )

Full-text articles assessed for eligibility
(n = 101 )

Full-text articles excluded, with reasons
(n =83 )

Studies included in qualitative synthesis
(n =18)

Studies included in quantitative synthesis (meta-analysis)
(n =18 )
